# Clinically Applicable Inhibitors Impacting Genome Stability

**DOI:** 10.3390/molecules23051166

**Published:** 2018-05-13

**Authors:** Anu Prakash, Juan F. Garcia-Moreno, James A. L. Brown, Emer Bourke

**Affiliations:** 1 Discipline of Pathology, Lambe Institute for Translational Research, School of Medicine, National University of Ireland Galway, H91 YR71 Galway, Ireland; A.PRAKASH2@nuigalway.ie; 2 Discipline of Surgery, Lambe Institute for Translational Research, School of Medicine, National University of Ireland Galway, H91 YR71 Galway, Ireland; juan_fernandez93@hotmail.com

**Keywords:** clustering, HR, NHEJ, clinical, trial, centrosome, amplification, chromatin, DSB, inhibitor

## Abstract

Advances in technology have facilitated the molecular profiling (genomic and transcriptomic) of tumours, and has led to improved stratification of patients and the individualisation of treatment regimes. To fully realize the potential of truly personalised treatment options, we need targeted therapies that precisely disrupt the compensatory pathways identified by profiling which allow tumours to survive or gain resistance to treatments. Here, we discuss recent advances in novel therapies that impact the genome (chromosomes and chromatin), pathways targeted and the stage of the pathways targeted. The current state of research will be discussed, with a focus on compounds that have advanced into trials (clinical and pre-clinical). We will discuss inhibitors of specific DNA damage responses and other genome stability pathways, including those in development, which are likely to synergistically combine with current therapeutic options. Tumour profiling data, combined with the knowledge of new treatments that affect the regulation of essential tumour signalling pathways, is revealing fundamental insights into cancer progression and resistance mechanisms. This is the forefront of the next evolution of advanced oncology medicine that will ultimately lead to improved survival and may, one day, result in many cancers becoming chronic conditions, rather than fatal diseases.

## 1. Introduction

Improved treatments for cancer are moving rapidly beyond “one size fits all” treatment regimes to precision (or personalised) medicine [1,2,3,4]. This approach involves profiling the patient or tumour (genomic or transcriptomics), which allows dysregulated pathways to be categorised and specific targeted treatments to be selected and implemented, with the aim of improved targeted killing of cancer cells. Targeted treatment-based approaches require specific targeted therapeutics to be developed and their mechanisms of action understood.

**Figure 1:**
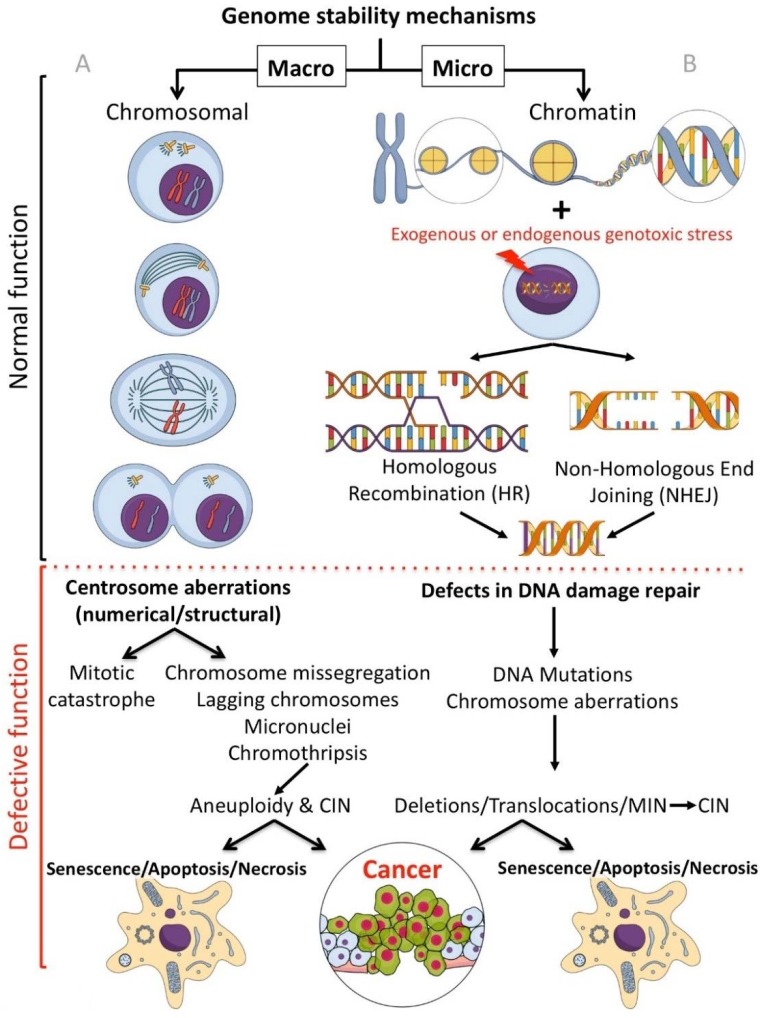
**Genome stability mechanisms**. (**A**). **Macro**—chromosomal integrity protected by centrosomal pathways. CIN: chromosomal instability. (**B**). **Micro**—DNA damage response mechanisms protecting chromatin from DNA double strand breaks. Homologous Recombination (HR) utilizes the undamaged DNA template (through strand invasion) allowing faithful reproduction of the original sequence. Non-Homologous End Joining (NHEJ) modifies and re-ligates broken DNA ends without consideration of the original sequence, generating mutations (deletions or insertions). MIN: microsatellite instability.

This review discusses current and potential future targeted therapeutics that effect genomic stability (inducing genomic instability), either targeting the genome at the macro (chromosome) or micro (chromatin) level (Figure 1).

Genomic instability can be caused through a number of independent, and at times related, mechanisms. Chromosomal instability (CIN) is the term labelling changes in chromosome structure and number, and is one of the most common forms of genomic instability found in human cancers. Microsatellite instability (MSI or MIN) is characterised by genetic hypermutability (a growth or reduction of oligonucleotide repeats in microsatellite sequences) resulting from impaired DNA repair mechanisms (reviewed in [5,6]). Genomic instability can be induced through defects (such as downregulation/mutation of key genes/proteins) in either the homologous recombination (HR), or the error-prone (intrinsically mutagenic) Non-Homologous End Joining (NHEJ) DNA repair pathways. HR is a slow process (hours) that utilizes the undamaged DNA template (through strand invasion) to allow faithful reproduction of the original sequence. NHEJ is a fast process (10 s of minutes) that modifies the broken DNA ends and joins them together without the original sequence as a template, as such, generating mutations (deletions or insertions) [7]. Genetic defects affecting the HR pathways result in reliance on the error prone NHEJ pathway for DNA repair, inducing/amplifying genome instability. Inhibitors targeting genome stability components, compounds targeting enzyme classes, descriptions of the drug molecular mechanisms of action, and the current or potential future clinical applications are reviewed.

### 1.1. Macro and Micro Targeting of Genome Stability Processes

In regard to inhibitors that target processes protecting the genome at the macro level, the focus of this section (Section 2) is on centrosome-affecting drugs. Centrosomes are multi-functional controllers of genome stability playing an essential role in protecting chromosomes [8,9,10]. Centrosomal aberrations, such as centrosome amplification (CA) can lead to mitotic catastrophe, aneuploidy, chromosome instability (CIN) or apoptosis (Figure 1A). 

Focusing on compounds targeting the chromatin (micro) level of genome protection, inhibitors of the most damaging DNA insult- the DNA double strand break (DSB) are detailed. Inhibiting specific DSB repair pathways (HR or NHEJ) in cancer cells can lead to targeted cell death (potentially sparing normal cells) [11] (Figure 1B). 

This review highlights small molecule inhibitors in use, being trailed, or exciting new potential clinical treatments that target cancer, by inhibiting essential pathways cancer cells rely on for survival. As pathway dysregulation in many cancers limits their choice of genome protective mechanisms, many tumour cells are vulnerable to targeted inhibition of these remaining genomic defence mechanisms. 

## 2. Small Molecule Inhibitors Targeted at the Chromosomal Level

The separation and correct positioning of (replicated) chromosomes during each cell cycle, late G2 and M phases, is a key process required to protect genomic integrity [8,9,10]. In mammalian cells, this process is coordinated by the centrosome, a subcellular organelle which forms the bipolar spindle during mitosis through microtubule organization, ensuring the equal distribution of chromosomes during mitosis (Figure 1A). Each centrosome has two centrioles embedded within a complex pericentriolar material (PCM). The PCM comprises hundreds of proteins, implicated in microtubule nucleation and mitotic assembly, kinetochore-microtubule attachments, DNA damage repair and cell cycle checkpoints [12]. This allows the centrosome to function as a multifunctional regulator of genome stability [9]. Due to this vital role, centrosome biogenesis/function is tightly regulated, with centrosome duplication occurring only once per cell cycle and is closely interconnected with cell cycle signalling/progression. Centrosome duplication results in two centrosomes, which migrate to opposite poles of the cell, orchestrating bipolar spindle formation for the faithful transmission of genetic material to the daughter cells. Anomalies in the centrosome cycle result in structural and numerical aberrations of the centrosome, causing genomic instability and aneuploidy, which have been shown drive tumourigenesis in vivo [13,14]. Importantly, centrosomal defects have long been associated with a wide range of solid and liquid tumours in humans [15]. Small molecules targeting centrosomal components have been developed and tested in vitro and in vivo, with several currently undergoing clinical trials, which will be reviewed below. 

### 2.1. Inhibitors Targeting Proteins Involved in Centrosome Duplication

**Figure 2:**
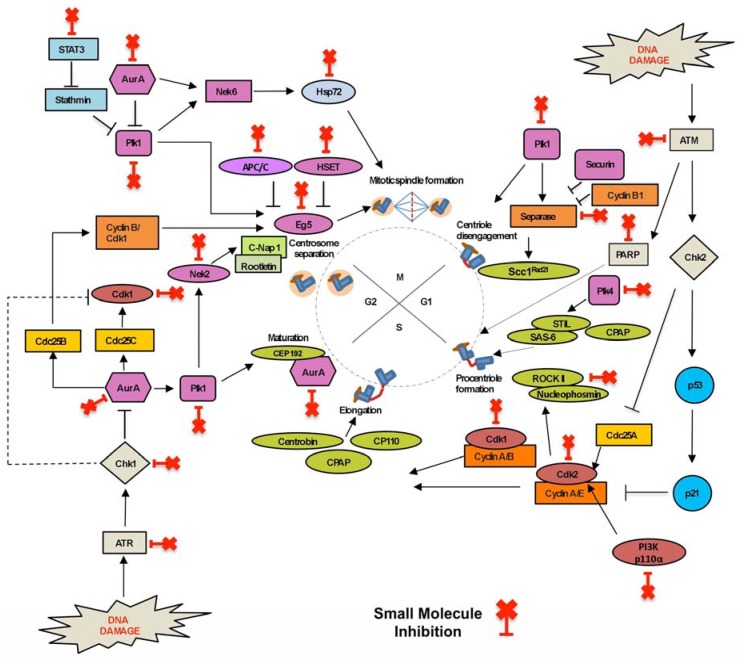
**Chromosomal stability mechanisms**. Centrosome duplication and segregation occurs in synchrony with the chromosome replication cycle. Proteins controlling progression through the phases of the centrosome and chromosome duplication cycles are key targets for inducing cytotoxicity in tumour cells. Small molecule inhibitor targets currently being investigated in pre-clinical and clinical trials are shown here in the context of the centrosome/cell cycle phases.

Centrosome number is regulated during the centrosome duplication cycle, and is a tightly controlled cellular event that is coupled with DNA replication and cell cycle progression [12]. Centrosome duplication is divided into four main stages: centriole disengagement, centriole duplication, centriole elongation and maturation, and centrosome separation (Figure 2). Proteins acting at each cell cycle phase or centrosome duplication cycle are key targets for small molecule inhibitors. Currently, inhibitors targeting the centrosome duplication cycle are in pre-clinical development or clinical trials and show promise as therapeutics (Table 1).

Sister chromatid and centriole separation is regulated by proteins expressed towards late mitosis, with some playing roles in both processes. Separase cleaves the cohesin subunit, Scc1^RAD21^, allowing sister chromatids to separate at the onset of anaphase during mitosis [16,49]. Separase also interacts with Polo-like kinase-1 (Plk1) to cleave Scc1^RAD21^ and initiates centriole disengagement [50]. In many human tumours separase is overexpressed (60% of breast cancers) and mislocalised [51]. Sepin-1 was designed as a novel non-competitive separase inhibitor, inhibiting separase enzymatic activity and inducing apoptosis in cancer cells [16,17]. Polo-like kinases (Plks) are serine/threonine protein kinases involved in diverse cellular processes and are crucial for mitotic progression, including centrosome maturation, mitotic entry, bipolar spindle formation, chromosome segregation, cytokinesis, and mitotic exit [52,53]. Plks are parallel activators of centriole disengagement, which is considered a licensing step for centriole duplication. Plk1 phosphorylates Pericentrin (PCNT), making it a target for Separase cleavage during mitotic exit, thus triggering centriole separation and regulating centriole duplication [52,54]. Plk1 co-regulates multiple mitotic events with another kinase, Aurora-A. Plk1 is phosphorylated (at pT210) by Aurora-A kinase, which in turn promotes the recruitment of Plk1 to centrosomes in late S-G2. Activated Plk1 localisation to the centrosome is a significant factor in the promotion of pro-centriole disengagement and maturation, leading to centriole duplication [55]. Plk1 also mediates the localisation of Eg5 kinesin, regulating centrosome separation [49,56]. Plk1 loss prevents centriole disengagement, and the subsequent centriole duplication. These key roles of Plk1 mean that Plk1 inhibitors are promising cancer therapeutics (reviewed in [18]). Plk1 small molecule inhibitors—BI 2536 (2-aminopyrimidine-containing ATP-competitive inhibitor), Volasertib (BI 6727) and GSK461364 (both ATP competitive molecules)—have shown important antitumour activity in xenograft models and are enrolled in clinical trials [19]. The ATP competitive inhibitor, ZK-thiazolidinone (TAL), is another promising Plk1 inhibitor that is currently in pre-clinical studies [57]. Dual action inhibitors co-inhibiting both Plk1 and Aurora-A kinases (discussed below) are a promising therapeutic option, which promote cell death by mitotic catastrophe in cancer cells [58].

Cell cycle progression, from G1 to S phase, initiates pro-centriole formation at the distal end of the mother centrioles. As the centrosome duplication cycle and cell cycle are linked, appropriate levels of active Cyclin–Cdk complexes are required for initiating the centrosome duplication cycle [56]. Importantly, nucleo-cytoplasmic shuttling of the Cyclin-A–Cdk2 and Cyclin-E–Cdk2 complexes promote initiation of centriole duplication and DNA synthesis. The Cyclin-E–Cdk2 complex phosphorylates several centrosomal proteins, such as Centrosomal protein (CP) 110 and nucleophosmin, regulating centrosomal activities [56,59,60]. While Cdk2 is a key regulator of both cell and centrosome cycles, it is not essential for centrosome duplication, as Cdk1 can compensate for Cdk2 in its absence [61]. Inhibition of Cdk2 can partially block centriole overduplication, thus maintaining genomic stability without affecting normal centriole duplication and cell cycle progression [62,63,64]. The Cdk2 inhibitors, SU9516 and Butyrolactone I (which inhibits centriole overduplication, promoting cell death in vitro), are in pre-clinical studies [22,23,24]. The Cdk2 inhibitor, Milciclib (PHA-848125 AC), and the non-specific Cdk2 inhibitors (targeting multiple Cdks, often 1, 2, 4 or 5) Flavopiridol (Alvocidib), R-547, SNS-032 and Roscovitine (CYC-202) are all enrolled in clinical trials (reviewed in [25]).

During the G1-S phase, Polo-like kinase 4 (Plk4; SAK in Drosophila) cooperates with Cyclin–Cdk complexes and recruits structural components required for the formation of pro-centrioles. Plk4 is considered the master regulator of centriole duplication, with an increase in Plk4 levels (and kinase activity) leading to extranumerary centrioles, whereas Plk4 depletion reduces centriole numbers [59]. Plk4 controls centriole number by phosphorylating substrate STIL (SCL/TAL1 interrupting locus) resulting in the recruitment of Sas6 to the pro-centriolar seeding site [65]. Subsequently, centriole elongation is governed by recruitment of Centrosomal P4.1–associated protein (CPAP), Centrobin and CP110. As a master regulator of centrosome duplication, Plk4 activity is tightly regulated, by trans-autophosphorylation, SCF (Slimb) E3 ubiquitin ligase degradation and KAT2A/B-mediated acetylation. Plk4 overexpression and overactivation are characteristics associated with many human cancers, and Plk4 is, therefore, considered a promising cancer target [66,67]. CFI-400945, a potent and selective Plk4 inhibitor is currently enrolled in phase I clinical trials in patients with relapsed and refractory acute myeloid leukaemia and myelodysplastic syndrome [30]. CFI-400945 blocks Plk4 kinase activity (both phosphorylation and trans-autophosphorylation) resulting in aberrant mitoses [30,66]. While overexpression of Plk4 causes centrosome amplification in cancer cells, insufficient amounts of Plk4 can trigger centriolar defects [68]. Therefore, achieving the correct dosage of Plk4 inhibitors is of critical importance (see discussion in Section 2.2). 

Centrosome maturation is coordinated by the kinase Aurora-A, which organises the recruitment of PCM proteins (including γ-TuRC and associated proteins). Aurora-A centrosomal recruitment is regulated by Plk1 (through phosphorylation). Importantly, Aurora-A overexpression induces centrosome amplification by concomitant tetraploidisation (not by excessive centrosome duplication) [31,33,69,70]. In contrast, the Aurora-B kinase acts as the catalytic component of the chromosomal passenger complex (CPC) and plays a key role in chromosome orientation, spindle assembly and cytokinesis. Overexpression of both Aurora kinases is associated with high tumour cell proliferation rates and poor patient prognosis, making them ideal targets for anticancer therapy [33]. Many small molecule inhibitors targeting the Aurora kinases are reversible ATP competitive inhibitors, which bind to the ATP-binding pocket via hydrogen bonding, and hydrophobic, aromatic and van der Waals interactions [33]. The orally-active Aurora-A inhibitor, ENMD-2076, has successfully completed phase II clinical trials in solid tumours [32]. The Aurora-A inhibitor, Alisertib (MLN8237), has shown promising efficacy in several solid tumours and has advanced to phase III clinical trials in T cell lymphoma patients [31]. Furthermore, newer Aurora-A inhibitors are entering phase I clinical trials, including MK-5108 (VX-689), KW-2449, XL228 and MLN8054 [33,34,35,36] (Table 1). Barasertib (AZD1152-HQPA) which selectively inhibits Aurora-B (over Aurora-A) has been widely studied in advanced solid tumours and haematological cancers, and is reportedly clinically effective in acute myeloid leukaemia (AML) patients [37]. The dual active inhibitors (targeting both Aurora-A and -B) Danusertib (PHA-739358), PF-03814735 and AMG 900 show anti-proliferative activity in vitro (against multiple cancer types), are orally bioavailable and are currently undergoing phase I clinical trials [31,33].

In late G2 phase, the matured centrosomes separate and migrate to opposite poles, initiating mitosis. Centrosome separation requires the regulated removal of the physical linkage tethering the duplicated centrosomes together. Rootletin, and its interacting partner, C-NAP1 (CEP250), are critical for maintaining this centrosome cohesion. The NIMA Related Kinase 2 (Nek2) phosphorylation of C-NAP1 leads to its dissociation from centrioles, and the initiation of centrosome separation [71]. High levels of Nek2 protein and Nek2 mRNA overexpression have been reported in breast cancer and in individual subtypes, making Nek2 a relevant drug target [72]. JH295 and NCL 00017509 are the latest generation of Nek2 inhibitors, exerting potent, specific and irreversible inhibition, and are currently in pre-clinical studies in vitro [39,40]. Interestingly, in addition to Nek2’s centrosomal role, there is Nek2-dependent activation of Alternative Reading Frame (ARF) protein in response to ATM depletion, which acts as a secondary protective checkpoint response in ATM-deficient cells [73]. This finding has important implications for the future clinical use of Nek2 inhibitors, particularly when used in combination therapies with ATM inhibitors (see Section 2.2 and Section 3.1.2 for ATM inhibitors).

G2–M transition is orchestrated by the key cyclin dependent kinase. In addition, Cdk1 promotes centrosome separation by phosphorylating and activating Eg5 at the spindle poles [49]. Inhibiting Cdk1 can cause cell cycle arrest at G2/M, but active Cdk2 allows cells to progress through the centrosome cycle, resulting in the production of multiple centrosomes [61]. RO-3306 and CGP 74514A are selective ATP-competitive Cdk1 inhibitors that cause cell cycle arrest, centrosomal defects and are currently undergoing further pre-clinical development [41,42,43,74,75] (Table 1). During G2-M transition, the two matured centrosomes move in opposite directions along the nuclear envelope to form the bipolar spindle. Eg5 (KIF11, kinesin-5, KSP) is a plus end mitotic kinesin that generates the sustained outward forces required for centrosome separation, movement and formation of the bipolar spindle [45]. Localisation of Eg5 to the centrosome requires intact microtubules and Plk1 [49]. Any aberration in Eg5 expression can affect cell division and leads to centrosome separation defects. HSET (KIFC1) is a minus-end interpolar mitotic kinesin and an Eg5 antagonist. While HSET is involved in microtubule organisation and stability, it plays an interesting role in the survival of cancer cells with supernumerary (≥3) centrosomes, by facilitating centrosome clustering (reviewed below in centrosome clustering Section 2.3) [76]. Monastrol was identified as the first small molecule Eg5 inhibitor (allosteric inhibitor of ATPase activity) and causes mitotic arrest and a monoastral spindle in cells [45,77]. Current next generation Eg5 inhibitors in clinical trials include the quinazolinone derivative, Ispinesib (SB-715992, CK0238273), which disturbs Eg5 microtubule movement by inhibiting ADP release, and is in phase II trials (Table 1). The allosteric Eg5 inhibitor, MK-0731 (non-competitive against ATP or microtubules) has completed a phase I trial (NCT00104364). Filanesib (ARRY-520) has completed (NCT01989325, NCT02092922) and is in a current phase I/II clinical trial against Multiple Myeloma (MM) (NCT02384083) [45,46,48]. 

### 2.2. Centrosome Amplification Inhibitors

Centrosome amplification (CA) refers to an aberrant centrosome number (≥3 per cell), which is a hallmark feature of many high-grade tumours [15]. CA is a trigger of chromosome instability (Figure 1A) and is now thought to act as a driver for cancer progression [59]. Possible mechanisms that result in CA include centrosome reduplication, cytokinesis failure (resulting in genome doubling and CA), centriole splitting and PCM fragmentation, and de novo formation of microtubule organizing centres (MTOCs) without centrioles and DNA damage [59,78]. In this section, small molecule inhibitors targeting key CA regulating proteins are discussed.

Genetic instability due to failed DNA repair mechanisms is an important pathway by which tumour cells induce CA. Cells employ DNA repair mechanisms (reviewed in detail in Section 3) to rectify severe DNA damage, like double strand breaks (DSB). Loss or inactivation of proteins involved in DNA damage repair pathways leads to persistence of DSB, which triggers centrosome amplification (reviewed in [79]). Dysregulation of key DNA damage proteins—ATM (Ataxia-telangiectasia mutated), ATR (Ataxia and Rad3-related), Chk1 (checkpoint kinase 1), Cdk1 (cyclin-dependent kinase 1), Cdk2, Rad51, Rad54, MCPH1, BRCA1 (breast cancer susceptibility gene 1) and BRCA2—has been implicated in CA induction [61,78,80,81,82]. CA in normal cells is poorly tolerated with tumour suppressor gene p53, and its downstream targets, p21 and Gadd45a, regulating the formation of supernumerary centrosomes [83]. In accordance with centrosomal activities, many DNA damage response (DDR) proteins physically localise and accumulate at the centrosome in a temporal manner throughout the cell cycle, including the apical DSB response kinases, ATM and ATR, which localise at centrosomes and regulate centrosome number during the G2 checkpoint [82]. Caffeine is a well-characterised inhibitor of both kinases [84]. Interestingly, ATM and ATR act in a complementary (or redundant) manner in driving centrosomal responses to DNA damage, as a caffeine-sensitive activity allows CA in ATM- or ATR-deficient cells [78]. Caffeine has been tested in multiple phase II/III/IV clinical trials against multiple tumour types (Table 2). Selective ATM and ATR inhibitors are discussed in the context of the DNA repair pathways in Section 3.1.2 (Table 4). Downstream of ATM/ATR in the DNA damage pathway, Chk1 is phosphorylated and localises to the centrosome which leads to the induction of CA [78,85,86]. Chk1 activation and centrosomal localisation blocks cyclin B–Cdk1 activation, negatively regulating mitotic entry and causing a prolonged G2 arrest, during which CA occurs [78]. Furthermore, Chk1 signalling causes CA after ionizing radiation (IR) by upregulating Cdk2 activity through activating phosphorylation [61]. UCN-01 (a Staurosporine derivative) is a reversible ATP-competitive Chk1 inhibitor (with dose dependent inhibition of other protein kinases) in clinical trials. MK-8776 (SCH 900776) is a potent Chk1 inhibitor which abrogates the G2 block and inhibits DSB repair in vitro, and is currently in phase II clinical trials (Table 2).

The DDR enzyme, poly(ADP-ribose) polymerase 1 (PARP-1) localizes to centrosomes and is activated following single- and double-strand DNA breaks (PARP-1 inhibition in the context of DNA repair is discussed in detail in Section 3). Centrosome hyperamplification is observed following treatment with specific PARP inhibitors: 3-AB (3-aminobenzamide), rucaparib and the quinazoline, NU1025, as well as in PARP-1-null cells [87,88,89,90]. This induction of CA by PARP inhibitors does not require DNA lesions and it is thought to be caused by a signal transduction pathway involving poly(ADP-ribosyl)ation of centrosomal proteins [91]. Interestingly, the cytotoxicity of a select group of PARP inhibitors is attributable to the de-clustering of amplified centrosomes (discussed in Section 2.3), leading to mitotic catastrophe. As such, PARP-1 inhibition is an emerging therapeutic target for centrosome-associated cancers.

Phosphoinositide 3-kinase (PI3K) is a crucial signalling pathway involved in cell proliferation, growth and survival, and a high incidence of PI3K pathway alterations is observed in many cancers. Activation of the PI3K-Akt pathway can contribute to CA, as inhibitors targeting key proteins of aberrant PI3K-Akt pathways can suppress centrosome amplification [100]. Many of the PI3K inhibitors are known to bind competitively to the ATP-binding pocket in the catalytic domain of the p110α catalytic subunit. A range of PI3K inhibitors (known to affect centrosomes) are currently enrolled in pre-clinical and clinical trials (including phase II clinical trials against a range of cancer types) (Table 2) [94,95,96]. Hyperactive Met-PI3K-Akt deregulates centrosome duplication and causes multipolar spindles and CIN, and the PI3K inhibitor, LY294002 (a reversible ATP competitor), has been reported to significantly suppress Met-induced supernumerary centrosomes [71,73]. Mutations in the PIK3CA gene, encoding the p110α catalytic subunit, have been reported in ~30% of breast cancers (mainly estrogen receptor positive; ER+), and many other cancer types (including ovarian, urological, neural and brain, lung, colorectal and pancreatic) [101,102]. Mutant PIK3CA (p110αH1047R) can cause sustained PI3K pathway activation, and induce CA at the G1/S transition via a pathway involving Akt, ROCK and Cdk2/Cyclin E-nucleophosmin [59,96,103]. The Cdk2-Cyclin E complex phosphorylates nucleophosmin, which interacts with ROCK II to initiate centrosome overduplication. These interacting CA events can be blocked by the inhibition of Cdk2, PI3K p110α or ROCK, preventing CA (Table 2). 

As previously discussed (Section 2.1), Plk4 is a master regulator of centrosome duplication, and Plk4 dysregulation is a major contributor to CA, tissue hyperplasia and chromosome instability [104,105,106,107,108]. A small molecule Plk4 inhibitor, CFI-400945 (an ATP competitive inhibitor), was tested in patient-derived mice xenografts (against a range of cancer types) and significantly reduced tumour growth and increased overall survival [30,109,110]. Interestingly, the effects of CFI-400945 were found to be dose dependent, with in vitro studies using high concentrations (≥200 nM) reducing centriole numbers, and low concentrations (10–100 nM) increasing the number of centrioles [30]. The dose dependent effects of CFI-400945 are mediated by full (high concentration) and partial (low concentration) inhibition of Plk4 activity. Partial Plk4 inhibition reduces Plk4 autophosphorylation, targeting it for degradation. However, enough activity remains for the phosphorylation of centrosome substrates and the induction of centriole overduplication [30]. Currently, orally active CFI-400945 is undergoing phase I clinical trials (NCT01954316, NCT03187288) (Table 2).

### 2.3. Centrosome Clustering Inhibitors

Cells harbouring supernumerary centrosomes should either arrest during the spindle assembly checkpoint, or produce a multipolar cell division, which leads to complete mitotic catastrophe or severe aneuploidy in the resultant progeny [59,103] (Figure 1A). However, given that CA is a key feature of high-grade tumours, tumour cells clearly employ a mechanism for survival in the face of extra centrosomes, namely, centrosome clustering (CC). Centrosome clustering gathers together supernumerary centrosomes during mitotic spindle assembly to form pseudo-bipolar cell division. The de-clustering of supernumerary centrosomes specifically eliminates cells with CA by causing multipolar mitosis and massive aneuploidy, which ultimately results in cell cycle arrest and cell death [59]. Thus, the inhibition of centrosome clustering is an exciting new therapeutic approach for cancer treatment. While the precise mechanism of centrosome clustering is not fully understood, inhibitors targeting known mechanisms are highlighted below.

The oncogenic transcription factor, STAT3 (Signal Transducer and Activator of Transcription 3), was recently shown to regulate centrosome clustering via a pathway involving Stathmin (a regulator of microtubule dynamics) and the mitotic kinase, Plk1 [111,112,113]. STAT3 is reported to be active in various cancer cell types, and it is primarily involved in the transcriptional regulation of apoptosis, inflammation, and invasiveness [114]. It has been shown that STAT3 inhibition leads to a transcription-independent mechanism that prevents centrosome clustering [113]. The inhibitor, Stattic (STAT three inhibitory compound) alters the SH2 domain of STAT3 and indirectly inhibits phosphopeptide binding, blocking the STAT3 mediated regulation of Stathmin, allowing its microtubule depolymerase function to remain active [112] (Table 2). Plk1 has been shown to induce clustering in endothelial cells by increasing γ-tubulin levels, and inhibition of STAT3 decreases downstream Plk1 phosphorylation, which reduces total γ-tubulin protein expression [115]. Napabucasin (BBI-608) is an orally-administered first-in-class STAT3 inhibitor that is currently active in >20 clinical trials (five in phase III) [111,112,113]. However, it should be noted that in addition to centrosomal activities, STAT3 inhibitors significantly inhibit cancer stem cell gene regulation. 

The anaphase-promoting complex/cyclosome (APC/C) is an essential ubiquitin-protein ligase that is involved in cell cycle progression. The activation of APC/C is dependent on its two co-activator subunits, CDC20 and CDH1. While APC/C-CDC20 principally regulates mitotic progression, APC/C-CDH1 has a broad spectrum of substrates that includes proteins involved in the regulation of centriole disengagement [116,117,118]. During mitosis, the APC/C-CDH1 complex regulates the downstream substrate, kinesin motor Eg5, which maintains balanced spindle forces and plays an essential role in centrosome clustering. Inhibition of APC/C-CDH1 increases Eg5 protein levels, resulting in a multipolar spindle phenotype specifically in CA cells [119]. Pro-TAME and Apcin are selective APC/C-CDH1 inhibitors (both disrupting protein–protein interactions) currently in pre-clinical development (Table 3). APC/C inhibitors represent a new therapeutic approach to specifically target tumour cells harbouring CA, while leaving the surrounding normal cells viable [118,120,121]. 

Microtubule motor human NCD (nonclaret disjunctional) homolog HSET (KIFC1) is a kinesin-14 family member. Kinesin-14 proteins are specific minus-end microtubule-directed motors that cross-link microtubules and coordinate spindle assembly. HSET utilizes the energy derived from ATP hydrolysis to move along microtubules from plus to minus-ends. The activity of HSET is not essential for spindle assembly in cultured cells because centrosomes can mask its function [144,145,146]. HSET is a direct binding partner of the centrosomal protein CEP215, and the CEP215–HSET complex promotes centrosome clustering, forming pseudo-bipolar spindles in cancer cells with CA [76]. Thus, targeting this complex has been shown to be specifically cytotoxic to CA cells [125]. Furthermore, recent work suggests that the loss of E-cadherin in cells increases cortical contractility, restricting centrosome movement to a minimal distance which facilitates HSET binding to the microtubules of multiple centrosomes, thus promoting clustering [144]. In addition, HSET is overexpressed in human cancer and mediates therapeutic resistance in breast cancer, making it a promising new therapeutic target [147]. CW069 is an allosteric, and selective inhibitor of HSET that increases multipolar spindles by inducing centrosome de-clustering in cells harbouring CA. Alternatively, AZ82 is a reversible and potent ATP-competitive (microtubule non-competitive) inhibitor of MT-stimulated ATPase HSET activity, which stimulates centrosome de-clustering in CA cancer cells [45,123,125] (Table 3).

Griseofulvin, an orally active antifungal drug, was identified as a centrosome de-clustering agent in a phenotype-based screen [148]. Griseofulvin induces multipolar spindles by disrupting the interphase microtubule network, via inhibition of microtubule polymerisation [142,148,149]. GF-15, a griseofulvin derivative, is a significantly more potent centrosome de-clustering agent which causes multipolar cell divisions and subsequent tumour-specific cell death [128]. GF-15 reduces spindle tension (without significantly impacting tubulin polymerisation) through inhibition of microtubule dynamic stability, leading to spindle multipolarity in CA cells [126,128,139] (Table 3).

An orally bioavailable phthalazinone PARP inhibitor, AZ0108, was identified as a potent de-clustering agent using a cell-based phenotypic screen to identify inhibitors of centrosome clustering. AZ0108 showed selective inhibition against PARPs 1, 2, 5a (tankyrase-1; TNKS1) and 6. Importantly, a siRNA screen for specific PARPs found that TNKS1 is essential for clustering and the formation of bipolar spindles [130]. PolyADP-ribosylation of TNKS1 may contribute to spindle bipolarity by providing a static matrix, anchoring microtubule-associated motor and spindle proteins [124]. A second PARP inhibitor, PJ-34, is currently undergoing pre-clinical trials and is known to induce distorted multipolar spindles and to disrupt bipolar clustering of extra centrosomes resulting in mitotic catastrophe and cell death, an effect exclusively eradicating cancer cells harbouring CA without affecting normal cell proliferation [124,132,150]. AZ0108 and PJ-34 showed better centrosome de-clustering abilities, compared to isoquinolinone-derived PARP inhibitors Tiq-A and Phen (phenanthrene) [124,151] (Table 3). They also performed better than the non-phenanthrene derivative (orally available PARP inhibitor) Veliparib (ABT-888). While Iniparib (BSI-201) was described as a PARP inhibitor (and found to have centrosomal activity), it has subsequently been reported to be a non-selective modifier of cysteine-containing proteins (making it a protein-reactive compound), rather than a bona fide PARP inhibitor [134,135] (Table 3).

Two centrosomal proteins critical for centrosome clustering are Transforming Acidic Coiled-Coil Containing Protein 3 (TACC3) and Colonic and Hepatic Tumour Overexpressed Gene (ch-TOG). Integrin-linked Kinase (ILK) regulates TACC3 phosphorylation at Ser558, in an Aurora A-dependent manner, and disruption of this phosphorylation leads to a destabilised spindle, astral microtubules and errors in microtubule attachment to centrosomes [129]. ILK depletion or inactivation can affect Aurora-A–TACC3 interactions. QLT-0267 is a pharmacological inhibitor that targets ILK kinase activity (binding to the ATP-binding site of ILK) and induces centrosome declustering [129]. QLT-0267 treatment causes a decrease in TACC3 phosphorylation, which leads to a destabilised spindle and errors in microtubule attachment. In vitro, QLT-0267 shows a significant selectivity for cancer cells with supernumerary centrosomes, compared to normal cells, indicating that ILK inhibition provides a selective way of targeting cancer cells [129,136].

A novel centrosome clustering pathway was identified, involving NIMA-related kinase 6 (Nek6) and Hsp72, mediated by the upstream regulators Aurora-A and Plk1 [137]. Nek6 is activated by Plk1, and Aurora-A which targets Hsp72 to the poles of cells with amplified centrosomes. Nek6 phosphorylates Hsp72 which modulates the interaction of clathrin with the chTOG–TACC3 complex, facilitating Hsp72 spindle association and promoting K-fiber stability. Blocking Hsp72 or Nek6 activity produces multipolar spindles in cells with supernumerary centrosomes [137,152]. VER-155008 is an ATP-derivative Hsp70 inhibitor which also blocks the catalytic activity in most of the members of the Hsp70 family (including Hsp72, Hsc70 and Grp75) and inhibits Hsp72-Nek6-mediated centrosome clustering [138]. Novel compounds targeting this centrosome clustering pathway may hold promise as an additional method for targeting CA cancer cells. 

CCCI-01 (N2-(3-pyridylmethyl)-5-nitro-2-furamide) was identified during a high-content screen for agents that block centrosome clustering in a breast cancer line harbouring high centrosome numbers. The specific target of CCCI-01 has yet to be identified, however, CCCI-01 may target a protein that is highly expressed during mitosis or has a mitosis-specific function [139]. Further investigation is needed to reveal the specific CCCI-01 target(s) (novel or known) and determine if future work with this compound is justified.

Noscapinoids represent a novel class of microtubule-modulating agents that circumvent the stronger effects of other tubulin-binding chemotherapeutics, by binding tubulin without altering its monomer/polymer ratio [153]. EM011 is a potent non-toxic noscapinoid derivative demonstrated to induce G2/M arrest, inhibiting cellular proliferation and tumor growth, in human xenograft models. EM011 modulates microtubule dynamics by inhibiting the association of microtubule plus-end tracking proteins, like End Binding Protein-1 (EB1) and cytoplasmic linker protein-170 (CLIP170) [140,149] (Table 3). Importantly, EM011 was found be a centrosome de-clustering agent, inducing both centrosome hyperamplification and the formation of multipolar spindles due to persistent centrosome declustering. EM011 induces CA via upregulation of Plk4 and Aurora-A protein levels and dysregulation of the centriole duplication cycle. This dual activity of EM011 suggests it may (potentially) emerge as a useful therapeutic. 

Originally developed for the inhibition of Platelet-derived Growth Factor Receptor b (PDGFR-b), Crenolanib and CP-673451 were found to act as robust centrosome de-clustering agents. These ATP competitive inhibitors induce mitotic spindle multipolarity by activating cofilin protein, leading to cortical actin network destabilisation [140,142]. Crenolanib shows great promise as an anticancer agent and is enrolled in phase III clinical trials. CP-673451 has been tested extensively in pre-clinical xenograft mouse models and causes significant tumour growth inhibition [143,154] (Table 3).

## 3. Small Molecule Inhibitors Targeting Chromatin 

**Figure 3:**
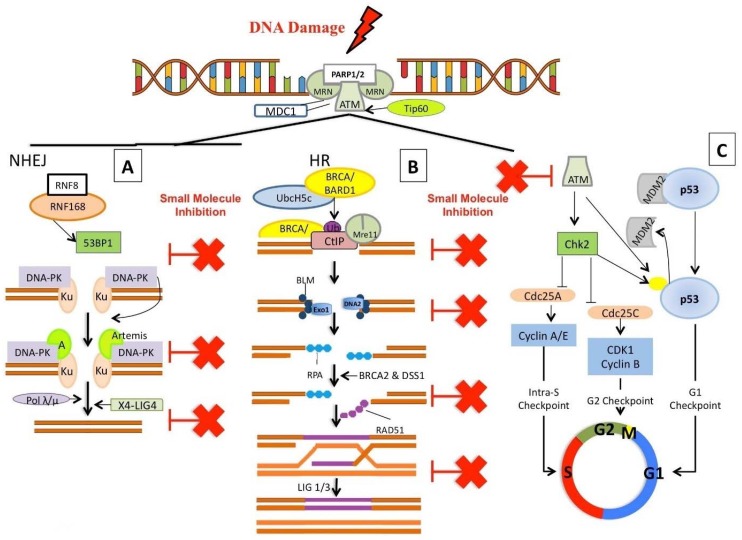
**Overview of double strand break chromatin stability mechanisms.** (**A**) Brief schematic of NHEJ (Non-Homologous End Joining) pathway; (**B**) Brief schematic of HR (Homologous Recombination) pathway; (**C**) Cell cycle related genome stability mechanisms. Key points where inhibitors can be used to disrupt the pathways are indicated.

The DNA damage response (DDR) evolved to repair damaged DNA upon initiation of cell cycle checkpoints, providing the necessary time for the damaged DNA to be repaired before allowing cells to re-enter the cell cycle [9,155,156,157]. The most damaging DNA lesion is the DNA double strand break (DSB), where failure to rejoin and repair the damaged DNA can lead to genomic instability and cancer. There are two key processes for repairing DSB, homologous recombination (HR) and non-homologous end joining (NHEJ), as previously discussed (see Introduction) [158,159]. It is established that many cancers have defects in their genome stability mechanisms, in either the HR or NHEJ pathway, ultimately leading many cancers to rely on a single pathway to maintain genomic stability [9,155,157,160]. Inhibition of key cellular processes which cancer cells rely on for survival has led to the development of targeted inhibitors, specifically tailored to/targeting the molecular profile of each tumour. Clinical use of these targeted inhibitors is at the leading edge of the latest wave of advances in precision medicine [1,161]. In the following sections, we discuss targeted inhibitors that broadly inhibit the DSB response, or specifically target either the HR or NHEJ pathway (Figure 3). The class of inhibitor, its mechanism of action, and cancer specific applications are briefly described to give context and relevance. Priority is given to inhibitors currently in clinical trials, however new inhibitor classes/novel inhibitors are described where they have interesting pre-clinical or potential future applications (particularly as first in class descriptions). 

### 3.1. Inhibitors Targeting DNA Damage Signalling and Processing 

**Figure 4:**
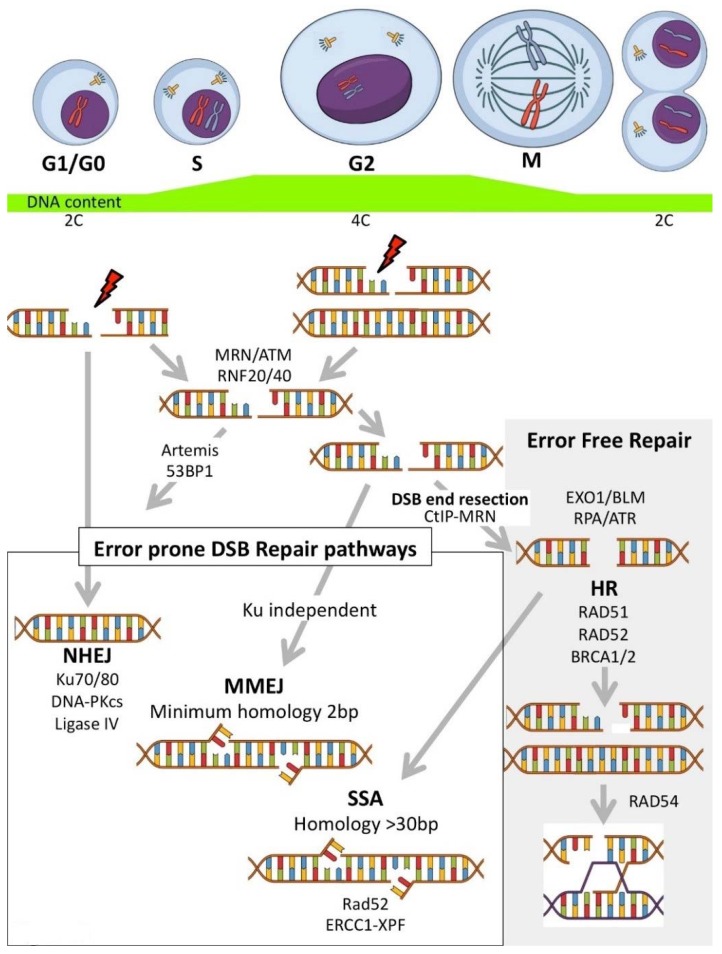
**Schematic overview of major pathways used to repair DSB.** Certain key proteins are indicated. NHEJ: Non-homologous End Joining; HR: Homologous Recombination; MMEJ: Microhomology-Mediated End Joining; SSA: Single Strand Annealing.

In addition to the major classical cell cycle dependent repair pathways (NHEJ and HR) (Figure 3A–C), there are at least two other error-prone pathways involved in repairing DSBs: Microhomology-Mediated End Joining (MMEJ) and Single Strand Annealing (SSA) (Figure 4). MMEJ is a Ku independent end-joining pathway mediated by the alignment of microhomologous sequences (>2 bp reported) internal to the broken ends that facilitate end joining. MMEJ results in deletions and insertions at the original break site and can led to chromosome translocations. SSA arises when a DSB occurs between homologous repeats. SSA is mediated by RAD51 and CtiP-MRN allowing DSB end resection, forming 3′ ssDNA. This exposes the homologous sequences flanking the DSB (>30 bp) which can then annealed together (reviewed in [162,163,164,165,166,167]) (Figure 4). The following text discusses current and potential targeted cancer therapeutics (and their mechanisms of action) using compounds that inhibit key proteins with roles integral to DSB repair (general DSB recognition and/or initiation of repair) (Table 5) and then compounds specifically targeting the NHEJ, MMEJ, SSA or HR pathways.

#### 3.1.1. PARP Inhibitors

While the role of poly-(ADP-ribose)’s (PAR) is established in the repair of single-strand DNA breaks (SSB), recent work has demonstrated the role of PARs in the detection and repair of DSBs [156,158,168,169]. PARP1 is an abundant nuclear protein that catalyzes the polymerisation of ADP-ribose units, resulting in the attachment of PAR polymers to PARP1 or other target proteins. PARP1 poly(ADP)ribosylation (PARylation) activity is one of the earliest steps of DNA damage recognition and is essential for initiating various forms of DNA repair (for review see [160]). PARP1 substrates, like the key DSB protein, ATM (discussed below Section 3.1.2), contain PAR-binding domains, and interactions with PARP1 stimulate their activity [170,171]. PARP1 is frequently upregulated in many cancers; therefore, blocking its activity using small molecules has great therapeutic potential [156,172,173,174]. PARP1 is involved in the early recruitment of factors to facilitate DSB repair, and its inhibition results in delayed activation of key DDR proteins, such as H2AX, p53 or SMC1 (Structural Maintenance of Chromosomes protein 1) [160,171,172]. Additionally, PARP inhibitors may show promise as dual action compounds (previously discussed in Section 2.2).

In cells with intact HR, DSBs that occur as a result of PARP1 inhibition can be resolved, but in tumour cells lacking HR, PARP1 inhibition leads to persistent DSBs and cell death [172,175]. Cells with Breast Cancer Susceptibility gene 1 or 2 (BRCA1 or BRCA2; E3 ubiquitin-protein ligases and essential components of the HR pathway) defects exhibit high sensitivity to PARP1 inhibitors, producing high levels of DNA damage, cell-cycle arrest and cell death. Partly, this is due to PARP inhibitor (PARPi) stimulation of error-prone NHEJ in the HR-deficient cells, leading to genomic instability and cell death [172,173,174,176,177]. PARPis have a common mechanism of action, trapping PARP1 at the site of DNA damage, preventing autoPARylation and release of PARP1 [156,175,177]. PARPis bind within the nicotinamide-binding pocket in the ADP-ribosyl transferase catalytic site, making contact with the regulatory subdomains [156,177,178]. 

The synthetic lethality conferred by PARPis, especially as a targeted treatment in breast and ovarian cancers, has been tested in clinical trials since 2003. Currently, there are three FDA (US Food and Drug Administration) approved PARPis [olaparib (Lynparza), rucaparib (Rubraca), and niraparib (Zejula)], with at least eight new PARPis in clinical trials, as either a monotherapy or in combination with other treatments (Table 2 and Table 4; >180 PARPi cancer trials combined, 19 PARPis in phase III cancer trials against multiple cancers). The approved PARPi are indicated for use in adult patients with germline and/or somatic BRCA-mutated ovarian cancer, and recently, olaparib has been approved for treatment of metastatic BRCA-mutated breast cancer. CEP-9722 (a prodrug of CEP-8983) is a selective PARP-1/PARP-2 inhibitor that is anticipated to reduce the myelosuppression observed with other oral PARP inhibitors [179]. PARPis have different PARP1 trapping efficiencies, with talazoparib having the greatest and veliparib the least [176,177,180]. Additionally, there are reports of off target effects, such as with rucaparib (additionally see Table 2), which have been proposed to add to their effectiveness [156]. Multiple mechanisms of resistance to PARPi have been identified, especially in ovarian cancer patients with BRCA-deficient tumours, where many tumours develop PARPi resistance within the first year [175,180]. It has been shown that BRCA deficient tumours acquire PARPi resistance by deletion of a mutation in BRCA1 or BRCA2, restoring DNA repair by HR [156,178]. Another mechanism of PARPi resistance includes inactivation of 53BP1, which prevents end resection, a crucial step for the initiation of HR [175,181]. BRCA hypomorphs (cancer causing) with residual activity of mutant proteins are able to partially compensate for the absence of the wild-type proteins following PARPi treatment [156]. Overexpression of RAD51, a key protein in DSB repair by HR, has been linked with therapy resistance to PARPis in triple-negative breast cancer cells. Combining inhibitors that target these resistance mechanisms with PARPi may avoid resistance or resensitize cells to PARPis [181,182]. Combinational therapies targeting these resistance mechanisms (using inhibitors described below) are showing promise in overcoming acquired PARPi resistance, improving treatment efficacies.

#### 3.1.2. ATM and ATR Kinase Inhibitors

Cells respond to SSBs and DSBs through two linked molecular signalling pathways, regulated by the apical kinases ATR (Ataxia and Rad3-related) and ATM (Ataxia-telangiectasia mutated) respectively. ATM and ATR belong to the PIKK family (phosphatidylinositol 3-kinase-related kinase) of serine/threonine protein kinases, which includes DNA-PKcs (DNA-dependent protein kinase catalytic subunit) [190]. Both ATM and ATR are activated after DNA damage and function as apex transducers of DNA damage response signalling, inducing signalling cascades through the phosphorylation of numerous targets, such as H2AX, p53 and the checkpoint kinases, Chk1 and Chk2 [191]. Distinct DDR are coordinated by individual PIKK kinase-mediated signalling cascades, either the ATM–Chk2 (DSB) or ATR–Chk1 (SSB) pathways. Current work suggests that while these pathways primarily act in parallel, they do have overlapping functions and a more complex relationship [182,192]. Proper activation of ATM (or ATR) is essential for the correct coordination of cell cycle checkpoints and DNA repair processes, ultimately modulating key biological consequences, such as apoptosis or senescence. In vitro, it has been shown that inhibition of ATM or ATR can sensitize cancer cells to genotoxic agents, highlighting their potential as therapeutic targets [188]. However, the high level of sequence similarity between the kinases catalytic domains has represented a challenge for the development of specific inhibitors. 

ATM inhibition represents an exciting clinical opportunity to hypersensitise tumours to chemo/radiotherapy. Most ATM inhibitors demonstrating specificity for ATM share a mechanism of action, binding to the ATP binding pocket of ATM and blocking its kinase activity (Table 4; additionally, Table 2). A widely used ATM inhibitor (for research) is KU-55933, which was the first cell-permeable, potent and selective ATM inhibitor described [170]. KU-55933 is an ATP-competitive inhibitor of ATM and exposure of cells to KU-55933 induces a significant sensitisation to DSB-inducing agents [170,193]. However, as lipophilicity issues complicate its clinical utility, it has not progressed into clinical trials. KU-60019 (a KU-55933 analogue) is an ATP-competitive ATM inhibitor designed for improved efficiency and exhibits better solubility; however, its bioavailability remains poor [184,188]. It has been demonstrated that, in addition to inhibiting the DDR, KU-60019 reduces pro-survival signalling, migration and invasion of tumour cells and radiosensitises cancer cells [184,185,194]. KU-60019 suppresses the proliferation of breast cancer cells and sensitised cells to doxorubicin, making it a promising combinational therapeutic for non-invasive breast cancer [186,193]. KU-59403 (structurally related to KU-60019) has been developed with improved solubility, bioavailability and selectivity for ATM and holds more clinical promise [195]. CP466722 is a selective non-toxic ATM inhibitor, able to rapidly and importantly, reversibly inhibit ATM activity. CP466722 was discovered though compound library screening [185,196]. Recently a novel class of ATM inhibiting compounds was developed, 3-quinole carboxamides, including AZD0156 which binds to and inhibits ATM kinase activity [11,197]. AZD0156 is reported to be highly soluble, showing robust efficacy in mouse xenograft models in combination with DSB-inducing agents [194]. In mice models, AZD0156 has demonstrated its potential as a therapeutic for the treatment of acute myeloid leukaemia [197]. AZD0156 is a first-in-class orally available ATM inhibitor and is the only ATM inhibitor enrolled in clinical trials (phase I, in combination with olaparib for locally advanced/metastatic cancer, NCT02588105) [195] (Table 4). 

The first reported ATR-selective small-molecule inhibitor, Schisandrin B (SchB), abrogated ATR kinase activity and therefore, ATR-mediated UV-induced intra-S-phase and G2/M cell cycle checkpoints, sensitising cancer cells to UV radiation. However, high SchB concentrations were needed for this inhibition [198,199]. A more potent ATR inhibitor, VE-822 (VX970), was recently developed and radiosensitised cancer cells in vitro and in vivo [187]. VE-822 inhibited ATR kinase activity and dramatically enhanced the efficacy of cisplatin in xenograft models [198]. VE-822 was the first ATR inhibitor to enter clinical trials and is currently in phase I/II trials (in combination with topotecan in small-cell lung cancer; NCT02487095) and in multiple trials for the treatment of advanced refractory solid tumours (alone and as a combinational therapeutic) (Table 4). AZD6738 is an ATP competitive ATR inhibitor (an analogue of the previously identified ATR inhibitor, AZ20) with significantly improved solubility, bioavailability and pharmacokinetic properties (compared to AZ20) and the ability to combine with multiple agents [188,200]. AZD6738 is currently enrolled in 10 phase I/II trials against multiple cancer types (including CLL, breast cancer, high-grade carcinomas, NSCLC) [201,202,203]. BAY-1895344 is a potent, orally available and selective ATR (kinase activity) inhibitor that in vivo exhibited strong antitumour efficacy as a monotherapy in a variety of DNA damage deficient preclinical xenograft tumour models (ovarian, colorectal prostate and cell lymphoma models) [189]. Currently, BAY-1895344 is in a phase I trial (NCT03188965) as a monotherapy treatment for advanced solid tumours and lymphomas (Table 4). NU6027 (6-cyclohexylmethoxy-5-nitroso-2,4-diaminopyrimidine) was originally demonstrated to inhibit Cdk2 and additionally, was found to inhibit ATR kinase activity. In vitro, NU6027 increased sensitivity to DSB agents and PARPi, and is a promising new dual agent ATR/Cdk inhibitor [204].

#### 3.1.3. DNA Helicase Inhibitors

DNA helicases play a key role in maintaining genomic stability, functioning in DNA replication and the DDR. Expression of many DNA helicases is upregulated in tumours, enhancing proliferation or resistance to DNA damaging chemotherapeutics. In contrast, downregulation of DNA helicases leads to chromosomal instability, promoting carcinogenesis [205]. The RecQ family of DNA helicases has five members: RecQL1, BLM, WRN and RecQL4 and RecQL5 [206]. Members have key roles in maintaining genomic stability, with inactivation leading to cancer predisposition syndromes including Bloom’s syndrome (BLM) [207,208], Werner syndrome (WRN) [209], and in the case of RecQL4, three syndromes: Rothmund–Thomson syndrome (RTS), Baller–Gerold syndrome (BGS) and RAPADILINO [210,211]. RecQL1 is a DNA repair protein whose activity is regulated by PARP1, and plays a key role in the recovery from replication stress induced by topoisomerase I inhibitors [201]. While no (known) syndromes are associated with RecQL1 or RecQL5 mutations, they play important roles in genome stability [212,213,214]. 

BLM is the key element in a complex that includes topoisomerase IIIα and the RecQ-mediated genome instability (RMI) sub-complexes (RMI1 and RMI2), and its coordinated action is critical for unwinding a wide range of DNA structures that can arise during DNA replication and repair [215]. BLM has multiple roles in the HR-mediated DNA repair pathway, including DNA end resection and RAD51 filament and D-loop formation [216,217]. The BLM inhibitor, ML216, targets BLM helicase activity, either by competing for ATP binding or by preventing BLM from binding to DNA, and exhibits selectivity (over related helicases) [156,218]. Additionally, BLM inhibitors show promise as agents, targeting the 5–10% of tumours that depend on the alternative lengthening of telomeres pathways for survival [218]. 

WRN plays a prominent role in replication fork progression following DNA damage or replication fork arrest. While WRN inhibitors have been created, none have progressed into clinical trials. NSC 617145 inhibits WRN helicase activity, but not its nuclease activity, and its likely mechanism of action is by trapping WRN on the DNA substrate. This results in the accumulation of stalled replication forks, impaired growth, and the sensitisation of cancer cells to DNA-damaging agents. Inhibition of WRN activity by NSC 617145 in Fanconi Anemia (FANCA and FANCD2) or DNA-PKcs deficient cells sensitised them to mitomycin C (MMC) [219]. The WRN specific inhibitor, NSC 19630, sensitised cells to topotecan, and has shown promise against leukaemia cells [220,221]. Targeted inhibition of WRN has potential as an anticancer strategy for inducing synthetic lethality, when used in combination with other pathway specific inhibitors (such as inhibitors affecting the NHEJ pathway). 

#### 3.1.4. Topoisomerase Inhibitors 

Topoisomerases are essential enzymes for transcription, DNA replication and DNA repair, controlling DNA supercoiling and entanglements. The opening of the DNA helix and the separation into two single DNA strands during transcription and replication generates supercoiling structures. Positive supercoiling can prevent further strand separation and stall polymerases, whereas negative supercoiling can extend DNA strand separation and induce the formation of irregular chromatin structures and stall RNA polymerases [222]. The role of topoisomerases is indispensable in preventing the formation of these supercoiling structures, and topoisomerases are divided into two classes according to their mechanism of action [223]. Topoisomerases I (TOP1) cleave one strand of DNA, and topoisomerases II (TOP2) cleave both strands. Through a covalent bond to a nicked DNA molecule, TOP1 forms a “cleavable complex” and catalyses two transesterification reactions, single-strand DNA cleavage and re-ligation, crucial steps for the DNA relaxation needed for transcription or chromatin replication. Targeted inhibition leads to the accumulation of TOP1 cleavage complexes, DNA damage induced genomic instability and ultimately, apoptosis or senescence. 

The TOP1 inhibitor, camptothecin has been studied for >60 years, and thousands of analogues have been developed. Camptothecin works by trapping TOP1–DNA cleavage complexes (for review see [224,225]). Despite this, only two camptothecin analogues, irinotecan (CPT-11) and topotecan, have been FDA approved for the treatment of cancer (colorectal, pancreatic cancer, ovarian, cervix, primary brain malignances and sarcomas) (Table 5) [223,226]. Irinotecan and topotecan act by inhibiting the re-ligation reaction of TOP1, which is lethal during ongoing DNA replication or transcription [227]. Irinotecan and topotecan have limitations, including dose-limiting toxicities, rapid inactivation by E-ring opening, or through targeting by efflux pumps [such as breast cancer resistance protein (BCRP) or multidrug resistance gene 1 (MDR1)] [228,229,230]. Other TOP1 inhibitors in the indenoisoquinoline family, indotecan (LMP400) and indimitecan (LMP776), have overcome the E-ring instability of camptothecins and are in an early phase clinical trial (NCT01051635) (Table 5). Indenoisoquinolines are synthetic and chemically stable, target additional genomics locations, have prolonged drug action and can overcome multidrug resistance drug efflux pumps [231,232]. Finally, a novel non-camptothecin TOP1 inhibitor (Genz-644282) was developed using a structure−activity relationship-based approach. A clinical trial of Genz-644282 is underway against solid tumours (NCT00942799), due to its favourable cytotoxic profile in bone marrow [228] (Table 5).

TOP2 enzymes are a well explored target for anticancer agents, with TOP2 inhibitors currently used for the treatment of many cancers (including breast, lung, prostate, sarcomas, haematological malignancies). There are two main types of TOP2 inhibitors, TOP2 poisons (that stabilise the cleavable complex), and catalytic TOP2 inhibitors (that interfere with TOP2 during different stages of its catalytic cycle) [236]. While TOP2 poisons activate the DDR (phosphorylation of ATM and activation of downstream targets in both HR and NHEJ pathways), patients develop resistance to those drugs [236]. However, recent work using genome-wide studies have discovered genes that may predict resistance to TOP2 poisons, improving their potential as precision medicine therapeutics [237]. Currently, TOP2 poisons are among the most frequently used clinical TOP2 inhibitors [including doxorubicin, etoposide, mitoxantrone (Navantrone^®^)] (Table 5). Doxorubicin, which is an anthracycline drug, was the first FDA approved agent targeting TOP2 and is currently used for the treatment of many cancers, including ALL (Acute lymphoblastic leukaemia) and AML (acute myeloid leukaemia), Wilms’ tumour, neuroblastoma, breast, ovarian, thyroid, gastric, Hodgkin’s disease, malignant lymphoma and bronchogenic carcinoma [233,238]. The FDA approved (1999) anthracycline, epirubicin (an active isomer of doxorubicin) is used against breast, esophageal and gastric cancers, and has fewer side effects than doxorubicin [239,240,241]. 

The TOP2 poison, etoposide, is widely used in oncology (FDA approved 1983), often in combination with other chemotherapeutics, for the treatment of many cancer types (including ovarian, testicular, small cell lung, leukaemia and lymphoma) [242]. Etoposide is an attractive TOP2 poison due to its low affinity toward free DNA, its poor DNA intercalating activity, its high selectivity to the TOP2–DNA cleavage complex and its high frequency of trapping cleavage complexes [223,243,244]. The anthracenedione, mitoxantrone is another important TOP2 poison (FDA approved 1996) used to treat multiple cancers (including prostate, breast, AML and non-Hodgkin’s lymphoma). Mitoxantrone targets TOP2 and is a potent DNA intercalator, but compared to doxorubicin, it induces less cardiotoxicity [245]. Aclarubicin is an anthracycline-based chemotherapeutic with less cardiotoxicity than doxorubicin or daunorubicin and is a second line treatment for ANLL (acute nonlymphocytic leukaemia). Aclarubicin disrupts chromatin (by TOP2 inhibition resulting in histone eviction though intercalation) and can be used to treat haematologic cancers and solid tumors and (currently enrolled in four trials against AML). Interestingly, catalytic TOP2 inhibitors, such as the complex-forming bisdioxopiperazine ICRF-187 (dexrazoxane), can be used to modulate the toxicity of TOP2 poisons [246]. Dexrazoxane is currently in Phase III–IV clinical trials against multiple tumour types, often as part of a combinational approach (Table 5). 

#### 3.1.5. Mre11 Inhibitors

Mre11, as a key component of the MRN (Mre11, Rad50, NBS1) complex, has a central role in the DDR (DSB sensing, signalling and repair) [247]. During DSB repair, exo- and endo-nuclease activities of Mre11 are crucial for DSB repair (by either the HR or NHEJ pathways) [217,247,248]. Recently, specific Mre11 inhibitors (exo- or endonuclease) were used to discover that endonuclease inhibition promotes NHEJ, while exonuclease inhibition confers a HR repair defect, defining distinct nuclease-dependent roles of Mre11 in DSB repair [234]. 

A forward chemical genetic screen identified 6-(4-hydroxyphenyl)-2-thioxo-2,3-dihydro-4(1*H*)-pyrimidinone (mirin) as an inhibitor of Mre11-associated exonuclease activity. Mirin was shown to prevent MRN-dependent activation of ATM, without affecting ATM protein kinase activity. Mirin binds in the active site of Mre11, blocking DNA phosphate backbone rotation, inhibiting its exonuclease activity and MRN-mediated ATM activation [249] (Table 5). In vitro treatment with mirin leads to HR failure, and recently, was reported to downregulate NHEJ’s repair efficiency. As mirin affects both DDR pathways, it is a poor candidate for clinical trials [249,250]. PFM39, PFM01 and PFM03 (mirin analogues) demonstrate selectivity toward Mre11 exo- or endonuclease activity (Table 5). PFM39, like mirin inhibits the exonuclease activity of Mre11, and prevents end resection. PFM01 inhibits Mre11 endonuclease activity, affecting NHEJ [234]. PFM39 causes a G2 repair defect in HR deficient cells, while PFM01 and PFM03 do not induce DDR defects, but enhance NHEJ (while reducing the HR pathway). This highlights the different phenotypes of exo- or endonuclease activity inhibition, a key issue for precision medicine, where the selection of therapeutic drugs requires profiling to determine optimal drug choice based on a tumour’s DDR pathway profile. 

#### 3.1.6. ERCC1–XPF Inhibitors

The Excision Repair Cross-Complementation Group 1 (ERCC1)-Excision Repair Cross-Complementation Group 4 (XPF), commonly referred to as ERCC1–XPF, is a heterodimer with 5′-3′ structure-specific endonuclease activity, where the XPF molecule delivers the endonuclease activity, with important roles in DSB repair. Both ERCC1 and XPF can bind to DNA and have protein–protein interactions, with ERCC1 mediating these activities. The inhibitor, F06 (NSC 130813), was discovered, by in silico screening, and found to disrupt the ERCC1–XPF interaction [251]. F06 was able to sensitize cancer cell lines to interstrand crosslinking chemotherapeutics; however, reports now suggest this activity is not specific to ERCC1–XPF, and may disrupt the ERCC1–XPA interaction [235]. Subsequent screening identified two inhibitors against ERCC1–XPF, E-X AS5-4 targeting the interaction domain for heterodimerisation and E-X AS7 targeting the XPF active site itself [235] (Table 5). Both E-X AS5-4 and E-X AS7 induced sensitivity in nucleotide excision repair deficient cells, suggesting that with further development to improve potency, they may have the potential for clinical applications. 

### 3.2. NHEJ Inhibitors

Drugs targeting specific DSB repair pathways are attractive as they can be used to sensitize cancer cells to specific DNA damaging agents [including chemotherapeutics or ionising radiation (IR)], or, in tandem with molecular profiling, can be used to target cancers with deficiencies in specific DDR pathways [252,253]. The redundant roles of many proteins in the DNA repair process makes it essential to identify and understand the predominant/alternative DNA repair mechanisms/pathways used in cancer cells. The error-prone (mutagenic) NHEJ can operate throughout all cell cycle phases, and can be suppressed by HR at S/G2 phases, depending on the chromatin background and other factors. NHEJ inhibitors can be used to target specific DDR components (such as DNA-PKcs, the heterodimeric Ku or the Ligase IV/XRCC4 complex) (Figure 3A and Figure 4) (Table 6). 

#### 3.2.1. DNA-PK Inhibitors

DNA-dependent protein kinase (DNA-PK) is a serine/threonine protein kinase (a member of the PIKK family with ATM and ATR), formed by the large catalytic subunit (DNA-PKcs) and the smaller Ku70/80 heterodimer. Following DSB recognition, the Ku complex (Ku70 and Ku80) recruits DNA-PKcs, forming the heterodimeric DNA-PK complex with Ku at the DNA terminus. DNA-PK is a central component of the NHEJ pathway and is required for the efficient repair of DSBs (for review see [254]). DNA-PK and ATM have overlapping roles in phosphorylating H2AX and initiating the DDR [190,255]. As DNA-PKcs inhibition leads to increased levels of HR, this is a strategy to sensitise cells to DSB inducing agents [256,257]. Many inhibitors targeting DNA-PKcs are designed to target its ATP binding site (in the kinase domain) [258]. Wortmannin and LY294002, non-specific ATP-competitive PIKK inhibitors inhibiting kinase activity, were early DNA-PKcs inhibitors identified and proven to inhibit DNA-PK activity and are effective radiosensitizers [259,260]. However, LY294002 and Wortmannin display a lack of specificity (concentration dependent targeting of the PIKK family), which, coupled with poor solubility or in vivo toxicity, has limited their clinical application [261]. Screening of compound libraries identified a small group of specific DNA-PKcs inhibitory molecules, including NU7026 (2-(morpholin-4-yl)-benzo[h]chomen-4-one) and NU7441 [262,263,264] (Table 6). Subsequently, NU7026 and NU7441 were found to be LY294002 analogues with improved selectivity [262]. NU7026 combines well with TOP2 inhibitors (such as doxorubicin, etoposide or mitoxantrone), but has no effect on the cell cycle alone [264]. NU7441 increased the cytotoxicity of doxorubicin and IR in vitro, but has performed poorly in pre-clinical studies [263,265]. Currently, two DNA-PK inhibitors are enrolled in clinical trials, VX-984 (also known as M9831; NCT02644278) and MSC-2490484A (also known as M3814; NCT02516813) (Table 6). VX-984 and M3814 inhibit DNA-PKcs autophosphorylation and are orally available, with potential antineoplastic and chemo/radiosensitising activities [195,266,267,268,269]. Both are in phase I trials, as a monotherapy or in combination with pegylated liposomal doxorubicin in advanced solid tumours [likely to have alterations in DNA repair mechanisms, (i.e. BRCA or ATM mutations)] or chronic lymphocytic leukaemia. Interestingly, recent work supports the idea that specific phosphorylation events on DNA-PKcs can promote HR (inhibiting NHEJ), providing additional avenues to explore the DNA-PK dependent manipulation of DSB repair pathway choice [270].

#### 3.2.2. Ligase IV Inhibitors

The DNA-end processing activities of the NHEJ pathway rely on a number of enzymes, such as Artemis and ligase IV, to generate and join the repaired DNA ends. This ligation step in NHEJ is an attractive target for inhibition, and DNA ligase IV has an essential role. Ligase IV is an endonuclease phosphorylated by DNA-PKcs, and ligase IV is the enzyme responsible for ligating repaired DNA ends [272]. Patients with ligase IV mutations or lower levels of ligase IV are radiosensitive, as inhibition/mutation of ligase IV leads to the accumulation of numerous DSBs [273]. Ligase IV silencing results in increased cellular sensitivity to the chemotherapeutic, temozolomide (a methylating agent) [274]. Therefore, ligase IV is a promising target for new cancer therapeutics. L189 was originally developed to target ligase IV and is a competitive inhibitor that blocks its DNA binding activity. However, it displays poor specificity, with equal inhibitory activity against ligases I, III and IV [253]. Recently, the L189 derivative, SCR7, was identified and proposed as a ligase IV selective inhibitor [271]. However, recent work has shown that SCR7 is neither selective, nor a strong inhibitor of DNA ligase IV, and actually exhibits greater activity against DNA ligases I and III [275]. Currently, ligase IV inhibitors are confined to the pre-clinical testing and validation stages (Table 6). 

### 3.3. HR Inhibitors

HR is the other major DSB repair pathway, which most importantly is considered to be error-free, relying on an undamaged homologous DNA template as a guide to allow the damaged DNA to be replaced precisely with the correct sequence [276,277] (Figure 3B and Figure 4). Many chemotherapeutics induce replicative stress (i.e., alkylating agents or platinum compounds which produce intra- and interstrand DNA crosslinks) which arrest replication fork progression [278,279]. In this case, HR mediated repair is critical for re-establishing replication forks and restoring cell cycle progression. As such, HR-mediated repair can alleviate those deleterious effects in cancer cells, lowering the effectiveness of these chemotherapeutic drugs, making the HR pathway an attractive target for drug development (Table 7). 

#### 3.3.1. RAD51 Inhibitors

To target the HR pathway, several studies have focused on inhibition of the key protein, RAD51. RAD51 replaces replication protein A (RPA) bound to ssDNA, forming a RAD51–ssDNA filament, facilitating a homology driven search allowing a heteroduplex DNA to form between the damaged and the intact DNA strands (DNA strand invasion and exchange). Importantly, it has been shown that stalled replication forks are restarted using two distinct RAD51-dependent processes [290]. Additionally, it has been shown that RAD51 is overexpressed in a number of cancers (including sarcomas, breast, non-small cell lung, bladder, prostate and pancreatic) [291,292,293]. It has been proposed that this upregulation of RAD51 may be a result of the high proliferative index of tumour cells, and it has been shown that lowering RAD51 expression or activity (through inhibition) can sensitise cancer cells to chemotherapeutics (such as cisplatin, doxorubicin or IR) [253]. As RAD51 plays a key role in repair, RAD51 inhibition allows specific targeting of cells committed to the HR mediated repair pathway. To date, two classes of RAD51 inhibitors exist: compounds interfering with RAD51 ssDNA binding ability, and compounds that stimulate the formation of toxic RAD51 complexes (inducing nucleoprotein filament formation). Currently, neither class has proceeded to clinical trials; however, they have demonstrated potential in pre-clinical studies, with further improvement of their solubility, toxicity and effectiveness required for progression into clinical trials (Table 7).

B02 was identified by high-throughput screening and is a specific RAD51 inhibitor, interfering with RAD51s ssDNA binding activity [294]. B02 treatment increases sensitivity to DNA-damaging agents (including cisplatin, MMC, PARP1 inhibitors) by inhibiting RAD51-dependent DSB repair [280]. In a pre-clinical mouse xenograft model, B02 improved the effects of cisplatin treatment [295]. In multiple myeloma cells (with increased rates of HR mediating chemotherapy resistance) B02 sensitises cells to low-toxicity doses of doxorubicin, resulting in significant synthetic lethality [296]. High-throughput screening identified RI-1, which binds covalently to the surface of RAD51 protein and is thought to destabilize RAD51 oligomerization/filament formation on DNA. RI-1 inhibits RAD51 foci formation following DNA damage but does not affect replication protein A (RPA) foci formation. RI-1, stably and irreversibly inhibits RAD51, sensitising cancer cells to MMC [281]. Inhibitors stimulating the formation of toxic RAD51 complexes exploit the upregulation of RAD51 (by enhancing filament stability) targeting cancer cells constitutively overexpressing RAD51 and sparing non-tumour cells. A library screen identified the RAD51 inhibitor, RS-1 (RAD51-stimulatory compound 1), which enhanced nucleoprotein filament stability [282]. RS-1 stimulates RAD51 DNA binding and recombination activities (locking it into its active conformation), and RS-1 induces synthetic lethality in RAD51 overexpressing cancer cells [297] (Table 7). Additional RAD51 inhibitors have been developed that target key RAD51–BRCA2 binding sites, facilitating DNA repair. Breast cancer susceptibility gene 2 (BRCA2) is key protein that mediates HR, and defects in BRCA2 predispose cells to DNA damage and patients to cancers (particularly breast and ovarian cancers) [298]. BRCA2 contains eight conserved BRC repeats (the primary sites used by BRCA2 to bind RAD51), which facilitate the BRCA2-mediated assembly of RAD51 nucleoprotein filaments [299]. BRCA2 BRC repeats 1–4 bind free RAD51 with high affinity and facilitate RAD51 loading onto RPA-coated ssDNA, while BRC repeats 5–8 bind the RAD51 nucleoprotein filament with high affinity (having a low affinity free RAD51) [300]. In vitro, the RAD51 inhibitor, IBR2, sensitises cancer cells to IR, by disrupting the RAD51–BRC interaction (inducing proteasome-mediated RAD51 degradation) [301]. The development of IBR120 (a IBR2 analogue) led to higher specificity and growth inhibition in a range of cancer cells, in vitro [283] (Table 7). 

#### 3.3.2. RAD52 Inhibitors

The DNA repair protein, RAD52, is involved in all HR pathways, binding ssDNA and inducing DNA annealing though interactions with RAD51 and facilitating DNA strand-exchange. Interestingly, it was recently discovered that RAD52 (in yeast) has a role in RNA-template mediated repair of DSBs, although the mechanism of action is uncertain [302]. Importantly, RAD52 depletion is synthetically lethal in BRCA2 and BRCA1/PALB2 deficient cells [303,304]. As such, RAD52 inhibitors are promising therapeutics, particularly in breast and ovarian cancers. Recent initiatives to discover RAD52 inhibitors have yielded a number of compounds that either inhibit RAD52 oligomerization or block RAD52-mediated ssDNA binding activities. A high-throughput screen of small molecule libraries discovered 6-hydroxy-d-l-dopa, which inhibited the oligomerisation activity of RAD52 [287]. Additional library screens identified D-103 and D-G23, which displayed the ability to block RAD52-mediated ssDNA annealing [284]. In another approach, molecular docking of chemical libraries led to the discovery of a number of RAD52 inhibitors, of which AICAR [5-amino-1-((2*R*,3*R*,4*S*,5*R*)-3,4-dihydroxy-5-(hydroxymethyl)-tetrahydrofuran-2-yl)-1*H*-imidazole-4-carboxamide] disrupted the RAD52-ssDNA interactions and displayed synthetic lethality in BRCA1 and BRCA2 mutated cells [285] (Table 7). Recent high-throughput screens identified a number of compounds predicted to bind within the ssDNA-binding groove of the RAD52 oligomeric ring, disrupting RAD52-ssDNA interactions. Additional in silico screening of these targets led to the discovery of the novel RAD52 inhibitor, NP-00425, which fits into the RAD52 ssDNA binding groove. Two additional compounds identified by computational modeling [(−)-Epigallocatechin) and Epigallocatechin-3-monogallate] inhibited RAD52-ssDNA binding, and were synthetically lethal in BRCA2 or MUS81 deficient cells [286] (Table 7). Together, these results demonstrate the exciting therapeutic potential of targeting RAD52-mediated DNA repair, particularly in BRCA-deficient cancers. A recent report identified 6-hydroxy-dopa (6-OH-dopa) as an allosteric inhibitor of the RAD52 ssDNA-binding domain. 6-OH-dopa is reported to suppress RAD52 recruitment and recombination activity in vitro (by disrupting RAD52 heptamer and undecamer ring superstructures), selectively inhibiting the proliferation of BRCA deficient cancer cells. As such 6-OH-dopa is a promising new potential therapeutic [305] (Table 7).

#### 3.3.3. RAD54 Inhibitors

RAD54 is a helicase and is a member of helicase Superfamily 2 of ATPase-dependent DNA translocases. RAD54 belongs to the SNF2 (SWI2/SNF2) protein family of dsDNA-dependent ATPases, but lacks DNA strand-separation activity. RAD54 has important roles in protecting genome stability in the HR pathway (in conjunction with RAD51), mediating DNA supercoiling as it translocates along dsDNA [306]. The RAD54 inhibitor, streptonigrin (STN) is an antitumor antibiotic found to bind the RAD54 ATPase domain and inactivate RAD54 by generating reactive oxygen species [288]. Streptonigrin has been used clinically for the treatment for cancer (including breast, lung, lymphoma, melanoma). However, clinically, streptonigrin induces prolonged, severe bone marrow depression, limiting its use [307] (Table 7). Recently, streptonigrin has been reported to bind to and inhibit SUMO-specific protease, SENP1, which makes its mechanism of action less clear but may help explain its clinical effects [289].

### 3.4. Targeting Chromatin Remodelling

Nucleosomes are the basic unit of chromatin, and each core nucleosome consists of an octameric complex of the core histone proteins, heterodimers of H2A/H2B and H3/H4 that wrap 145–147 bp of DNA, and are connected by linker DNA to the adjacent nucleosome [308,309]. Nucleosomes package and compact DNA, allowing higher-level structure to form in three-dimensional nuclear space (forming ordered loops, domains and chromatin fibers), ultimately assembling chromosomes [310,311]. These higher order structures mediate essential cellular processes, such as coordination of DNA repair or gene expression [309,312]. Chromatin bound proteins are subject to (often multiple) post translational modifications (PTM) (including acetylation, methylation, phosphorylation, sumoylation) [313,314,315,316]. Histone PTM can determinate higher order chromatin structure and regulate the ordered recruitment of enzyme complexes [316]. Abnormalities in histone PTM or nucleosome processing have been linked to genome instability and cancer initiation/progression [317,318]. Acetylation and methylation are two major chemical modifications affecting nucleosome status [319]. Nucleosome packing and chromatin architecture surrounding any DSB mediates the efficiency of the DDR to access and repair damaged DNA [317]. Resolution of DSBs requires coordination between the DSB machinery and chromatin remodelling complexes, to create a suitable chromatin context, allowing the correct and chronological recruitment of DDR proteins [320,321]. Importantly, open, relaxed chromatin is required for the DDR, and open and actively transcribed domains are associated with high levels of histone acetylation [317,322].

#### 3.4.1. Acetylation Inhibitors

Histone acetylation is required for many aspects of genome regulation and metabolism, and aberrant acetylation has been linked to the development of numerous diseases, including cancer (for review see [323,324,325]). Histone acetylation is associated with chromatin remodelling and transcription activation, where the acetylation moieties neutralise the positive charge of lysine residues, affecting the interaction between histones and the DNA’s negatively charged backbone. This induces a more relaxed chromatin confirmation, allowing factors access to the DNA and facilitating gene expression. Histone lysine acetylation is maintained by two opposing enzyme classes: Writers, or lysine (K) acetyl transferases (KATs; also referred to as histone acetyl transferases or HATs) which add the acetylation modification, and Erasers, which are histone deacetylases (HDACs) that remove acetylation marks [326]. Additionally, acetylation readers recognise acetylated lysine residues using bromodomains, which are present in 46 diverse nuclear and cytoplasmic proteins [327]. Readers include factors such as transcription factors that regulate gene expression, based on the chromatin context (deacetylated—closed, or acetylated—open).

#### 3.4.2. HDAC Inhibitors

The HDAC family is divided into two groups: classical Zinc (Zn)-dependent (Class I and II enzymes: which include HDACs 1–10), and Zn-independent, NAD-dependent (Class III enzymes; including HDAC11) enzymes, known as sirtuins [328]. In general, HDACs participate in large multiprotein complexes, commonly in association with co-repressors and silencing mediators [326,329]. As hyperacetylating agents, most HDAC inhibitors (HDACi) consist of three components: an active site metal binding unit, a surface recognition domain and a linker (connecting the other two domains) [330,331]. HDACis increase levels of the acetylated histones, which can induce re-expression of silenced genes and activate antitumor pathways (including growth arrest, senescence, apoptosis or autophagy) [332]. However, the mechanism of actions of many HDACi are complex and not fully understood. HDACi targeting classical Zn-dependent HDACs often contain a moiety that occupies the catalytic core of the zinc-binding site, interfering with Zn binding [333]. Chemically HDACis can be divided into different classes based on their structures: hydroxamic acids, cyclic peptide structures, benzamides or short-chain fatty acids [334,335] (Table 8).

Currently, there are three hydroxamic acid based HDACis (Vorinostat, Belinostat, Panobinostat) and a cyclic peptide HDACi (Romidepsin) with FDA approval (Table 8). These four (and several other non-FDA approved HDACis) are enrolled in clinical trials for the treatment of several cancer types [335,336]. Currently >20 clinical trials utilising HDACis are underway [337]. Hydroxamic acid-based HDACis act though the CAP region of the HDACi molecule binding to the HDAC, and it is this region that determines HDAC class selectivity. The CAP contains a connecting unit (CU), a spacer (S), and the terminal (–CONHOH) region [a Zinc-binding group (ZBG)] (for review see [337]). It is the interaction of the ZBG with the HDACs zinc ion that inhibits HDAC activity [338,339]. Vorinostat (SAHA, suberoylanilide hydroxamic acid, zolinza, L-001079038) has strong anti-proliferative activity, through the activation of tumour suppressor genes (including p21, e-cadherin) and by decreasing the expression of other key genes (including cyclin B1, c-myc, cyclin D1) which results in cell cycle arrest, apoptosis induction and cell differentiation [331,340,341]. Vorinostat was FDA approved (2006) for the treatment of refractory cutaneous T-cell lymphoma and is currently enrolled in >250 clinical trials (including three phase III) for the treatment of multiple cancer types. The sulfonamide-hydroxamide, belinostat (PXD101, Beleodaq) was FDA approved for the treatment of relapsed or refractory peripheral T-cell lymphoma and is currently enrolled in >40 clinical trials (encompassing 24 phase II trials) for the treatment of multiple tumour types. Panobinostat (LBH 589) (a cinnamic hydroxamic acid analogue) is an oral histone deacetylase inhibitor which is FDA approved (2015) for use in combination with other agents in refractory or relapsed multiple myeloma and is enrolled in >130 trials (6 phase III trials against multiple cancer types completed) [342,343] (Table 8).

Cyclic peptide-based HDACis are potent and structurally complex HDACis, with increased HDAC isoform selectivity, mediated through modification of the CAP group moiety (providing the improved specificity and activity). Cyclic peptide-based HDACi [containing a AOE (2-amino-8-oxo-9, 10-epoxy-decanoyl) moiety] can be divided into two families (based on macrocyclic moieties): cyclic tetrapeptides and bicyclic depsipeptides. Cyclic tetrapeptides are formed by a cyclic scaffold of d- and l-amino acids. Bicyclic depsipeptides display high selectivity for the Class I HDACs (HDAC1, HDAC2, HDAC3, HDAC8) and require activation by intracellular reactions (to unmask thiol- containing side chain, working as a ZBG) (for review see [337]). Romidepsin (depsipeptides, Istodax, FR 901228) is the most characterised HDACi in this family and in vitro, romidepsin primarily induces growth arrest (due to p21 expression), however cell lines with downregulated p21 undergo apoptosis [344]. Romidepsin was FDA approved (2009) for the treatment of refractory or relapsed cutaneous and peripheral T cell lymphomas (Table 8). In vivo, romidepsin induces cell cycle arrest, apoptosis and inhibits angiogenesis [345]. 

Chidamide, in the benzamide family of HDACis, was designed with molecular docking analysis and displays a high affinity for HDAC class I and HDAC class IIb. Chidamide treatment induces increased histone acetylation (H3, Lys9, 18 and H4 Lys8), inducing transcription and G0/G1 cell cycle arrest [346,347]. Chidamide is enrolled >30 clinical trials, with two in phase III trials against hormone-receptor positive breast cancer (NCT02482753) and peripheral T cell lymphoma (NCT03023358) (Table 8). The synthetic benzamide derivative, entinostat (SNDX-275, MS 275, MS 27-275) is a HDACi targeting Class 1 HDACs (HDAC1, HDAC3) and is enrolled in >50 trials for the treatment of a range of cancers. Entinostat is currently undergoing a phase III trial (NCT02115282), in combination with exemestane, for the treatment of recurrent or metastatic hormone receptor-positive breast cancer [348]. 

Finally, short-chain fatty acid HDACis have a mechanism of action that remains poorly understood—their carboxylic function may act as a ZBG, or they may compete with deacetylation reaction-released acetate [338]. Valproic acid (VPA) targets class I and IIa HDACs and is the only member of this HDACi class enrolled in clinical trials (>80 overall, five in phase III against multiple tumour types) [349]. In vivo and in vitro VPA induces the differentiation of transformed cells and can delay growth in primary tumours [350,351]. Interestingly, VPA has been used in the treatment of epilepsy for almost 30 years. 

#### 3.4.3. Histone Acetyltransferase Inhibitors

Acetylation (Ac) is the transfer of an acetylation moiety onto a substrate. The lysine (K) acetyl- transferase (KAT) or histone acetyl-transferases (HAT) family contains 17 members, with distinct families grouped based on sequence conservation in the HAT domain. HATs are divided into five families: Gcn5-related acetyltransferases (GNATs), the p300/CBP family, the general transcription factor HATs (including the TFIID subunit, TAF250), nuclear hormone-related HATs (SRC1 and ACTR/SRC3) (reviewed in [373]). The final HAT family is the largest and most divergent, the MYST family (for MOZ, YBF2, SAS2, Tip60, MOF), which are characterised by the presence of a conserved three-region histone acetyltransferase domain (containing an acetyl-CoA binding site), a zinc finger and a helix-turn-helix DNA-binding motif [323,374]. Members of the MYST family have demonstrated roles in gene regulation and most importantly, in genome stability [374]. The MYST family member, Tip60, is of particular interest, due to its accumulation at DSBs, and for its key roles in mediating the DSB DDR [acetylation and activation of ATM, histone acetylation (including H2AX) and p53 acetylation], making it a promising therapeutic target for cancer [323]. The essential role of Tip60 in the DDR has led to the search for Tip60 specific inhibitors [360,361,375]. KAT inhibitors (KATis) (hypoacetylating agents) can be divided in two classes: inhibitors discovered from library screens (such as Lys-CoA, garcinol, curcumin, anacardic acid, NU9056) or designed small molecules inhibitors (like TH1834 or H3K9me3K14CoA). However, many current KATis targeting the catalytic domain suffer from low (pan-) specificity, due to the highly conserved nature of the acetyltransferase domain (for review see [323,376,377]). 

KATis found by screening libraries of natural compounds (such as garcinol, anacardic acid or curcumin) display a significant lack of selectivity [359,378]. The natural polyphenol, curcumin, is a pan-specific KATi that can modulate multiple cell signalling pathways (including transcription) and has anti-inflammatory activity. Curcumin is enrolled in many clinical trials (>55, 3 in phase III trials) for use against a variety of cancers (Table 8). The green tea extract, EGCG (epigallocatechin-3-gallate), is another polyphenol with pan-specific HAT inhibitor activity found to inhibit acetylation in vitro, and downregulate p300 dependent acetylation in vivo [357,358]. It is currently in multiple clinical trials (>20) as a potential anticancer or chemopreventive agent (Table 8). Newer KATis targeting the MYST family (NU9056, TH1834, 6-alkylsalicylates) have shown promising results in vitro against multiple cancer types [323,360,361,363,378]. Similarity-based virtual screening identified pyridoisothiazolones, which were validated as inhibitors of p300/CBP-associated factor (PCAF) in vitro [379]. Subsequently, two pyridoisothiazolone analogue inhibitors, PU139 [targeting PCAF, Gcn5, p300 and CREB (cAMP response element-binding) protein (CBP)] and PU141 (inhibiting CBP and p300) were demonstrated to have activity in vitro against a variety of tumour cell lines [362]. In vivo, PU139 and PU141 inhibited the growth of neuroblastoma xenografts in mice. Additionally, in vivo, PU139 sensitised tumours to doxorubicin treatment. CPTH6 [3-methylcyclopentylidene-[4-(4′-chlorophenyl)thiazol-2-yl] hydrazine] is a thiazole derivative inhibitor of pCAF and Gcn5 in vitro [363,380]. In vivo, CPTH6 demonstrated its efficacy by inhibiting lung cancer stem-like cells (LCSCs) in a xenograft model [381]. The natural compound, plumbagin (RTK1), a hydroxynaph-thoquinone, was found to potently inhibit p300, both in vitro and in vivo [364] (Table 8).

#### 3.4.4. Methylation Inhibitors

Histone methylation (Me) is the addition of a methyl moiety onto a lysine or arginine residue [382]. Histone methylation is added by histone methyltransferases (HMTs), and methyl groups are removed (from lysines or arginines) by histone demethylases (HDMs). HMTs are classified into two groups, in three families: The first group acts on lysine methyltransferases (KMT) and has two families: SET-domain containing enzymes and DOT1-like proteins. The second group has one family of arginine-N-methyltransferase (PRMTs) which methylate arginines [383,384]. HDMs are divided in lysine demethylases (KDMs) and arginine demethylases; however, the latter are not well characterised. Histone methylation plays a key role in gene regulation and in DDR [385]. Dynamic DDR-mediated methylation has been observed on many histone H3 and H4 lysine residues. Both KMTs and KDMs have been observed at DSBs [386]. Misregulation of lysine methylation is frequently observed in cancers, and the diverse nature of the regulatory defects indicates that a proper equilibrium requires a precise balance between HDMs and HMTs. Targeted disruption of histone methylation (in the DDR) is a potential therapeutic option through the development of KMT and KDM inhibitors [380,387,388].

#### 3.4.5. KMT Inhibitors

The initial KMT inhibitors were analogues of SAM (S-adenosyl methionine), a common co-substrate involved in the transmethylation reaction. The bound SAM cofactor exhibited excellent shape complementarity within the pocket formed between an activation loop and the α/β-fold in the structure of the only non-SET-domain KMT known (DOT1-L). A product of this methylation process is S-adenosyl homocysteine (SAH), a known non-selective inhibitor of many methyltransferases, including DOT1-L [389]. DOT1-L is the only known HMT that specifically methylates histone H3-lysine79 (H3K79). It has been shown in myeloid/lymphoid leukaemia (MLL) that DOT1-L activity is needed for the development and maintenance of *MLL*-rearranged leukaemia. Based on the structural information of DOT1-L, the SAM mimic EPZ004777 was developed as the first SAM-competitive selective DOT1-L inhibitor [390]. EPZ004777 had high activity and selectivity for DOT1-L, inducing the selective death of MLL cells in xenograft models [391]. However, EPZ004777 had poor pharmacokinetics and did not progress to clinical trials. EPZ-5676 (an improved EPZ004777 analogue) is undergoing a phase I trial (NCT02141828) for the treatment of AML and MLL [365] (Table 8). Recently, new and novel DOT1-L inhibitors have been described, but will require further in vitro investigation to determine if they have any clinical potential [392].

KMT containing SET-domains are the class’s most abundant, with seven families identified: EZ, RIZ, SET1, SET2, SMYD, SUV4-20, SUV39 and orphan members (including SET7/9, SET8) [393]. As this subclass is implicated in tumour development, the generation of SET-domain inhibitors is of great interest. Initially inhibitors were analogues of SAM; however, several new inhibitors have been discovered (by library screening) and characterised. The selective protein lysine methyltransferase (PKMT) G9 (also known as KMT1C or EHMT2) inhibitor BIX-01294 (a diazepin-quinazolin-amine derivative) was discovered. Subsequently, the improved BIX-01294 analogues, UNC0224 and UNC0321 (40 and 250 times more potent), were developed [366]. Another analogue, UNC0638, was created by adding an isopropyl group to the piperidine, nitrogen, and replacing the diazepam ring with a cyclohexyl group, producing a more potent and less toxic inhibitor [366]. UNC0638 is highly selective for G9a and the highly related GLP, while being inactive against other KMTs (Table 8). 

The KMTs, EZH2 and EZH1, are implicated in the development of leukaemia and several types of solid tumours. EPZ005678, and its improved analogue, EPZ-6438, are potent selective SAM-competitive small molecules inhibitors of EZH2 [367]. EZH2-mutant NHL (non-Hodgkin’s lymphoma) xenograft-bearing mice treated with EPZ-6438 had significant inhibition of tumour growth. EPZ-6438 (E7438) is currently enrolled in 15 clinical trials, nine as phase II trials, against recurrent cancers (including ovarian, primary peritoneal or endometrial cancers, lymphomas, sarcomas and other advanced solid tumours) (Table 8). The KMT SMYD2 represses the activity of tumour suppressor proteins, p53 and Rb. AZ505 was found to be a potent and selective SMYD2 inhibitor, whose binding is dependent on bound SAM [368]. SETD8 (PR-SET7 or KMT5a) is responsible for the DDR related methylation of H4K20 and monomethylates non-histone substrates, including PCNA (proliferating cell nuclear antigen), which deregulates it, promoting carcinogenesis [394,395]. Nahuoic acid A was identified by high-throughput screening, and was the first selective SAM-competitive inhibitor of SETD8 [369]. Further developments led to UNC0379 (a Nahuoic acid A analogue), found to be highly selective for SETD8 (over 15 other methyltransferases, including PKMTs G9a and GLP) [18,396]. Recently, exciting new approaches led to the discovery of peptide based SETD8 inhibitors [370] (Table 8).

#### 3.4.6. KDM Inhibitors

Lysine demethylase (KDM) misregulation has been observed in many tumours, resulting in different consequences depending on the tissue or additional modulating mutations. LSD1 (lysine-specific demethylase 1) was the first identified histone demethylase acting on histones, H3K4 and H3K9. LSD1 plays key roles in cancer related pathways (including cell proliferation, chromosome segregation, cell differentiation) and can promote tumour progression by inhibiting the tumour suppressor activity of p53 [388]. Currently, two LSD1 inhibitors are enrolled in cancer clinical trials. Tranylcypromine (TCP, Transamine, Parnate) inhibits LSD1 by forming a covalent adduct with the FAD cofactor and induces growth arrest in vitro and in in vivo mouse xenograft models (breast cancer, oral squamous cell carcinoma) [397,398]. TCP is currently enrolled in two phase I/II trials for the treatment of AML (NCT02717884, NCT02273102). GSK2879552 is an orally bioavailable, irreversible and selective inhibitor binding to LSD1 with excellent pre-clinical physicochemical properties. GSK2879552 is enrolled in a trial (NCT02929498) for the treatment of High Risk Myelodysplastic Syndromes (MDS) [372,399] (Table 8).

## 4. Conclusions 

In order to advance our treatment of cancer by using truly personalised treatment regimes, we need molecular profiling (patient and tumour) combined with specific treatments targeting each tumour’s unique mutational/biochemical profile. Understanding targeted treatments molecular mechanisms of action will allow multiple treatments to be combined (with either existing treatments or with newly developed targeted treatments), to improve synergistic effects (such as avoiding resistance to single agents). Great strides have been made in the last decade in the development of compounds targeting specific pathways required for genome stability, often dysregulated or absent in many cancers. Importantly, these new inhibitors are being used to reveal exciting new fundamental molecular mechanisms of the DNA damage response, allowing us to better understand how cancer forms and progresses. 

The many promising results of targeted drug development in vitro are being applied and validated in pre-clinical models and in clinical trials, producing truly valuable new therapeutic options for many cancers. The current successes in the development and deployment of several classes of compounds (FDA/clinical approval) is being combined with molecular tumour profiling (genomic or transcriptomic), to deliver on the immense potential of targeted inhibitors to achieve truly personalised cancer medicine. Ultimately, targeted treatments will improve patient outcomes (increasing survival times) and hopefully, one day, allow many cancers to become chronic, not lethal, diseases.

## Figures and Tables

**Table 1:** Inhibitors targeting proteins involved in centrosome duplication.

Inhibitors Targeting	Enzymes	Pathways	Protein Target	Inhibitor	Mechanism of Action of Inhibitors	Clinical Trial
**Centriole disengagement**	Rad21	Centrosome duplication	Separase	Sepin-1	Inhibits Rad21 cleavage by activated separase [16,17].	Pre-clinical development [17].
**Centriole disengagement and centrosome maturation**	Plk1	Centrosome duplication	Plk1	BI 2536	2-aminopyrimidine-containing ATP-competitive kinase inhibitor. Causes disruption of the spindle assembly leading to mitotic arrest and apoptosis [18].	Phase II clinical trials completed in patients with prostatic and pancreatic neoplasms, breast cancer, endometrial cancer, head and neck cancer, non-small cell lung cancer and SCLC. https://clinicaltrials.gov/ct2/results?cond=Cancer&term=BI2536&cntry=&state=&city=&dist=
				Volasertib (BI 6727)	Blocks cell division by competitively binding to the ATP-binding pocket of Plk1 [18,19].	Phase II clinical trial completed in patients with ovarian cancer and urothelial cancer. https://clinicaltrials.gov/ct2/results?cond=Cancer&term=BI+6727&cntry=&state=&city=&dist= Phase III clinical trial in combination with low dose cytarabine in AML patients: NCT01721876.
				GSK461364	ATP-competitive Plk1 inhibitor caused mitotic arrest through G2/M arrest [18].	Phase I clinical trial completed in non-Hodgkins lymphoma: NCT00536835.
				ZK-thiazolidinone (TAL)	ATP-competitive inhibitor specifically inhibits Plk1 and causes prometaphase-like mitotic arrest [18].	Pre-clinical development [20].
**Centriole disengagement and centrosome duplication**	CDK2	Cell cycle (G1/S transition)	CDK2	Milciclib (PHA-848125 AC)	ATP-competitive inhibitor of cyclin-dependent kinases (Cdks) that potently inhibits Cdk2/cyclin A (IC50 = 45 nM) [21].	Phase I clinical trial completed in patients with advanced/metastatic solid tumours: NCT01300468. Phase II clinical trial in patients with malignant thymoma, thymic carcinoma and hepatocellular carcinoma. https://clinicaltrials.gov/ct2/results?cond=Cancer&term=PHA-848125+AC&cntry=&state=&city=&dist=
				SU9516	Inhibits pRb phosphorylation causing enhanced pRB/E2F complex formation and induces G1 and G2-M cell cycle arrest [22].	Pre-clinical development.
			CDK2 and CDK1	Butyrolactone I	An ATP-competitive inhibitor of CDKs which inhibits phosphorylation of pRB and transcription factor EF-1 and prevents cell cycle progression both at G1/S and G2/M transitions [23,24].	Pre-clinical development.
	RNA polymerase II	Cell cycle progression	Pan Cdk	Flavopiridol (Alvocidib or HMR 1275)	Causes downregulation of cyclin D1, c-MYC, and MCL-1 and induces apoptosis in tumour cells [25].	Phase II clinical trial completed in prostate cancer, kidney cancer and endometrial cancer and phase II drug combination studies completed in esophageal cancer, liver cancer pancreatic cancer, breast cancer, head and neck cancer, germ cell tumours and ovarian epithelial cancer. https://clinicaltrials.gov/ct2/results?term=Flavopiridol&cond=cancer&age_v=&gndr=&type=&rslt=&phase=1&phase=2&phase=3&Search=Apply
	pRb phosphorylation			R-547 (RG547)	ATP-competitive CDK inhibitor, inhibits retinoblastoma protein phosphorylation in tumour cells and induces apoptosis [25,26].	Phase I clinical trial completed in patients with advanced solid tumours: NCT00400296.
	Mcl-1 transcription			CYC-202 (R-roscovitine/Seliciclib)	Competes with ATP for its binding site on CDKs, reduces retinoblastoma protein phosphorylation and arrests cell cycle at G1, S, and G2-M phases [23,25,27,28].	Phase I clinical trial of CYC-202 in sequential or concomitant combination therapies in patients with breast cancer (NCT01333423), non-small cell lung cancer (NCT00372073) and advanced solid tumours (NCT00999401).
	polymerase (RNA Pol) II			SNS-032	Reversibly inhibits phosphorylation of RNA polymerase II and causes RNA synthesis inhibition [29].	Phase I clinical trial completed in patients with selected advanced solid tumours (NCT00292864) and advanced B-lymphoid malignancies (NCT00446342).
**Centriole elongation and centrosome duplication**	Plk4	Centrosome duplication	Plk4	CFI- 400945	ATP competitive inhibitor and inhibits autophosphorylation of PLK4 at serine 305 [30].	Phase I clinical trial in patients with advanced cancer (NCT01954316), and in relapsed or refractory AML or myelodysplastic syndrome (NCT03187288).
**Centrosome maturation and separation**	Aurora-A	Cell cycle (mitotic regulator) and centrosome maturation	Aurora-A	Alisertib (MLN8237)	Binds and inhibits Aurora-A, causing delayed mitotic entry and cell cycle arrest, leading to the formation of cells with tetraploid DNA content [31].	Phase II clinical trial completed in prostate cancer, childhood solid tumours or leukaemia (NCT01799278), and adult advanced non-haematological malignancies (NCT01045421). Phase II combination studies with Paclitaxel in small cell lung cancer, breast cancer and combination therapy in malignant rhabdoid tumours. https://clinicaltrials.gov/ct2/results?term=MLN8237&cond=cancer+and+neoplasia&age_v=&gndr=&type=&rslt=&phase=1&phase=2&phase=3&Search=Apply Phase III clinical trial evaluating Alisertib, compared with Pralatrexate or Gemcitabine or Romidepsin, in patients with relapsed or refractory peripheral T-Cell Lymphoma: NCT01482962.
				ENMD-2076	Causes G2-M arrest and a decrease in the intercentrosomal distance, with induction of monopolar spindles and apoptosis [32].	Phase II clinical trial completed in patients with ovarian cancer, ovarian clear cell carcinomas, haematological malignancies, hepatocellular, sarcoma and triple negative breast cancer. https://clinicaltrials.gov/ct2/results?cond=Cancer&term=ENMD-2076&cntry=&state=&city=&dist=
				MK-5108 (VX-689)	Inhibits Aurora-A kinase by competitively binding to the ATP binding site [33].	Phase I clinical trial completed in patients with advanced solid tumours: NCT00543387.
				KW-2449	A multikinase inhibitor which causes downregulation of Aurora kinases and leads to G2/M arrest [34].	Phase I clinical trial in patients with acute myelogenous leukaemia (AML) myelodysplastic syndromes and chronic myelogenous leukaemia (NCT00779480, NCT00346632).
				XL228	Multi-tyrosine kinases inhibitor which inhibits Aurora-A, the T315I mutant form of the Abl protein, IGF1R, Src tyrosine kinase. It prevents tumor angiogenesis, cell proliferation, and metastasis [35].	Phase I clinical trial in patients with advanced malignancies (NCT00526838), and chronic myeloid leukaemia or philadelphia-chromosome-positive acute lymphocytic leukaemia (NCT00464113).
				MLN8054	An ATP-competitive inhibitor which targets Aurora-A and causes monopolar, acentriolar bipolar, and multipolar spindles, leading to chromosomal segregation defects, aneuploidy and cell death [36].	Phase I clinical trials in patients with advanced malignancies (NCT00652158) and breast, colon, pancreatic and bladder tumours (NCT00249301).
	Aurora-B	Cell cycle (sister chromatid cohesion)	Aurora-B	Barasertib (AZD1152-HQPA)	Competitively blocks the ATP-binding pocket of Aurora-B kinase [31,33,37].	Phase I clinical trial completed in patients with AML (NCT01019161, NCT00926731). Phase I/II clinical trial in in patients with relapsed acute myeloid leukaemia/high-risk myelodysplastic syndrome (NCT03217838).
				BI811283	Inhibits by binding to the ATP binding pocket of Aurora-B [33].	Phase I clinical trial completed in patients with various solid tumours: NCT00701324. Phase I/IIa combination study with Cytarabine completed in patients with previously untreated acute myeloid leukaemia: NCT00701324.
	Aurora-A/B/C	Cell cycle	(Pan Aurora) Aurora-A/B/C	Danusertib (PHA-739358)	ATP competitive pan-Aurora kinase inhibitor that inhibits the catalytic domain of Aurora kinases [31,33].	Phase II study completed in patients with metastatic hormone refractory prostate cancer (NCT00766324), multiple myeloma (NCT00872300) and leukaemia (NCT00335868).
	Aurora-A/B		Aurora-A/B	PF-03814735	ATP competitive reversible inhibitor which blocks cytokinesis, resulting in decreased cell proliferation and the appearance of polyploid multinucleate cells [31,33,38].	Phase I clinical trial completed in patients with advanced solid tumours: NCT00424632.
	Aurora-A/B/C		Aurora-A/B/C	AMG 900	ATP-competitive inhibitor of Aurora kinases, causing inhibition of autophosphorylation of Aurora-A and -B [31,33].	Phase I clinical trial completed in active study in advanced malignancy and solid tumours (NCT00858377) and acute myeloid leukaemia (NCT01380756).
**Linker dissociation**	NIMA Related Kinase 2 (Nek2)	Centrosome duplication	Nek2	JH295	Irreversibly inhibits Nek2 via alkylation of residue Cys22 without affecting the mitotic kinases, Cdk1, Aurora-B, or Plk1 [39].	Pre-clinical development.
				NCL 00017509	Binds to ATP domain of Nek2 and causes irreversible inhibition [40].	Pre-clinical development.
**Centrosome separation and spindle fibre formation**	Cdk1	Cell cycle (G2/M transition)	CDK1/cyclin B1	RO-3306	Binds to ATP binding pocket and inhibits CDK1/cyclin B1 [41,42].	Pre-clinical development.
			CDK1	CGP 74514A	Binds to ATP binding pocket and reduces Akt phosphorylation, increasing mitochondrial damage and inducing apoptosis [43,44].	Pre-clinical development.
	KSP/Eg5	(Cell cycle) mitotic spindle pole separation	KSP/Eg5	Monastrol	ATP non-competitive reversible inhibitor which binds to the Eg5-ADP complex and prevents force generation and kinesin motility [45].	Pre-clinical development.
				Ispinesib (SB-715992)	ATP non-competitive reversible inhibitor which binds to the Eg5-ADP complex and prevents force generation and kinesin motility [46,47].	Phase II clinical trial completed in patients with breast cancer, prostate cancer, ovarian cancer, non-small cell lung cancer, liver cancer, kidney cancer, colorectal cancer and melanoma. https://clinicaltrials.gov/ct2/results?cond=cancer&term=Ispinesib+SB-715992&cntry=&state=&city=&dist=
				MK-0731	ATP non-competitive reversible inhibitor which binds to the Eg5-ADP complex and prevents force generation and kinesin motility [45].	Phase I clinical trial completed in patients with advanced solid tumours: NCT00104364.
	KSP/Eg5 mcl-1		KSP/Eg5 mcl-1	Filanesib (ARRY-520)	Non-ATP competitive inhibitor which binds to Eg5 at the same site as monastrol and induces cell cycle arrest at mitosis, leading to apoptosis [45,48].	Phase I/II clinical trial completed in patients with advanced solid tumours and haematological malignancies. https://clinicaltrials.gov/ct2/results?cond=cancer&term=ARRY-520&cntry=&state=&city=&dist=

**Table 2:** Inhibitors targeting proteins involved in centrosome amplification (CA).

Inhibitors Targeting	Enzymes	Pathways	Protein Target	Inhibitor	Mechanism of Action of Inhibitors	Clinical Trial
**Centrosome amplification** **(CA)**	ATM/ATR kinases	DNA damage	ATM/ATR kinases	Caffeine	Induces G1/S arrest and abrogates the G1/S and G2/M checkpoint delay periods [84].	Phase I–IV studies in a wide range of solid tumours (lymphoma, small cell lung cancer, melanoma, kidney, pancreatic, ovarian and leukaemia). https://clinicaltrials.gov/ct2/results?cond=Cancer&term=caffeine&cntry=&state=&city=&dist=
	Chk1		Chk1	UCN-01	Binds the ATP-binding pocket of Chk1, resulting in accumulation of cells in G1 phase and induction of apoptosis [92].	Phase II clinical trials in lymphoma, small-cell lung cancer, melanoma, kidney, pancreatic, ovarian and leukaemia patients. https://clinicaltrials.gov/ct2/results?term=UCN-01&age_v=&gndr=&type=&rslt=&phase=1&Search=Apply
				MK-8776 (SCH 900776)	Radiosensitizes tumor cells by causing abrogation of the G2 block and DSB repair [93].	Phase II clinical trial completed in patients with relapsed acute myeloid leukaemia: NCT01870596.
	poly (ADP-ribose) polymerase (PARP)		PARP-1	3-Aminobenzamide (3-AB)	1st generation PARP inhibitor: shows structural similarity with nicotinimide and binds PARP preventing it from depleting NAD+. PARP-1 inhibition causes the uncoupling of DNA and centrosome duplication cycles leading to CA [87].	Pre-clinical development [89].
				Rucaparib (AG14361)	Hydrogen bonds with the Gly863, Ser904, and Glu988 residues of the PARP-1 protein [91].	Phase I/II/III trials in a range of human malignancies alone and in combination with other agents. https://clinicaltrials.gov/ct2/results?cond=Cancer&term=rucaparib&cntry=&state=&city=&dist=
				NU1025	Hydrogen bonds with the Gly863, Ser904, and Glu988 residues of the PARP-1 protein [91].	Pre-clinical development [88].
	Phosphoinositide 3-kinase (PI3K)	PI3K/Akt	PI3K/p110α	LY294002	Reversibly inhibits PI3K by competing with ATP for the active site of catalytic subunit p110 [94].	Phase I clinical trial in patients with relapsed or refractory neuroblastoma: NCT02337309.
				GDC-0941 Pictrelisib	Selectively binds to PI3K isoforms in an ATP-competitive manner and inhibits the production of the secondary messenger phosphatidylinositol-3,4,5-trisphosphate (PIP3) [95].	Phase II clinical study in completed in breast cancer and non-small cell lung cancer. https://clinicaltrials.gov/ct2/results?term=GDC-0941&cond=cancer+and+neoplasia&age_v=&gndr=&type=&rslt=&phase=1&Search=Apply
				Wortmannin	Binds to the ATP-binding site of p110 by forming a covalent bond between C20 of the wortmannin furan ring and K802 of p110a [94].	Pre-clinical development.
				A66	Blocks insulin signalling to Akt/PKB by inhibiting p110α and reducing cell growth [96].	Pre-clinical development [97].
	Akt		Akt	MK-2206	Non-ATP competitive inhibitor of the PI3K/Akt signalling pathway causing decreased cell proliferation and induction of apoptosis [96].	Clinical trials in advanced breast cancer, metastatic neuroendocrine tumors (NET), advanced colorectal carcinoma, ovarian cancer, endometrial cancer and non-small cell lung cancer. https://clinicaltrials.gov/ct2/results?cond=cancer&term=MK-2206&cntry=&state=&city=&dist=&Search=Search
	Mtor p70S6 kinase			Akti X	Inhibits phosphorylation of mTOR, p70S6 kinase and S6 ribosomal protein resulting in apoptosis [96].	Phase I clinical trial in cancer patients with metastatic melanoma: NCT02489266.
	PI3K /Mtor			PF-04691502	ATP-competitive PI3K/mTOR dual inhibitor, which potently inhibits recombinant class I PI3K and mTOR [98].	Phase II clinical trial in patients with breast cancer (NCT01658176, NCT01430585) and endometrial cancer (NCT01420081).
	ROCK1 and ROCK2	RhoA/ROCK	ROCK	Y27632	Y-27632 inhibits both ROCK1 and ROCK2 by competing with ATP for binding to the catalytic site [96,99].	Pre-clinical trials [99].
				H1152	ATP-competitive inhibitor of G-protein Rho-associated [96,99].	Pre-clinical trials [99].
	Plk4	Centriole duplication	Plk4	CFI-400945	ATP competitive inhibitor, inhibits autophosphorylation of Plk4 at serine 305 [30].	Phase I clinical trial in patients with advanced cancer (NCT01954316) and phase I study in patients with relapsed or refractory acute myeloid leukaemia or myelodysplastic syndrome (NCT03187288).

**Table 3:** Inhibitors targeting proteins involved in centrosome clustering (CC).

Inhibitors Targeting	Enzymes	Pathways	Protein Target	Inhibitor	Mechanism of Action of Inhibitors	Clinical Trial
**Centrosome** **Clustering** **(CC)**	Stathmin	STAT3–Stathmin	STAT3	Stattic	Blocks STAT3 to inhibit stathmin depolymerase function, allowing stathmin to remain active to depolymerize microtubules [112].	Pre-clinical development.
				Napabucasin(BBI-608)	Blocks STAT3 to inhibit stathmin depolymerase function, allowing stathmin to remain active to depolymerize microtubules [112].	Phase I-III clinical trials including trials in combination with other compounds in patients with pancreatic, metastatic colorectal cancer, gastric, gastro-esophageal junction cancer and non-squamous, non-small cell lung cancer. https://clinicaltrials.gov/ct2/results?cond=cancer&term=BBI-608&cntry=&state=&city=&dist=&Search=Search
	APC/C Cdc20/CDH1	Mitotic progression	Anaphase-promoting Complex	ProTAME	Disrupts APC3–Cdc20 IR-tail binding interaction and prevents its activation by Cdc20 and Cdh1 [119,120].	Pre-clinical development.
	APC/C Cdc20			Apcin	Disrupts D-box interaction between Cdc20 and the substrate [120].	Pre-clinical development [122].
	HSET	Mitotic spindle assembly	HSET	CW069	Binds to loop 5 cleft of HSET motor domain causing selective allosteric inhibition of HSET [45,123].	Pre-clinical development.
				AZ82	Blocks the ATP binding pocket and binds specifically to the KIFC1/microtubule (MT) binary complex, inhibiting the MT-stimulated KIFC1 enzymatic activity [45].	Pre-clinical development [124,125].
	α/β Tubulin	microtubule dynamics	Tubulin	Griseofulvin	Decreases the dynamicity of microtubules, leading to multipolar spindles and inducing mitotic arrest [126].	Preclinical development [127].
				GF-15	Disrupts microtubule dynamics, leading to multipolar spindles [128].	Pre-clinical development [128,129].
	Poly (ADP-ribose) polymerase	DNA damage	PARP5a (TNKS1) PARP1, 2, 6	AZ0108	NAD+ competitive inhibitor [130].	Pre-clinical development [130].
			PARP-1	PJ-34	Induces G2/M arrest in cancer cells via p21 gene activation and subsequent cell death via centrosome declustering [124].	Pre-clinical development [131,132].
			PARP-1, 2	Veliparib (ABT-888)	Inhibits PARP catalytic activity and, to a lesser extent, exerts PARP trapping activity [133].	Phase I/II/III trials in a range of human malignancies, alone and in combination with other agents. https://clinicaltrials.gov/ct2/results?term=veliparib&cond=Cancer&age_v=&gndr=&type=&rslt=&phase=2&phase=3&Search=Apply
			Non-specific PARP inhibitor/cysteine-containing proteins	Iniparib (BSI-201)	A non-selective modifier of cysteine-containing proteins (protein-reactive compound), rather than a bona fide PARP inhibitor [134,135].	Phase I/II/III trials in breast, ovarian, uterine, lung and advanced solid tumours, both alone and in combination with other agents. https://clinicaltrials.gov/ct2/results?cond=Cancer&term=Iniparib&cntry=&state=&city=&dist=
	Coiled-Coil Containing Protein 3 (TACC3) Colonic and Hepatic Tumour Overexpressed gene (ch-TOG)	Actin and mitotic microtubule organization	Integrin-linked Kinase (ILK)	QLT-0267	Inhibits kinase activity of ILK in an ATP-competitive manner and disrupts TACC3 phosphorylation [129].	Pre-clinical development [136].
	Hsp70	Nek6–Hsp72	Hsp70 Hsp72	VER-155008	ATP competitive inhibitor of Hsp 70 which blocks the nucleotide-binding domain and prevents substrate binding [137].	Pre-clinical development [138].
	N/A	N/A	N/A	CCCI-01	Inhibits centrosome clustering, promoting spindle multipolarity and cell death selectively in cancer cells [139].	Pre-clinical development [139].
	End Binding Protein-1 (EB1), cytoplasmic linker protein-170 (CLIP-170)	Microtubule dynamics	EB1, CLIP-170	EM011	Disrupts microtubule dynamicity and induces G2/M arrest in cancer cells followed by apoptotic cell death [140].	Pre-clinical development [141].
	Cofilin	Actin destabilization	Cofilin Platelet-derived Growth Factor Receptor (PDGFR)-a and -b and FLT3	Crenolanib (CP-868596)	An ATP competitive inhibitor which induces spindle multipolarity in cells with CA by activating cofilin [142].	Phase I–III clinical trials in D842V PDGFRA gene-mutated tumours, advanced gastrointestinal stromal tumours and acute myeloid leukaemia. https://clinicaltrials.gov/ct2/results?cond=cancer&term=Crenolanib&cntry=&state=&city=&dist=&Search=Search
	Cofilin	Actin destabilization	Cofilin PDGFR-b	CP-673451	An ATP competitive inhibitor which induces spindle multipolarity in cells with CA by activating cofilin [142].	Pre-clinical development [143].

**Table 4:** Small Molecule Inhibitors of DNA Repair Proteins—Initiators.

Inhibitors Targeting	Enzymes	Pathways	Protein Target	Inhibitor	Mechanism of Action	Clinical Trial
ADP- ribosylation	PARP	DDR	PARP1, PARP2 and PARP3	Olaparib	Binds within the nicotinamide-binding pocket in the ADP-ribosyl transferase catalytic site [177].	2014: FDA approved for the treatment of adult patients with deleterious or suspected deleterious germline BRCA-mutated advanced ovarian cancer [183]. 2017: FDA approved for the maintenance treatment of adult patients with recurrent epithelial ovarian, fallopian tube or primary peritoneal cancer, who are in a complete or partial response to platinum-based chemotherapy. https://www.fda.gov/Drugs/InformationOnDrugs/ApprovedDrugs/ucm572143.htm 2018: FDA approved for the treatment of adult patients with metastatic breast cancer who have a BRCA gene mutation. https://www.fda.gov/newsevents/newsroom/pressannouncements/ucm592347.htm
				Rucaparib	Binds within the nicotinamide-binding pocket in the ADP-ribosyl transferase catalytic site [177].	2016: FDA approved for treatment of adults patients with germline and/or somatic BRCA-mutated advanced ovarian cancer. https://www.fda.gov/Drugs/InformationOnDrugs/ApprovedDrugs/ucm533891.htm
			PARP1 and PARP2	Niraparib	Binds within the nicotinamide-binding pocket in the ADP-ribosyl transferase catalytic site and makes contact with the regulatory subdomains. Efficiently traps PARP1 on the damage-containing DNA [177].	2017: FDA approved for treatment of adult patients with recurrent epithelial ovarian, fallopian tube or primary peritoneal cancer who are in complete or partial response to platinum-based chemotherapy. https://www.fda.gov/Drugs/InformationOnDrugs/ApprovedDrugs/ucm548487.htm
				Veliparib	Binds within nicotinamide-binding pocket in the ADP-ribosyl transferase catalytic site and makes contact with the regulatory subdomains. Efficiently traps PARP1 on the damage-containing DNA [177].	Phase I-III clinical trials including patients with previously untreated advanced or metastatic squamous non-small cell lung cancer; patients receiving first cytotoxic chemotherapy for metastatic or advanced non-squamous, non-small cell lung cancer; patients with ovarian cancer; triple negative breast cancer; glioblastoma. Mostly in combination with chemotherapy. https://clinicaltrials.gov/ct2/results?term=Veliparib&age_v=&gndr=&type=&rslt=&phase=0&phase=1&phase=2&phase=3&Search=Apply
				Talazoparib	Binds within nicotinamide-binding pocket in the ADP-ribosyl transferase catalytic site and makes contact with the regulatory subdomains. Potent PARP trapping [177].	Phase I–III clinical trials including phase III patients with advanced and/or metastatic breast cancer with germline BRCA (breast cancer susceptibility gene) mutations and squamous cell lung carcinoma. https://clinicaltrials.gov/ct2/results?term=Talazoparib&age_v=&gndr=&type=&rslt=&phase=0&phase=1&phase=2&phase=3&Search=Apply
				CEP-9722	Binds within the nicotinamide-binding pocket in the ADP-ribosyl transferase catalytic site [177,179].	Phase I/II trial in patients with advanced or metastatic solid tumours and documented deficiencies of DNA repair pathways, such as BRCA1/2 (NCT01311713, NCT01345357, NCT00920595).
Phosphorylation	PIKK	DSB, cell cycle	ATM	KU-55933	Binds to the ATP binding pocket of ATM, blocking its kinase function and ATM-mediated signalling [170].	Pre-clinical development.
				KU-60019	Binds to the ATP binding pocket of ATM, blocking its kinase function and ATM-mediated signalling [184].	Pre-clinical development.
				KU-59403	Binds to the ATP binding pocket of ATM, blocking its kinase function and ATM-mediated signalling [185].	Pre-clinical development.
				CP466722	Binds to the ATP binding pocket of ATM, blocking its kinase function and ATM-mediated signalling [185].	Pre-clinical development.
				AZD0156	Binds to the ATP binding pocket of ATM, blocking its kinase function and ATM-mediated signalling [186].	Phase I trial of AZD0156 in combination with olaparib in patients with locally advanced/metastatic cancer: NCT02588105.
		SSB, Cell Cycle	ATR	VE-822/VX-970	Selectively inhibits ATR kinase activity and prevents ATR-mediated signalling in the ATR-checkpoint kinase 1 (Chk1) signalling pathway [187].	Phase I/II trial of VX970 and topotecan treating small cell lung cancer: NCT02487095. Phase I trial of VX970 in combination with veliparib and cisplatin in patients with advanced refractory solid tumours: NCT02723864 Phase I trial of VX970 and irinotecan hydrochloride in treating patients with metastatic cancer: NCT02595931.
				AZD6738	Selectively inhibits ATR kinase activity and prevents ATR-mediated signalling [188].	Phase I/II trial for AZD6738 in combination with acalabrutinib in subjects with relapse or refractory high-risk chronic lymphocytic leukaemia (CLL): NCT03328273. Phase I trial for AZD6738 in combination with palliative radiotherapy or chemotherapy in patients with advanced solid tumours: NCT02223923.
				BAY-1895344	Selectively inhibits ATR kinase activity and prevents ATR-mediated signalling in the ATR-checkpoint kinase 1 (Chk1) signalling pathway [189].	Phase I trial of BAY1895344 monotherapy in patients with advanced solid tumours and lymphomas: NCT03188965.
			ATR/CDK2	NU6027	Low micromolar inhibitor of ATR kinase activity and prevents ATR-mediated signalling in the ATR-checkpoint kinase 1 (Chk1) signalling pathway.	Pre-clinical development.

**Table 5:** Small Molecule Inhibitors of DNA Repair Proteins—Effectors.

Inhibitors Targeting	Enzymes	Pathways	Protein Target	Inhibitor	Mechanism of Action	Clinical Trial
DNA processing	RecQ DNA Helicases	DDR	BLM	ML216	Inhibits helicase activity of BLM. ML216 competes with ATP binding, the driving force behind its DNA unwinding, or by preventing BLM from binding to DNA [218].	Pre-clinical development.
			WRN	NSC 617145	Inhibits WRN helicase activity, but not its nuclease activity. Thought to trap WRN on the DNA substrate [219].	Pre-clinical development.
				NSC 19630	Inhibits helicase activity, but not nuclease activity [221].	Pre-clinical development.
	Topoisomerases	DDR, DNA replication	TOP1	Irinotecan	Prevents religation of the DNA strand by binding to topoisomerase I-DNA complex [226].	FDA approved (1996) for treatment of colorectal cancer when disease has recurred following initial fluorouracil treatment. FDA approved (2015) in combination with fluorouracil and leucovorin, in patients with advanced (metastatic) pancreatic cancer previously treated with gemcitabine-based chemotherapy. https://www.accessdata.fda.gov/scripts/cder/ob/search_product.cfm
				Topotecan	Binds to the topoisomerase I-DNA complex and prevents re-ligation of single strand breaks [226].	FDA approved (2006) in combination with cisplatin for the treatment of stage IVB recurrent or persistent cervical cancer that is not amenable to curative treatment with surgery and/or radiotherapy. https://www.cancer.gov/about-cancer/treatment/drugs/fda-topotecan-hydrochloride Phase III trial in combination with radiotherapy in patients with brain metastases from non-small cell lung cancer: NCT00390806.
				Indotecan (LMP400)	Binds to the topoisomerase I-DNA covalent cleavage complexes, and inhibits repair of single-strand breaks [223].	Phase I trial of LMP400 in subjects with solid tumours or lymphomas that have not responded to treatment: NCT01794104.
				Indimitecan (LMP776)	Preferential Top1-DNA trapping at unique sites [232].	Phase I trial in adults with relapsed solid tumors and lymphomas: NCT01051635.
				GENZ-644282	Binds to the topoisomerase I-DNA covalent cleavage complexes, and inhibits repair of single-strand breaks [228].	Phase I trial of Genz-644282 in patients with advanced malignant, solid tumours: NCT00942799.
			TOP2	Doxorubicin	Intercalates into DNA and targets the topoisomerase II cleavage complexes, thereby inhibiting DNA religation [223].	FDA approved (1974) and currently used for treatment for acute lymphoblastic leukaemia, acute myeloblastic leukaemia, Wilms’ tumour, neuroblastoma, breast carcinoma, ovarian carcinoma, transitional cell bladder carcinoma, thyroid carcinoma, gastric carcinoma, Hodgkin’s disease, malignant lymphoma and bronchogenic carcinoma [233].
				Etoposide	Intercalates into DNA and poisons the topoisomerase II cleavage complexes, thereby inhibiting DNA re-ligation [223].	FDA approved (1983) and currently used in combination with other chemotherapeutic drugs for treatment of patients with refractory testicular tumours, small lung cancer, ovarian cancer, leukaemia and lymphoma. https://www.accessdata.fda.gov/scripts/cder/daf/index.cfm?event=overview.process&applno=074290
				Mitoxantrone	Intercalates into DNA, causing crosslinks and strand breaks and targets the topoisomerase II cleavage complexes, thereby inhibiting DNA re-ligation [223].	FDA approved (1987) and currently used with other drugs to treat acute myeloid leukaemia and prostate cancer. https://www.accessdata.fda.gov/scripts/cder/daf/index.cfm?event=overview.process&applno=077356 https://www.cancer.gov/about-cancer/treatment/drugs/mitoxantronehydrochloride
				Aclarubicin	Intercalates into DNA and targets the topoisomerase II cleavage complexes, inhibiting DNA re-ligation [223].	Phase II–IV trials in combination with other drugs in patients with acute myeloid leukaemia (AML). https://clinicaltrials.gov/ct2/results?cond=cancer&term=Aclarubicin&cntry=&state=&city=&dist=
				Dexrazoxane (ICRF-187)	Catalytic TOP2 inhibitor.	Phase III–IV trials against multiple cancers. https://clinicaltrials.gov/ct2/results?cond=Cancer&term=Dexrazoxane&cntry=&state=&city=&dist=&Search=Search
DNA repair signalling	MRN complex	DDR	Mre11	Mirin	Binds in the active site of Mre11 blocking DNA phosphate backbone rotation; inhibits exonuclease activity of Mre11 and MRN/DSB-mediated ATM activation without affecting ATM protein kinase activity [234].	Pre-clinical development.
				PFM01	Binds near the dimer interface blocking the ssDNA-binding path towards the catalytic metal ions and disrupts endonuclease activity [185].	Pre-clinical development.
				PFM39	Binds in the active site of Mre11 inhibiting its exonuclease activity [185].	Pre-clinical development.
DNA repair	Nucleotide Excision Repair (NER)		ERCC1–XPF	E-X AS5-4	Targets the ERCC1–XPF interaction domain for heterodimerisation [235].	Pre-clinical development.
				E-X AS7	Targets the XPF active site itself [235].	Pre-clinical development.

**Table 6:** Small Molecule Inhibitors of DNA Repair Proteins—NHEJ.

Inhibitors Targeting	Enzymes	Pathways	Protein Target	Inhibitor	Mechanism of Action	Clinical Trial
Phosphorylation	PIKK	NHEJ	DNA-PKcs	NU7026	A potent inhibitor of DNA-PK, exhibiting ATP-competitive inhibitor kinetics [264].	Pre-clinical development.
				NU7441	A highly potent and selective DNA-PK inhibitor, exhibiting ATP-competitive inhibition kinetics [263].	Pre-clinical development.
				VX-984/M9831	ATP-competitive inhibitor of DNA-PKcs [269].	Phase 1 clinical trial as a single agent and in combination with doxorubicin or pegylated liposomal doxorubicin (PLD): NCT02644278.
				MSC-2490484A/M3814	Binds to DNA-PK and inhibits its kinase activity and prevents (at least partially) the NHEJ pathway [266].	Phase I trial of MSC2490484A monotherapy in subjects with advanced solid tumors or chronic lymphocytic leukaemia (CLL) likely to have alterations in DNA repair mechanisms, such as the BRCA and ATM pathways: NCT02316197. Phase I trial of MSC2490484A in combination with radiotherapy and in combination with chemoradiotherapy (radiotherapy and cisplatin) in patients with advanced solid tumours: NCT02516813.
DNA processing	DNA ligase		Ligases I, III and IV	L189	Equally inhibits ligases I, III and IV by blocking DNA binding [253].	Pre-clinical development.
			Ligase-IV (DNA ligases I and III)	SCR7	Binds to the DNA-binding domain of ligase-IV, thus preventing the binding of ligase IV to DNA ends [271].	Pre-clinical development.

**Table 7:** Small Molecule Inhibitors of DNA Repair Proteins—HR.

Inhibitors Targeting	Enzymes	Pathways	Protein Target	Inhibitor	Mechanism of Action	Clinical Trial
DNA processing	RecA-like NTPases	HR	RAD51	B02	Inhibits RAD51 by disrupting RAD51 ability to bind ssDNA [280].	Pre-clinical development.
				RI-1	Contains a chloromaleimide group that reacts with cysteine 319 of RAD51 at the monomer–monomer interface near the ATP active site [281].	Pre-clinical development.
				RS-1	Stabilizes the RAD51 nucleoprotein filament and stimulates RAD51 biochemical activities [282].	Pre-clinical development.
				IBR120	Inhibits RAD51 by mimicking the effect of BRC repeat binding to RAD51 [283].	Pre-clinical development.
	RAD52		RAD52	D-103	Inhibits RAD52-mediated ssDNA annealing and inhibits D-loop formation [284].	Pre-clinical development.
				D-G23	Inhibits RAD52-mediated ssDNA annealing and inhibits D-loop formation [284].	Pre-clinical development.
				AICAR	Disrupts the RAD52-ssDNA interaction [285].	Pre-clinical development.
				(−)-Epigallocatechin)	Inhibits RAD52 ssDNA binding [286].	Pre-clinical development.
				6-HidroxyDL-dopa	Disrupts RAD52 recruitment and recombination activity [287].	Pre-clinical development.
	RAD54		RAD54/SENP1	Streptonigrin (STN)	An antitumor antibiotic that binds RAD54 ATPase domain and inactivates it by generating reactive oxygen species [288]. Recently found to bind to and inhibit SUMO-specific protease, SENP1 [289].	Used to treat multiple cancer types (since the 1960s); however, induces severe and prolonged bone marrow depression.

**Table 8:** Small Molecule Inhibitors of DNA Repair Proteins—Chromatin Modification.

Inhibitors Targeting	Enzymes	Pathways	Protein Target	Inhibitor	Mechanism of Action	Clinical Trial
Acetylation	HDAC	Chromatin modification and DDR	Histone deacetylases (HDACs) I, IIa, IIb, IV	Vorinostat/SAHA	Inhibits HDAC by binding the zinc-activated catalytic site [352].	FDA approved (2006) for the treatment of cutaneous manifestations of T-cell lymphoma [352]. Phase III trial of vorinostat in the treatment of advanced malignant pleural mesothelioma and multiple myeloma: NCT00128102. Phase III trial in combination with chemotherapy for the treatment of advanced non-small cell lung cancer patients: NCT00473889.
			HDACs I, II	Belinostat	Inhibits HDAC by binding to the zinc-activated catalytic site [353].	FDA approved (2014) for the treatment of patients with relapsed or refractory peripheral T-cell lymphoma [353]. Phase I–II clinical trials in the treatment of a range of solid tumours, acute myeloid leukaemia, cutaneous T-cell lymphoma, lung and liver cancer and non-Hodgkins lymphoma and other haematological malignancies. https://clinicaltrials.gov/ct2/results?term=belinostat&age_v=&gndr=&type=&rslt=&Search=Apply
			HDACs I, II	Panobinostat	Inhibits HDAC by binding to the zinc-activated catalytic site [354].	FDA approved (2015) for the treatment of patients with multiple myeloma. https://www.accessdata.fda.gov/scripts/cder/ob/search_product.cfm Phase I–III trials in the treatment of a range of cancers, including pancreatic, breast, lung, liver, prostate, thyroid, renal, colon, brain, gastric, skin, and haematological malignancies. https://clinicaltrials.gov/ct2/results?term=panobinostat+AND+Cancer+AND+Neoplasms&phase=0123
			HDACs 1,2,4,6	Romidepsin	inhibits HDAC by binding to the zinc-activated catalytic site [345].	FDA approved (2009) for the treatment of cutaneous T-cell lymphoma in patients who have received at least one prior systemic therapy. 2011: FDA approved for the treatment of peripheral T-cell lymphoma in patients who have received at least one prior therapy [355].
			HDACs 1,2,3,10	Chidamide	Inhibits HDAC by binding to the zinc-activated catalytic site [356].	Phase III trial in combination with exemestane for the treatment of hormone-receptor positive advanced breast cancer: NCT02482753. Phase III trial in combination with chemotherapy for the treatment of peripheral T-cell lymphoma: NCT03023358.
			HDACs I, IIa	Valproic acid (VPA)	In vivo and in vitro induces differentiation of transformed cells and can delay growth in primary tumours [350,351,357,358].	Enrolled in >80 clinical cancer trials, including five in phase III (for multiple tumour types). https://clinicaltrials.gov/ct2/results?cond=Cancer&term=Valproic+acid&cntry=&state=&city=&dist=
	histone acetyl transferases (HAT)		p300/CBP, PCAF, Tip60	Curcumin	Inhibits p300/CBP by decreasing the binding efficiency of both histones and acetyl CoA to p300 [359].	Phase I–III clinical trials for the treatment of multiple tumour types. https://clinicaltrials.gov/ct2/results?cond=Cancer&term=Curcumin+&cntry=&state=&city=&dist=
			p300, CBP, Tip60, PCAF	EGCG	Did not appear bind to the HAT domain, potentially binds another site on the protein [357].	Phase I–IV clinical trials in a range of tumours including breast, prostate, colon, lung, pancreas. https://clinicaltrials.gov/ct2/results?cond=Cancer&term=EGCG&cntry=&state=&city=&dist=
			Tip60	TH1834	Binds into the AcCoA binding pocket [360].	Pre-clinical development.
			Tip60	NU9056	Binds into the AcCoA binding pocket [361].	Pre-clinical development.
			PCAF, Gcn5, p300 CREB	PU139	Predicted to bind at the catalytic site binding pocket [362].	Pre-clinical development.
			CBP, p300	PU141	Predicted to bind at the catalytic site binding pocket [362].	Pre-clinical development.
			PCAF, Gcn5	CPTH6	Competes with Acetyl-CoA to bind at the catalytic site [363].	Pre-clinical development.
			p300	RTK1	Through its hydroxyl group, possibly forms a specific interaction with lysine residue (Lys-1358) in the p300 HAT domain [364].	Pre-clinical development.
Methylation	KMT		DOT1-L	EPZ-5676	Occupies the S-adenosyl methionine (SAM) binding pocket of DOT1-L [365].	Phase I trial for the treatment of acute myeloid leukaemia (AML) and acute lymphoblastic leukaemia (ALL): NCT02141828.
			G9a	UNC0638	Occupies the histone peptide-binding channel and interacts with the lysine-binding pocket [366].	Pre-clinical development.
			EZH2	EPZ-6438 (tazemetostat)	Occupies the S-adenosyl methionine (SAM) binding pocket of EZH2 [367].	Phase I–II clinical trials for the treatment of recurrent ovarian, primary peritoneal, or endometrial cancer, different types of lymphomas, sarcomas and advanced solid tumours. https://clinicaltrials.gov/ct2/results?term=EPZ-6438&age_v=&gndr=&type=&rslt=&phase=0&phase=1&phase=2&phase=3&Search=Apply
			SMYD2	AZ505	Inhibits though its benzooxazinone group, which is positioned within the lysine-binding channel of the substrate [368].	Pre-clinical development.
			SETD8	Nahuoic acid A	Occupies the S-adenosyl methionine (SAM) binding pocket [369].	Pre-clinical development.
			SETD8	Peptide based inhibitors	Selective norleucine containing peptide inhibitor [370]	Pre-clinical development.
	KDM		LSD1	TCP (tranylcypromine)	Inhibits LSD1 by forming a covalent adduct with the FAD cofactor [371].	Phase I/II trial in combination with ATRA (all-trans-retinoic acid) for the treatment of acute myeloid leukaemia or myelodysplastic syndrome (NCT02717884, NCT02273102).
				GSK2879552	Inhibits LSD1 by forming a covalent adduct with the FAD cofactor, leading to homolytic cleavage of the cyclopropyl ring [372].	Phase I trial for the treatment of myelocytic leukaemia (NCT02177812) and small cell carcinoma (NCT02034123). Phase II trial in combination with azacitidine for the treatment of myelodysplastic syndrome: NCT02929498.

## Abbreviations

The following abbreviations are used in this manuscript:

DDR	DNA damage response
FDA	Food and Drug Administration (United States)
HAT	Histone acetyltransferase
HATi	HAT inhibitors
HDAC	Histone de-acetyltransferase
HDACi	Histone de-acetyltransferase inhibitor
HMT	Histone methyltransferases
KDM	Lysine demethylases
DSB	Double strand break
SSB	Single Strand Break
SSA	Single Strand Annealing
HR	Homologous recombination
NHEJ	Non-Homologous End Joining
CA	Centrosome Amplification
PARPi	PARP inhibitors
ATM	Ataxia-telangiectasia mutated
ATR	Ataxia and Rad3-related
PIKK	Phosphatidylinositol 3-kinase-related kinase
IR	Ionizing radiation
FA	Fanconi Anemia
MMC	Mitomycin C
MMEJ	Microhomology-Mediated End Joining
PTM	Post translational modifications

## References

[1] Matchett K.B., Lynam-Lennon N., Watson R.W., Brown J.A.L. (2017). Advances in Precision Medicine: Tailoring Individualized Therapies. Cancers (Basal).

[2] Brown J.A.L., Ni Chonghaile T., Matchett K.B., Lynam-Lennon N., Kiely P.A. (2016). Big Data-Led Cancer Research, Application, and Insights. Cancer Res..

[3] Ciardiello F., Arnold D., Casali P.G., Cervantes A., Douillard J.-Y., Eggermont A., Eniu A., McGregor K., Peters S., Piccart M.R. (2014). Delivering precision medicine in oncology today and in future-the promise and challenges of personalised cancer medicine: A position paper by the European Society for Medical Oncology (ESMO). Ann. Oncol..

[4] Girotti M.R., Gremel G., Lee R., Galvani E., Rothwell D., Viros A., Mandal A.K., Lim K.H.J., Saturno G., Furney S.J. (2016). Application of Sequencing, Liquid Biopsies, and Patient-Derived Xenografts for Personalized Medicine in Melanoma. Cancer Discov..

[5] Negrini S., Gorgoulis V.G., Halazonetis T.D. (2010). Genomic instability—An evolving hallmark of cancer. Nat. Rev. Mol. Cell Biol..

[6] Aguilera A., Gómez-González B. (2008). Genome instability: A mechanistic view of its causes and consequences. Nat. Rev. Genet..

[7] Mao Z., Bozzella M., Seluanov A., Gorbunova V. (2008). Comparison of nonhomologous end joining and homologous recombination in human cells. DNA Repair (Amst.).

[8] Conduit P.T., Wainman A., Raff J.W. (2015). Centrosome function and assembly in animal cells. Nat. Rev. Mol. Cell Biol..

[9] Lerit D.A., Poulton J.S. (2016). Centrosomes are multifunctional regulators of genome stability. Chromosome Res..

[10] Bornens M. (2012). The centrosome in cells and organisms. Science.

[11] O’Connor M.J. (2015). Targeting the DNA Damage Response in Cancer. Mol. Cell.

[12] Nigg E.A., Holland A.J. (2018). Once and only once: Mechanisms of centriole duplication and their deregulation in disease. Nat. Rev. Mol. Cell Biol..

[13] Serçin Ö., Larsimont J.-C., Karambelas A.E., Marthiens V., Moers V., Boeckx B., Mercier M.L., Lambrechts D., Basto R., Blanpain C. (2015). Transient PLK4 overexpression accelerates tumorigenesis in p53-deficient epidermis. Nat. Cell Biol..

[14] Levine M.S., Bakker B., Boeckx B., Moyett J., Lu J., Vitre B., Spierings D.C., Lansdorp P.M., Cleveland D.W., Lambrechts D. (2017). Centrosome Amplification Is Sufficient to Promote Spontaneous Tumorigenesis in Mammals. Dev. Cell.

[15] Chan J.Y. (2011). A clinical overview of centrosome amplification in human cancers. Int. J. Biol. Sci..

[16] Zhang N., Scorsone K., Ge G., Kaffes C.C., Dobrolecki L.E., Mukherjee M., Lewis M.T., Berg S., Stephan C.C., Pati D. (2014). Identification and Characterization of Separase Inhibitors (Sepins) for Cancer Therapy. J. Biomol. Screen..

[17] Do H.T., Zhang N., Pati D., Gilbertson S.R. (2016). Synthesis and activity of benzimidazole-1,3-dioxide inhibitors of separase. Bioorg. Med. Chem. Lett..

[18] Gutteridge R.E.A., Ndiaye M.A., Liu X., Ahmad N. (2016). Plk1 Inhibitors in Cancer Therapy: From Laboratory to Clinics. Mol. Cancer Ther..

[19] Cholewa B.D., Ndiaye M.A., Huang W., Liu X., Ahmad N. (2017). Small molecule inhibition of polo-like kinase 1 by volasertib (BI 6727) causes significant melanoma growth delay and regression in vivo. Cancer Lett..

[20] Kumar S., Kim J. (2015). PLK-1 Targeted Inhibitors and Their Potential against Tumorigenesis. Biomed. Res. Int..

[21] Chohan T.A., Qian H., Pan Y., Chen J.-Z. (2015). Cyclin-dependent kinase-2 as a target for cancer therapy: Progress in the development of CDK2 inhibitors as anti-cancer agents. Curr. Med. Chem..

[22] Lane M.E., Yu B., Rice A., Lipson K.E., Liang C., Sun L., Tang C., McMahon G., Pestell R.G., Wadler S. (2001). A novel cdk2-selective inhibitor, SU9516, induces apoptosis in colon carcinoma cells. Cancer Res..

[23] Matsumoto Y., Hayashi K., Nishida E. (1999). Cyclin-dependent kinase 2 (Cdk2) is required for centrosome duplication in mammalian cells. Curr. Biol. CB.

[24] Yamamoto H., Monden T., Miyoshi H., Izawa H., Ikeda K., Tsujie M., Ohnishi T., Sekimoto M., Tomita N., Monden M. (1998). Cdk2/cdc2 expression in colon carcinogenesis and effects of cdk2/cdc2 inhibitor in colon cancer cells. Int. J. Oncol..

[25] Mariaule G., Belmont P. (2014). Cyclin-dependent kinase inhibitors as marketed anticancer drugs: Where are we now? A short survey. Molecules.

[26] DePinto W., Chu X.-J., Yin X., Smith M., Packman K., Goelzer P., Lovey A., Chen Y., Qian H., Hamid R. (2006). In vitro and in vivo activity of R547: A potent and selective cyclin-dependent kinase inhibitor currently in phase I clinical trials. Mol. Cancer Ther..

[27] Whittaker S.R., Walton M.I., Garrett M.D., Workman P. (2004). The Cyclin-dependent kinase inhibitor CYC202 (R-roscovitine) inhibits retinoblastoma protein phosphorylation, causes loss of Cyclin D1, and activates the mitogen-activated protein kinase pathway. Cancer Res..

[28] Raje N. (2005). Seliciclib (CYC202 or R-roscovitine), a small-molecule cyclin-dependent kinase inhibitor, mediates activity via down-regulation of Mcl-1 in multiple myeloma. Blood.

[29] Chen R., Wierda W.G., Chubb S., Hawtin R.E., Fox J.A., Keating M.J., Gandhi V., Plunkett W. (2009). Mechanism of action of SNS-032, a novel cyclin-dependent kinase inhibitor, in chronic lymphocytic leukemia. Blood.

[30] Mason J.M., Lin D.C.-C., Wei X., Che Y., Yao Y., Kiarash R., Cescon D.W., Fletcher G.C., Awrey D.E., Bray M.R. (2014). Functional characterization of CFI-400945, a Polo-like kinase 4 inhibitor, as a potential anticancer agent. Cancer Cell.

[31] Bavetsias V., Linardopoulos S. (2015). Aurora Kinase Inhibitors: Current Status and Outlook. Front. Oncol..

[32] Fletcher G.C., Brokx R.D., Denny T.A., Hembrough T.A., Plum S.M., Fogler W.E., Sidor C.F., Bray M.R. (2011). ENMD-2076 is an orally active kinase inhibitor with antiangiogenic and antiproliferative mechanisms of action. Mol. Cancer Ther..

[33] Green M.R., Woolery J.E., Mahadevan D. (2011). Update on Aurora Kinase Targeted Therapeutics in Oncology. Expert Opin. Drug Discov..

[34] Shiotsu Y., Kiyoi H., Ishikawa Y., Tanizaki R., Shimizu M., Umehara H., Ishii K., Mori Y., Ozeki K., Minami Y. (2009). KW-2449, a novel multikinase inhibitor, suppresses the growth of leukemia cells with FLT3 mutations or T315I-mutated BCR/ABL translocation. Blood.

[35] Moore A.S., Blagg J., Linardopoulos S., Pearson A.D.J. (2010). Aurora kinase inhibitors: Novel small molecules with promising activity in acute myeloid and Philadelphia-positive leukemias. Leukemia.

[36] Hoar K., Chakravarty A., Rabino C., Wysong D., Bowman D., Roy N., Ecsedy J.A. (2007). MLN8054, a small-molecule inhibitor of Aurora A, causes spindle pole and chromosome congression defects leading to aneuploidy. Mol. Cell. Biol..

[37] Sessa F., Villa F. (2014). Structure of Aurora B-INCENP in complex with barasertib reveals a potential transinhibitory mechanism. Acta Crystallogr. F Struct. Biol. Commun..

[38] Jani J.P., Arcari J., Bernardo V., Bhattacharya S.K., Briere D., Cohen B.D., Coleman K., Christensen J.G., Emerson E.O., Jakowski A. (2010). PF-03814735, an orally bioavailable small molecule aurora kinase inhibitor for cancer therapy. Mol. Cancer Ther..

[39] Henise J.C., Taunton J. (2011). Irreversible Nek2 kinase inhibitors with cellular activity. J. Med. Chem..

[40] 40. Carbain B., Bayliss R., Boxall K., Coxon C., Lebraud H., Matheson C., Turner D., Zhen-Wang L., Griffin R.J. (2012). 188 2-arylamino-6-ethynylpurines as Potent Irreversible Inhibitors of the Mitotic Kinase Nek2. Eur. J. Cancer.

[41] Vassilev L.T., Tovar C., Chen S., Knezevic D., Zhao X., Sun H., Heimbrook D.C., Chen L. (2006). Selective small-molecule inhibitor reveals critical mitotic functions of human CDK1. Proc. Natl. Acad. Sci. USA.

[42] Steere N., Wagner M., Beishir S., Smith E., Breslin L., Morrison C.G., Hochegger H., Kuriyama R. (2011). Centrosome amplification in CHO and DT40 cells by inactivation of cyclin-dependent kinases. Cytoskeleton (Hoboken).

[43] Furet P., Zimmermann J., Capraro H.G., Meyer T., Imbach P. (2000). Structure-based design of potent CDK1 inhibitors derived from olomoucine. J. Comput. Aided Mol. Des..

[44] Dai Y., Dent P., Grant S. (2002). Induction of apoptosis in human leukemia cells by the CDK1 inhibitor CGP74514A. Cell Cycle.

[45] Myers S.M., Collins I. (2016). Recent findings and future directions for interpolar mitotic kinesin inhibitors in cancer therapy. Future Med. Chem..

[46] Sarli V., Giannis A. (2008). Targeting the kinesin spindle protein: Basic principles and clinical implications. Clin. Cancer Res..

[47] Rath O., Kozielski F. (2012). Kinesins and cancer. Nat Rev Cancer..

[48] Woessner R., Tunquist B., Lemieux C., Chlipala E., Jackinsky S., Dewolf W., Voegtli1 W., Cox1 A., Rana S., Lee P., Walker D. (2009). ARRY-520, a novel KSP inhibitor with potent activity in hematological and taxane-resistant tumor models. Anticancer Res..

[49] Mardin B.R., Schiebel E. (2012). Breaking the ties that bind: New advances in centrosome biology. J. Cell Biol..

[50] Agircan F.G., Schiebel E., Mardin B.R. (2014). Separate to operate: Control of centrosome positioning and separation. Philos. Trans. R. Soc. Lond. B Biol. Sci..

[51] Meyer R., Fofanov V., Panigrahi A., Merchant F., Zhang N., Pati D. (2009). Overexpression and mislocalization of the chromosomal segregation protein separase in multiple human cancers. Clin. Cancer Res..

[52] Kishi K., van Vugt M.A.T.M., Okamoto K.-I., Hayashi Y., Yaffe M.B. (2009). Functional dynamics of Polo-like kinase 1 at the centrosome. Mol. Cell. Biol..

[53] Kim J., Lee K., Rhee K. (2015). PLK1 regulation of PCNT cleavage ensures fidelity of centriole separation during mitotic exit. Nat. Commun..

[54] Tsou M.-F.B., Wang W.-J., George K.A., Uryu K., Stearns T., Jallepalli P.V. (2009). Polo kinase and separase regulate the mitotic licensing of centriole duplication in human cells. Dev. Cell.

[55] Lončarek J., Hergert P., Khodjakov A. (2010). Centriole Reduplication during Prolonged Interphase Requires Procentriole Maturation Governed by Plk1. Curr. Biol..

[56] Zyss D., Gergely F. (2009). Centrosome function in cancer: Guilty or innocent?. Trends Cell Biol..

[57] Yim H. (2013). Current clinical trials with polo-like kinase 1 inhibitors in solid tumors. Anticancer Drugs.

[58] Li J., Hong M.J., Chow J.P.H., Man W.Y., Mak J.P.Y., Ma H.T., Poon R.Y.C. (2015). Co-inhibition of polo-like kinase 1 and Aurora kinases promotes mitotic catastrophe. Oncotarget.

[59] Godinho S.A., Pellman D. (2014). Causes and consequences of centrosome abnormalities in cancer. Philos. Trans. R. Soc. Lond. B Biol. Sci..

[60] Chen Z., Indjeian V.B., McManus M., Wang L., Dynlacht B.D. (2002). CP110, a cell cycle-dependent CDK substrate, regulates centrosome duplication in human cells. Dev. Cell.

[61] Bourke E., Brown J.A.L., Takeda S., Hochegger H., Morrison C.G. (2010). DNA damage induces Chk1-dependent threonine-160 phosphorylation and activation of Cdk2. Oncogene.

[62] Korzeniewski N., Hohenfellner M., Duensing S. (2013). The centrosome as potential target for cancer therapy and prevention. Expert Opin. Ther. Targets.

[63] Duensing A., Ghanem L., Steinman R.A., Liu Y., Duensing S. (2006). p21(Waf1/Cip1) deficiency stimulates centriole overduplication. Cell Cycle.

[64] Tetsu O., McCormick F. (2003). Proliferation of cancer cells despite CDK2 inhibition. Cancer Cell.

[65] Lee M., Seo M.Y., Chang J., Hwang D.S., Rhee K. (2017). PLK4 phosphorylation of CP110 is required for efficient centriole assembly. Cell Cycle.

[66] Holland A.J., Cleveland D.W. (2014). Polo-like kinase 4 inhibition: A strategy for cancer therapy?. Cancer Cell.

[67] Kuriyama R., Bettencourt-Dias M., Hoffmann I., Arnold M., Sandvig L. (2009). Gamma-tubulin-containing abnormal centrioles are induced by insufficient Plk4 in human HCT116 colorectal cancer cells. J. Cell Sci..

[68] Sillibourne J.E., Bornens M. (2010). Polo-like kinase 4: The odd one out of the family. Cell Div..

[69] Meraldi P., Honda R., Nigg E.A. (2002). Aurora-A overexpression reveals tetraploidization as a major route to centrosome amplification in p53-/- cells. EMBO J..

[70] Zhu J., Abbruzzese J.L., Izzo J., Hittelman W.N., Li D. (2005). AURKA amplification, chromosome instability, and centrosome abnormality in human pancreatic carcinoma cells. Cancer Genet. Cytogenet..

[71] Conroy P.C., Saladino C., Dantas T.J., Lalor P., Dockery P., Morrison C.G. (2012). C-NAP1 and rootletin restrain DNA damage-induced centriole splitting and facilitate ciliogenesis. Cell Cycle.

[72] Fang Y., Zhang X. (2016). Targeting NEK2 as a promising therapeutic approach for cancer treatment. Cell Cycle.

[73] Velimezi G., Liontos M., Vougas K., Roumeliotis T., Bartkova J., Sideridou M., Dereli-Oz A., Kocylowski M., Pateras I.S., Evangelou K. (2013). Functional interplay between the DNA-damage-response kinase ATM and ARF tumour suppressor protein in human cancer. Nat. Cell Biol..

[74] Manchado E., Guillamot M., Cárcer G., Eguren M., Trickey M., García-Higuera I., Moreno S., Yamano H., Cañamero M., Malumbres M. (2010). Targeting mitotic exit leads to tumor regression in vivo: Modulation by Cdk1, Mastl, and the PP2A/B55α,δ phosphatase. Cancer Cell.

[75] McCloy R.A., Rogers S., Caldon C.E., Lorca T., Castro A., Burgess A. (2014). Partial inhibition of Cdk1 in G_2_ phase overrides the SAC and decouples mitotic events. Cell Cycle.

[76] Chavali P.L., Chandrasekaran G., Barr A.R., Tátrai P., Taylor C., Papachristou E.K., Woods C.G., Chavali S., Gergely F. (2016). A CEP215-HSET complex links centrosomes with spindle poles and drives centrosome clustering in cancer. Nat. Commun..

[77] Sarli V., Huemmer S., Sunder-Plassmann N., Mayer T.U., Giannis A. (2005). Synthesis and Biological Evaluation of Novel Eg5 Inhibitors. ChemBioChem.

[78] Bourke E., Dodson H., Merdes A., Cuffe L., Zachos G., Walker M., Gillespie D., Morrison C.G. (2007). DNA damage induces Chk1-dependent centrosome amplification. EMBO Rep..

[79] Saladino C., Bourke E., Morrison C.G., Schatten H. (2012). The Centrosome.

[80] Brown J.A.L., Bourke E., Liptrot C., Dockery P., Morrison C.G. (2010). MCPH1/BRIT1 limits ionizing radiation-induced centrosome amplification. Oncogene.

[81] Saladino C., Bourke E., Conroy P.C., Morrison C.G. (2009). Centriole separation in DNA damage-induced centrosome amplification. Environ. Mol. Mutagen..

[82] Dodson H., Bourke E., Jeffers L.J., Vagnarelli P., Sonoda E., Takeda S., Earnshaw W.C., Merdes A., Morrison C. (2004). Centrosome amplification induced by DNA damage occurs during a prolonged G2 phase and involves ATM. EMBO J..

[83] Hollander M.C., Fornace A.J. (2002). Genomic instability, centrosome amplification, cell cycle checkpoints and Gadd45a. Oncogene.

[84] Bode A.M., Dong Z. (2007). The enigmatic effects of caffeine in cell cycle and cancer. Cancer Lett..

[85] Gatei M., Sloper K., Sörensen C., Syljuäsen R., Falck J., Hobson K., Savage K., Lukas J., Zhou B.-B., Bartek J. (2003). Ataxia-telangiectasia-mutated (ATM) and NBS1-dependent phosphorylation of Chk1 on Ser-317 in response to ionizing radiation. J. Biol. Chem..

[86] Guo Z., Kumagai A., Wang S.X., Dunphy W.G. (2000). Requirement for Atr in phosphorylation of Chk1 and cell cycle regulation in response to DNA replication blocks and UV-damaged DNA in Xenopus egg extracts. Genes Dev..

[87] Kanai M., Tong W.-M., Sugihara E., Wang Z.-Q., Fukasawa K., Miwa M. (2003). Involvement of poly(ADP-Ribose) polymerase 1 and poly(ADP-Ribosyl)ation in regulation of centrosome function. Mol. Cell. Biol..

[88] Tentori L., Leonetti C., Scarsella M., d’Amati G., Portarena I., Zupi G., Bonmassar E., Graziani G. (2002). Combined treatment with temozolomide and poly(ADP-ribose) polymerase inhibitor enhances survival of mice bearing hematologic malignancy at the central nervous system site. Blood.

[89] Lesueur P., Chevalier F., Austry J.-B., Waissi W., Burckel H., Noël G., Habrand J.-L., Saintigny Y., Joly F. (2017). Poly-(ADP-ribose)-polymerase inhibitors as radiosensitizers: A systematic review of pre-clinical and clinical human studies. Oncotarget.

[90] De Soto J.A., Wang X., Tominaga Y., Wang R.-H., Cao L., Qiao W., Li C., Xu X., Skoumbourdis A.P., Prindiville S.A. (2006). The inhibition and treatment of breast cancer with poly (ADP-ribose) polymerase (PARP-1) inhibitors. Int. J. Biol. Sci..

[91] Tsuda M., Tanaka M., Mushiake M., Takahashi J., Tanaka K., Watase J., Fujisawa J.-I., Miwa M. (2012). Novel pathway of centrosome amplification that does not require DNA lesions. Cancer Sci..

[92] Zhao B., Bower M.J., McDevitt P.J., Zhao H., Davis S.T., Johanson K.O., Green S.M., Concha N.O., Zhou B.-B.S. (2002). Structural basis for Chk1 inhibition by UCN-01. J. Biol. Chem..

[93] Bridges K.A., Chen X., Liu H., Rock C., Buchholz T.A., Shumway S.D., Skinner H.D., Meyn R.E. (2016). MK-8776, a novel chk1 kinase inhibitor, radiosensitizes p53-defective human tumor cells. Oncotarget.

[94] Yuan T.L., Cantley L.C. (2008). PI3K pathway alterations in cancer: Variations on a theme. Oncogene.

[95] Sarker D., Ang J.E., Baird R., Kristeleit R., Shah K., Moreno V., Clarke P.A., Raynaud F.I., Levy G., Ware J.A. (2015). First-in-human phase I study of pictilisib (GDC-0941), a potent pan-class I phosphatidylinositol-3-kinase (PI3K) inhibitor, in patients with advanced solid tumors. Clin. Cancer Res..

[96] Berenjeno I.M., Piñeiro R., Castillo S.D., Pearce W., McGranahan N., Dewhurst S.M., Meniel V., Birkbak N.J., Lau E., Sansregret L. (2017). Oncogenic PIK3CA induces centrosome amplification and tolerance to genome doubling. Nat. Commun..

[97] Jamieson S., Flanagan J.U., Kolekar S., Buchanan C., Kendall J.D., Lee W.-J., Rewcastle G.W., Denny W.A., Singh R., Dickson J. (2011). A drug targeting only p110α can block phosphoinositide 3-kinase signalling and tumour growth in certain cell types. Biochem. J..

[98] Yuan J., Mehta P.P., Yin M.-J., Sun S., Zou A., Chen J., Rafidi K., Feng Z., Nickel J., Engebretsen J. (2011). PF-04691502, a potent and selective oral inhibitor of PI3K and mTOR kinases with antitumor activity. Mol. Cancer Ther..

[99] Wei L., Surma M., Shi S., Lambert-Cheatham N., Shi J. (2016). Novel Insights into the Roles of Rho Kinase in Cancer. Arch. Immunol. Ther. Exp. (Warsz.).

[100] Nam H.-J., Chae S., Jang S.-H., Cho H., Lee J.-H. (2010). The PI3K-Akt mediates oncogenic Met-induced centrosome amplification and chromosome instability. Carcinogenesis.

[101] Samuels Y., Wang Z., Bardelli A., Silliman N., Ptak J., Szabo S., Yan H., Gazdar A., Powell S.M., Riggins G.J. (2004). High frequency of mutations of the PIK3CA gene in human cancers. Science.

[102] Miller T.W. (2012). Initiating breast cancer by PIK3CA mutation. Breast Cancer Res. BCR.

[103] Nigg E.A. (2002). Centrosome aberrations: Cause or consequence of cancer progression?. Nat. Rev. Cancer.

[104] Coelho P.A., Bury L., Shahbazi M.N., Liakath-Ali K., Tate P.H., Wormald S., Hindley C.J., Huch M., Archer J., Skarnes W.C. (2015). Over-expression of Plk4 induces centrosome amplification, loss of primary cilia and associated tissue hyperplasia in the mouse. Open Biol..

[105] Cunha-Ferreira I., Bento I., Pimenta-Marques A., Jana S.C., Lince-Faria M., Duarte P., Borrego-Pinto J., Gilberto S., Amado T., Brito D. (2013). Regulation of Autophosphorylation Controls PLK4 Self-Destruction and Centriole Number. Curr. Biol. CB.

[106] Rodrigues-Martins A., Riparbelli M., Callaini G., Glover D.M., Bettencourt-Dias M. (2007). Revisiting the role of the mother centriole in centriole biogenesis. Science.

[107] Rogers G.C., Rusan N.M., Roberts D.M., Peifer M., Rogers S.L. (2009). The SCF Slimb ubiquitin ligase regulates Plk4/Sak levels to block centriole reduplication. J. Cell Biol..

[108] Bettencourt-Dias M., Hildebrandt F., Pellman D., Woods G., Godinho S.A. (2011). Centrosomes and cilia in human disease. Trends Genet..

[109] Lohse I., Mason J., Mary P.C., Pintilie M., Bray M., Hedley D.W. (2017). Activity of the novel polo-like kinase 4 inhibitor CFI-400945 in pancreatic cancer patient-derived xenografts. Oncotarget.

[110] Sredni S.T., Bailey A.W., Suri A., Hashizume R., He X., Louis N., Gokirmak T., Piper D.R., Watterson D.M., Tomita1 T. (2017). Inhibition of polo-like kinase 4 (PLK4): A new therapeutic option for rhabdoid tumors and pediatric medulloblastoma. Oncotarget.

[111] Metge B., Ofori-Acquah S., Stevens T., Balczon R. (2004). Stat3 activity is required for centrosome duplication in Chinese hamster ovary cells. J. Biol. Chem..

[112] Morris E.J., Dedhar S. (2017). Stat3 in mitosis: A new role in clustering excess centrosomes. Cell Cycle.

[113] Morris E.J., Kawamura E., Gillespie J.A., Balgi A., Kannan N., Muller W.J., Roberge M., Dedhar S. (2017). Stat3 regulates centrosome clustering in cancer cells via Stathmin/PLK1. Nat. Commun..

[114] Avalle L., Pensa S., Regis G., Novelli F., Poli V. (2012). STAT1 and STAT3 in tumorigenesis: A matter of balance. JAKSTAT.

[115] Kushner E.J., Ferro L.S., Liu J.Y., Durrant J.R., Rogers S.L., Dudley A.C., Bautch V.L. (2014). Excess centrosomes disrupt endothelial cell migration via centrosome scattering. J. Cell Biol..

[116] Qiao X., Zhang L., Gamper A.M., Fujita T., Wan Y. (2010). APC/C-Cdh1: From cell cycle to cellular differentiation and genomic integrity. Cell Cycle.

[117] Hatano T., Sluder G. (2012). The interrelationship between APC/C and Plk1 activities in centriole disengagement. Biol. Open.

[118] Zhou Z., He M., Shah A.A., Wan Y. (2016). Insights into APC/C: From cellular function to diseases and therapeutics. Cell Div..

[119] Drosopoulos K., Tang C., Chao W.C.H., Linardopoulos S. (2014). APC/C is an essential regulator of centrosome clustering. Nat. Commun..

[120] Sackton K.L., Dimova N., Zeng X., Tian W., Zhang M., Sackton T.B., Meaders J., Pfaff K.L., Sigoillot F., Yu H. (2014). Synergistic blockade of mitotic exit by two chemical inhibitors of the APC/C. Nature.

[121] Wang L., Zhang J., Wan L., Zhou X., Wang Z., Wei W. (2015). Targeting Cdc20 as a novel cancer therapeutic strategy. Pharmacol. Ther..

[122] Wang C.Y., Huang E.Y.-H., Huang S.-C., Chung B.-C. (2015). DNA-PK/Chk2 induces centrosome amplification during prolonged replication stress. Oncogene.

[123] Watts C.A., Watts C.A., Richards F.M., Bender A., Bond P.J., Korb O., Kern O., Riddick M., Owen P., Myers R.M., Raff J. (2013). Design, synthesis, and biological evaluation of an allosteric inhibitor of HSET that targets cancer cells with supernumerary centrosomes. Chem. Biol..

[124] Castiel A., Visochek L., Mittelman L., Dantzer F., Izraeli S., Cohen-Armon M. (2011). A phenanthrene derived PARP inhibitor is an extra-centrosomes de-clustering agent exclusively eradicating human cancer cells. BMC Cancer.

[125] Xiao Y.-X., Yang W.-X. (2016). KIFC1: A promising chemotherapy target for cancer treatment?. Oncotarget.

[126] Rønnest M.H., Rebacz B., Markworth L., Terp A., Larsen T., Krämer A., Clausen M. (2009). Synthesis and structure-activity relationship of griseofulvin analogues as inhibitors of centrosomal clustering in cancer cells. J. Med. Chem..

[127] Kim Y., Alpmann P., Blaum-Feder S., Krämer S., Endo T., Lu D., Carson D., Schmidt-Wolf I.G.H. (2011). In vivo efficacy of griseofulvin against multiple myeloma. Leuk. Res..

[128] Raab M.S., Breitkreutz I., Anderhub S., Rønnest M.H., Leber B., Larsen T.O., Weiz L., Konotop G., Hayden P.J., Podar K. (2012). GF-15, a novel inhibitor of centrosomal clustering, suppresses tumor cell growth in vitro and in vivo. Cancer Res..

[129] Fielding A.B., Lim S., Montgomery K., Dobreva I., Dedhar S. (2011). A critical role of integrin-linked kinase, ch-TOG and TACC3 in centrosome clustering in cancer cells. Oncogene.

[130] Johannes J.W., Almeida L., Daly K., Ferguson A.D., Grosskurth S.E., Guan H., Howard T., Ioannidis S., Kazmirski S., Lamb M.L. (2015). Discovery of AZ0108, an orally bioavailable phthalazinone PARP inhibitor that blocks centrosome clustering. Bioorg. Med. Chem. Lett..

[131] Huang G.T.-J., Gronthos S., Shi S. (2009). Mesenchymal stem cells derived from dental tissues vs. those from other sources: Their biology and role in regenerative medicine. J. Dent. Res..

[132] Visochek L., Castiel A., Mittelman L., Elkin M., Atias D., Golan T., Izraeli S., Peretz T., Cohen-Armon M. (2017). Exclusive destruction of mitotic spindles in human cancer cells. Oncotarget.

[133] Murai J., Zhang Y., Morris J., Ji J., Takeda S., Doroshow J.H., Pommier Y. (2014). Rationale for poly(ADP-ribose) polymerase (PARP) inhibitors in combination therapy with camptothecins or temozolomide based on PARP trapping versus catalytic inhibition. J. Pharmacol. Exp. Ther..

[134] Liu X., Shi Y., Maag D.X., Palma J.P., Patterson M.J., Ellis P.A., Surber B.W., Ready D.B., Soni N.B., Ladror U.S. (2012). Iniparib nonselectively modifies cysteine-containing proteins in tumor cells and is not a bona fide PARP inhibitor. Clin. Cancer Res..

[135] Ekblad T., Camaioni E., Schüler H., Macchiarulo A. (2013). PARP inhibitors: Polypharmacology versus selective inhibition. FEBS J..

[136] Kalra J., Warburton C., Fang K., Edwards L., Daynard T., Waterhouse D., Dragowska W., Sutherland B.W., Dedhar S., Gelmon K. (2009). QLT0267, a small molecule inhibitor targeting integrin-linked kinase (ILK), and docetaxel can combine to produce synergistic interactions linked to enhanced cytotoxicity, reductions in P-AKT levels, altered F-actin architecture and improved treatment outcomes in an orthotopic breast cancer model. Breast Cancer Res. BCR.

[137] Sampson J., O’Regan L., Dyer M.J.S., Bayliss R., Fry A.M. (2017). Hsp72 and Nek6 Cooperate to Cluster Amplified Centrosomes in Cancer Cells. Cancer Res..

[138] Massey A.J., Williamson D.S., Browne H., Murray J.B., Dokurno P., Shaw T., Macias A.T., Daniels Z., Geoffroy S., Dopson M. (2010). A novel, small molecule inhibitor of Hsc70/Hsp70 potentiates Hsp90 inhibitor induced apoptosis in HCT116 colon carcinoma cells. Cancer Chemother. Pharmacol..

[139] Kawamura E., Fielding A.B., Kannan N., Balgi A., Eaves C.J., Roberge M., Dedhar S. (2013). Identification of novel small molecule inhibitors of centrosome clustering in cancer cells. Oncotarget.

[140] Karna P., Rida P.C., Pannu V., Gupta K.K., Dalton W.B., Joshi H., Yang V.W., Zhou J., Aneja R. (2011). A novel microtubule-modulating noscapinoid triggers apoptosis by inducing spindle multipolarity via centrosome amplification and declustering. Cell Death Differ..

[141] Pannu V., Karna P., Sajja H.K., Shukla D., Aneja R. (2011). Synergistic antimicrotubule therapy for prostate cancer. Biochem. Pharmacol..

[142] Konotop G., Bausch E., Nagai T., Turchinovich A., Becker N., Benner A., Boutros M., Mizuno K., Krämer A., Raab M.S. (2016). Pharmacological Inhibition of Centrosome Clustering by Slingshot-Mediated Cofilin Activation and Actin Cortex Destabilization. Cancer Res..

[143] Xi Y., Chen M., Liu X., Lu Z., Ding Y., Li D. (2014). CP-673451, a platelet-derived growth-factor receptor inhibitor, suppresses lung cancer cell proliferation and migration. OTT.

[144] Rhys A.D., Monteiro P., Smith C., Vaghela M., Arnandis T., Kato T., Leitinger B., Sahai E., McAinsh A., Charras G. (2018). Loss of E-cadherin provides tolerance to centrosome amplification in epithelial cancer cells. J. Cell Biol..

[145] Mountain V., Simerly C., Howard L., Ando A., Schatten G., Compton D.A. (1999). The kinesin-related protein, HSET, opposes the activity of Eg5 and cross-links microtubules in the mammalian mitotic spindle. J. Cell Biol..

[146] She Z.-Y., Yang W.-X. (2017). Molecular mechanisms of kinesin-14 motors in spindle assembly and chromosome segregation. J. Cell Sci..

[147] Krämer A., Maier B., Bartek J. (2011). Centrosome clustering and chromosomal (in)stability: A matter of life and death. Mol. Oncol..

[148] Rebacz B., Larsen T.O., Clausen M.H., Rønnest M.H., Löffler H., Ho A.D., Krämer A. (2007). Identification of griseofulvin as an inhibitor of centrosomal clustering in a phenotype-based screen. Cancer Res..

[149] Pannu V., Rida P.C., Celik B., Turaga R.C., Ogden A., Cantuaria G., Gopalakrishnan J., Aneja R. (2014). Centrosome-declustering drugs mediate a two-pronged attack on interphase and mitosis in supercentrosomal cancer cells. Cell Death Dis..

[150] Huang S.-H., Xiong M., Chen X.P., Xiao Z.Y., Zhao Y.F., Huang Z.Y. (2008). PJ34, an inhibitor of PARP-1, suppresses cell growth and enhances the suppressive effects of cisplatin in liver cancer cells. Oncol. Rep..

[151] Donawho C.K., Luo Y., Luo Y.-P., Penning T.D., Bauch J.L., Bouska J.J., Bontcheva-Diaz V.D., Cox B.F., DeWeese T.L., Dillehay L.E. (2007). ABT-888, an orally active poly(ADP-ribose) polymerase inhibitor that potentiates DNA-damaging agents in preclinical tumor models. Clin. Cancer Res..

[152] O’Regan L., Sampson J., Richards M.W., Knebel A., Roth D., Hood F.E., Straube A., Royle S.J., Bayliss R., Fry A.M. (2015). Hsp72 is targeted to the mitotic spindle by Nek6 to promote K-fiber assembly and mitotic progression. J. Cell Biol..

[153] Lopus M., Naik P.K. (2015). Taking aim at a dynamic target: Noscapinoids as microtubule-targeted cancer therapeutics. Pharmacol. Rep..

[154] Hojjat-Farsangi M. (2014). Small-molecule inhibitors of the receptor tyrosine kinases: Promising tools for targeted cancer therapies. Int. J. Mol. Sci..

[155] Lord C.J., Ashworth A. (2012). The DNA damage response and cancer therapy. Nature.

[156] Hengel S.R., Spies M.A., Spies M. (2017). Small-Molecule Inhibitors Targeting DNA Repair and DNA Repair Deficiency in Research and Cancer Therapy. Cell Chem. Biol..

[157] Jeggo P.A., Pearl L.H., Carr A.M. (2016). DNA repair, genome stability and cancer: A historical perspective. Nat. Rev. Cancer.

[158] Ko H.L., Ren E.C. (2012). Functional Aspects of PARP1 in DNA Repair and Transcription. Biomolecules.

[159] Thompson L.H. (2012). Recognition, signaling, and repair of DNA double-strand breaks produced by ionizing radiation in mammalian cells: The molecular choreography. Mutat. Res..

[160] Ray Chaudhuri A., Nussenzweig A. (2017). The multifaceted roles of PARP1 in DNA repair and chromatin remodelling. Nat. Rev. Mol. Cell Biol..

[161] Pauli C., Hopkins B.D., Prandi D., Shaw R., Fedrizzi T., Sboner A., Sailer V., Augello M., Puca L., Rosati R. (2017). Personalized In Vitro and In Vivo Cancer Models to Guide Precision Medicine. Cancer Discov..

[162] Bhargava R., Onyango D.O., Stark J.M. (2016). Regulation of Single-Strand Annealing and its Role in Genome Maintenance. Trends Genet..

[163] Frit P., Barboule N., Yuan Y., Gomez D., Calsou P. (2014). Alternative end-joining pathway(s): Bricolage at DNA breaks. DNA Repair (Amst.).

[164] McVey M., Lee S.E. (2008). MMEJ repair of double-strand breaks (director’s cut): Deleted sequences and alternative endings. Trends Genet..

[165] Howard S.M., Yanez D.A., Stark J.M. (2015). DNA damage response factors from diverse pathways, including DNA crosslink repair, mediate alternative end joining. PLoS Genet..

[166] Chernikova S.B., Game J.C., Brown J.M. (2012). Inhibiting homologous recombination for cancer therapy. Cancer Biol. Ther..

[167] Truong L.N., Li Y., Shi L.Z., Hwang P.Y., He J., Wang H., Razavian N., Berns M.W., Wu X. (2013). Microhomology-mediated End Joining and Homologous Recombination share the initial end resection step to repair DNA double-strand breaks in mammalian cells. Proc. Natl. Acad. Sci. USA.

[168] Sousa F.G., Matuo R., Soares D.G., Escargueil A.E., Henriques J.A., Larsen A.K., Saffi J. (2012). PARPs and the DNA damage response. Carcinogenesis.

[169] Krietsch J., Caron M.-C., Gagné J.-P., Ethier C., Vignard J., Vincent M., Rouleau M., Hendzel M.J., Poirier G.G., Masson J.-Y. (2012). PARP activation regulates the RNA-binding protein NONO in the DNA damage response to DNA double-strand breaks. Nucleic Acids Res..

[170] Hickson I., Zhao Y., Richardson C.J., Green S.J., Martin N.M., Orr A.I., Reaper P.M., Jackson S.P., Curtin N.J., Smith G.C. (2004). Identification and characterization of a novel and specific inhibitor of the ataxia-telangiectasia mutated kinase ATM. Cancer Res..

[171] Haince J.-F., Kozlov S., Dawson V.L., Dawson T.M., Hendzel M.J., Lavin M.F., Poirier G.G. (2007). Ataxia telangiectasia mutated (ATM) signaling network is modulated by a novel poly(ADP-ribose)-dependent pathway in the early response to DNA-damaging agents. J. Biol. Chem..

[172] Farmer H., McCabe N., Lord C.J., Tutt A.N., Johnson D.A., Richardson T.B., Santarosa M., Dillon K.J., Hickson I., Knights C. (2005). Targeting the DNA repair defect in BRCA mutant cells as a therapeutic strategy. Nature.

[173] Murata S., Zhang C., Finch N., Zhang K., Campo L., Breuer E.K. (2016). Predictors and Modulators of Synthetic Lethality: An Update on PARP Inhibitors and Personalized Medicine. Biomed. Res. Int..

[174] Sonnenblick A., de Azambuja E., Azim H.A., Piccart M. (2015). An update on PARP inhibitors—Moving to the adjuvant setting. Nat. Rev. Clin. Oncol..

[175] Lord C.J., Ashworth A. (2017). PARP inhibitors: Synthetic lethality in the clinic. Science.

[176] Murai J., Huang S.Y., Renaud A., Zhang Y., Ji J., Takeda S., Morris J., Teicher B., Doroshow J.H., Pommier Y. (2014). Stereospecific PARP trapping by BMN 673 and comparison with olaparib and rucaparib. Mol. Cancer Ther..

[177] Pommier Y., O’Connor M.J., de Bono J. (2016). Laying a trap to kill cancer cells: PARP inhibitors and their mechanisms of action. Sci. Transl. Med..

[178] Edwards S.L., Brough R., Lord C.J., Natrajan R., Vatcheva R., Levine D.A., Boyd J., Reis-Filho J.S., Ashworth A. (2008). Resistance to therapy caused by intragenic deletion in BRCA2. Nature.

[179] Plummer R., Stephens P., Aissat-Daudigny L., Cambois A., Moachon G., Brown P.D., Campone M. (2014). Phase 1 dose-escalation study of the PARP inhibitor CEP-9722 as monotherapy or in combination with temozolomide in patients with solid tumors. Cancer Chemother. Pharmacol..

[180] Incorvaia L., Passiglia F., Rizzo S., Galvano A., Listì A., Barraco N., Maragliano R., Calò V., Natoli C., Ciaccio M. (2017). ‘Back to a false normality’: New intriguing mechanisms of resistance to PARP inhibitors. Oncotarget.

[181] Liu Y., Burness M.L., Martin-Trevino R., Guy J., Bai S., Harouaka R., Brooks M.D., Shang L., Fox A., Luther T.K. (2017). RAD51 Mediates Resistance of Cancer Stem Cells to PARP Inhibition in Triple-Negative Breast Cancer. Clin. Cancer Res..

[182] Smith J., Tho L.M., Xu N., Gillespie D.A. (2010). The ATM-Chk2 and ATR-Chk1 pathways in DNA damage signaling and cancer. Adv. Cancer Res..

[183] Domchek S.M. (2017). Reversion Mutations with Clinical Use of PARP Inhibitors: Many Genes, Many Versions. Cancer Discov..

[184] Golding S.E., Rosenberg E., Valerie N., Hussaini I., Frigerio M., Cockcroft X.F., Chong W.Y., Hummersone M., Rigoreau L., Menear K.A. (2009). Improved ATM kinase inhibitor KU-60019 radiosensitizes glioma cells, compromises insulin, AKT and ERK prosurvival signaling, and inhibits migration and invasion. Mol. Cancer Ther..

[185] Velic D., Couturier A.M., Ferreira M.T., Rodrigue A., Poirier G.G., Fleury F., Masson J.Y. (2015). DNA Damage Signalling and Repair Inhibitors: The Long-Sought-After Achilles’ Heel of Cancer. Biomolecules.

[186] Degorce S.L., Barlaam B., Cadogan E., Dishington A., Ducray R., Glossop S.C., Hassall L.A., Lach F., Lau A., McGuire T.M. (2016). Discovery of Novel 3-Quinoline Carboxamides as Potent, Selective, and Orally Bioavailable Inhibitors of Ataxia Telangiectasia Mutated (ATM) Kinase. J. Med. Chem..

[187] Fokas E., Prevo R., Pollard J.R., Reaper P.M., Charlton P.A., Cornelissen B., Vallis K.A., Hammond E.M., Olcina M.M., Gillies M.W. (2012). Targeting ATR in vivo using the novel inhibitor VE-822 results in selective sensitization of pancreatic tumors to radiation. Cell Death Dis..

[188] Weber A.M., Ryan A.J. (2015). ATM and ATR as therapeutic targets in cancer. Pharmacol. Ther..

[189] Wengner A.M., Siemeister G., Luecking U., Lefranc J., Lienau P., Deeg G., Lagkadinou E., Liu Li., Golfier S., Schatz C. (2017). Abstract 836: ATR inhibitor BAY 1895344 shows potent anti-tumor efficacy in monotherapy and strong combination potential with the targeted alpha therapy Radium-223 dichloride in preclinical tumor models. Cancer Res..

[190] Blackford A.N., Jackson S.P. (2017). ATM, ATR, and DNA-PK: The Trinity at the Heart of the DNA Damage Response. Mol. Cell.

[191] Lavin M.F. (2008). Ataxia-telangiectasia: From a rare disorder to a paradigm for cell signalling and cancer. Nat. Rev. Mol. Cell Biol..

[192] Shiloh Y. (2003). ATM and related protein kinases: Safeguarding genome integrity. Nat. Rev. Cancer.

[193] Zhu Y., Mao C., Wu J., Li S., Ma R., Cao H., Ji M., Jing C., Tang J. (2014). Improved ataxia telangiectasia mutated kinase inhibitor KU60019 provides a promising treatment strategy for non-invasive breast cancer. Oncol. Lett..

[194] Barlaam B., Pike K. (2016). Identifying high quality, potent and selective inhibitors of ATM kinase: Discovery of AZD0156. Eur. J. Cancer.

[195] Brandsma I., Fleuren E.D.G., Williamson C.T., Lord C.J. (2017). Directing the use of DDR kinase inhibitors in cancer treatment. Expert Opin. Investig. Drugs.

[196] Rainey M.D., Charlton M.E., Stanton R.V., Kastan M.B. (2008). Transient inhibition of ATM kinase is sufficient to enhance cellular sensitivity to ionizing radiation. Cancer Res..

[197] Morgado-Palacin I., Day A., Murga M., Lafarga V., Anton M.E., Tubbs A., Chen H.T., Ergan A., Anderson R., Bhandoola A. (2016). Targeting the kinase activities of ATR and ATM exhibits antitumoral activity in mouse models of MLL-rearranged AML. Sci. Signal..

[198] Hall A.B., Newsome D., Wang Y., Boucher D.M., Eustace B., Gu Y., Hare B., Johnson M.A., Milton S., Murphy C.E. (2014). Potentiation of tumor responses to DNA damaging therapy by the selective ATR inhibitor VX-970. Oncotarget.

[199] Nishida H., Tatewaki N., Nakajima Y., Magara T., Ko K.M., Hamamori Y., Konishi T. (2009). Inhibition of ATR protein kinase activity by schisandrin B in DNA damage response. Nucleic Acids Res..

[200] Foote K.M., Blades K., Cronin A., Fillery S., Guichard S.S., Hassall L., Hickson I., Jacq X., Jewsbury P.J., McGuire T.M. (2013). Discovery of 4-{4-[(3*R*)-3-Methylmorpholin-4-yl]-6-[1-(methylsulfonyl)cyclopropyl]pyrimidin-2-yl}-1H-indole (AZ20): A potent and selective inhibitor of ATR protein kinase with monotherapy in vivo antitumor activity. J. Med. Chem..

[201] Veith S., Mangerich A. (2015). RecQ helicases and PARP1 team up in maintaining genome integrity. Ageing Res. Rev..

[202] Foote K.M., Lau A., Nissink J.W.M. (2015). Drugging ATR: Progress in the development of specific inhibitors for the treatment of cancer. Future Med. Chem..

[203] Kwok M., Davies N., Agathanggelou A., Smith E., Petermann E., Yates E., Brown J., Lau A., Stankovic T. (2015). Synthetic lethality in chronic lymphocytic leukaemia with DNA damage response defects by targeting the ATR pathway. Lancet Oncol..

[204] Peasland A., Wang L.Z., Rowling E., Kyle S., Chen T., Hopkins A., Cliby W.A., Sarkaria J., Beale G., Edmondson R.J. (2011). Identification and evaluation of a potent novel ATR inhibitor, NU6027, in breast and ovarian cancer cell lines. Br. J. Cancer.

[205] Brosh R.M. (2013). DNA helicases involved in DNA repair and their roles in cancer. Nat. Rev. Cancer.

[206] Bernstein K.A., Gangloff S., Rothstein R. (2010). The RecQ DNA helicases in DNA repair. Annu. Rev. Genet..

[207] Ellis N.A., Groden J., Ye T.Z., Straughen J., Lennon D.J., Ciocci S., Proytcheva M., German J. (1995). The Bloom’s syndrome gene product is homologous to RecQ helicases. Cell.

[208] Arora H., Chacon A.H., Choudhary S., McLeod M.P., Meshkov L., Nouri K., Izakovic J. (2014). Bloom syndrome. Int. J. Dermatol..

[209] Yu C.E., Oshima J., Fu Y.H., Wijsman E.M., Hisama F., Alisch R., Matthews S., Nakura J., Miki T., Ouais S. (1996). Positional cloning of the Werner’s syndrome gene. Science.

[210] Kitao S., Shimamoto A., Goto M., Miller R.W., Smithson W.A., Lindor N.M., Furuichi Y. (1999). Mutations in RECQL4 cause a subset of cases of Rothmund-Thomson syndrome. Nat. Genet..

[211] Mo D., Zhao Y., Balajee A.S. (2018). Human RecQL4 helicase plays multifaceted roles in the genomic stability of normal and cancer cells. Cancer Lett..

[212] Croteau D.L., Popuri V., Opresko P.L., Bohr V.A. (2014). Human RecQ helicases in DNA repair, recombination, and replication. Ann. Rev. Biochem..

[213] Ramamoorthy M., Tadokoro T., Rybanska I., Ghosh A.K., Wersto R., May A., Kulikowicz T., Sykora P., Croteau D.L., Bohr V.A. (2012). RECQL5 cooperates with Topoisomerase II alpha in DNA decatenation and cell cycle progression. Nucleic Acids Res..

[214] Hu Y., Raynard S., Sehorn M.G., Lu X., Bussen W., Zheng L., Stark J.M., Barnes E.L., Chi P., Janscak P. (2007). RECQL5/Recql5 helicase regulates homologous recombination and suppresses tumor formation via disruption of Rad51 presynaptic filaments. Genes Dev..

[215] Mankouri H.W., Hickson I.D. (2007). The RecQ helicase-topoisomerase III-Rmi1 complex: A DNA structure-specific ‘dissolvasome’?. Trends Biochem. Sci..

[216] Manthei K.A., Keck J.L. (2013). The BLM dissolvasome in DNA replication and repair. Cell. Mol. Life Sci..

[217] Tripathi V., Agarwal H., Priya S., Batra H., Modi P., Pandey M., Saha D., Raghavan S.C., Sengupta S. (2018). MRN complex-dependent recruitment of ubiquitylated BLM helicase to DSBs negatively regulates DNA repair pathways. Nat. Commun..

[218] Nguyen G.H., Dexheimer T.S., Rosenthal A.S., Chu W.K., Singh D.K., Mosedale G., Bachrati C.Z., Schultz L., Sakurai M., Savitsky P. (2013). A small molecule inhibitor of the BLM helicase modulates chromosome stability in human cells. Chem. Biol..

[219] Aggarwal M., Banerjee T., Sommers J.A., Brosh R.M. (2013). Targeting an Achilles’ heel of cancer with a WRN helicase inhibitor. Cell Cycle.

[220] Moles R., Bai X.T., Chaib-Mezrag H., Nicot C. (2016). WRN-targeted therapy using inhibitors NSC 19630 and NSC 617145 induce apoptosis in HTLV-1-transformed adult T-cell leukemia cells. J. Hematol. Oncol..

[221] Aggarwal M., Sommers J.A., Shoemaker R.H., Brosh R.M. (2011). Inhibition of helicase activity by a small molecule impairs Werner syndrome helicase (WRN) function in the cellular response to DNA damage or replication stress. Proc. Natl. Acad. Sci. USA.

[222] Gilbert N., Allan J. (2014). Supercoiling in DNA and chromatin. Curr. Opin. Genet. Dev..

[223] Pommier Y. (2013). Drugging topoisomerases: Lessons and challenges. ACS Chem. Biol..

[224] Li F., Jiang T., Li Q., Ling X. (2017). Camptothecin (CPT) and its derivatives are known to target topoisomerase I (Top1) as their mechanism of action: Did we miss something in CPT analogue molecular targets for treating human disease such as cancer?. Am. J. Cancer Res..

[225] Liu L.F., Desai S.D., Li T.K., Mao Y., Sun M., Sim S.P. (2000). Mechanism of action of camptothecin. Ann. N. Y. Acad. Sci..

[226] Fox B.M., Xiao X., Antony S., Kohlhagen G., Pommier Y., Staker B.L., Stewart L., Cushman M. (2003). Design, synthesis, and biological evaluation of cytotoxic 11-alkenylindenoisoquinoline topoisomerase I inhibitors and indenoisoquinoline-camptothecin hybrids. J. Med. Chem..

[227] Mathijssen R.H.J., Loos W.J., Verweij J., Sparreboom A. (2002). Pharmacology of topoisomerase I inhibitors irinotecan (CPT-11) and topotecan. Curr. Cancer Drug Targets.

[228] Kurtzberg L.S., Roth S., Krumbholz R., Crawford J., Bormann C., Dunham S., Yao M., Rouleau C., Bagley R.G., Yu X.J. (2011). Genz-644282, a novel non-camptothecin topoisomerase I inhibitor for cancer treatment. Clin. Cancer Res..

[229] Schellens J.H., Maliepaard M., Scheper R.J., Scheffer G.L., Jonker J.W., Smit J.W., Beijnen J.H., Schinkel A.H. (2000). Transport of topoisomerase I inhibitors by the breast cancer resistance protein. Potential clinical implications. Ann. N. Y. Acad. Sci..

[230] Beretta G.L., Perego P., Zunino F. (2006). Mechanisms of cellular resistance to camptothecins. Curr. Med. Chem..

[231] Pommier Y., Cushman M. (2009). The indenoisoquinoline noncamptothecin topoisomerase I inhibitors: Update and perspectives. Mol. Cancer Ther..

[232] Antony S., Agama K.K., Miao Z.H., Takagi K., Wright M.H., Robles A.I., Varticovski L., Nagarajan M., Morrell A., Cushman M. (2007). Novel indenoisoquinolines NSC 725776 and NSC 724998 produce persistent topoisomerase I cleavage complexes and overcome multidrug resistance. Cancer Res..

[233] Cortazar P., Justice R., Johnson J., Sridhara R., Keegan P., Pazdur R. (2012). US Food and Drug Administration approval overview in metastatic breast cancer. J. Clin. Oncol..

[234] Shibata A., Moiani D., Arvai A.S., Perry J., Harding S.M., Genois M.M., Maity R., van Rossum-Fikkert S., Kertokalio A., Romoli F. (2014). DNA double-strand break repair pathway choice is directed by distinct MRE11 nuclease activities. Mol. Cell.

[235] McNeil E.M., Astell K.R., Ritchie A.M., Shave S., Houston D.R., Bakrania P., Jones H.M., Khurana P., Wallace C., Chapman T. (2015). Inhibition of the ERCC1-XPF structure-specific endonuclease to overcome cancer chemoresistance. DNA Repair (Amst.).

[236] Nitiss J.L. (2009). Targeting DNA topoisomerase II in cancer chemotherapy. Nat. Rev. Cancer.

[237] Wijdeven R.H., Pang B., van der Zanden S.Y., Qiao X., Blomen V., Hoogstraat M., Lips E.H., Janssen L., Wessels L., Brummelkamp T.R. (2015). Genome-Wide Identification and Characterization of Novel Factors Conferring Resistance to Topoisomerase II Poisons in Cancer. Cancer Res..

[238] Tacar O., Sriamornsak P., Dass C.R. (2013). Doxorubicin: An update on anticancer molecular action, toxicity and novel drug delivery systems. J. Pharm. Pharmacol..

[239] Khasraw M., Bell R., Dang C. (2012). Epirubicin: Is it like doxorubicin in breast cancer? A clinical review. Breast.

[240] Webb A., Cunningham D., Scarffe J.H., Harper P., Norman A., Joffe J.K., Hughes M., Mansi J., Findlay M., Hill A., Oates J. (1997). Randomized trial comparing epirubicin, cisplatin, and fluorouracil versus fluorouracil, doxorubicin, and methotrexate in advanced esophagogastric cancer. J. Clin. Oncol..

[241] Wagner A.D., Grothe W., Haerting J., Kleber G., Grothey A., Fleig W.E. (2006). Chemotherapy in advanced gastric cancer: A systematic review and meta-analysis based on aggregate data. J. Clin. Oncol..

[242] Hande K.R. (1998). Etoposide: Four decades of development of a topoisomerase II inhibitor. Eur. J. Cancer.

[243] Wu C.-C., Li T.K., Farh L., Lin L.Y., Lin T.S., Yu Y.J., Yen T.J., Chiang C.W., Chan N.L. (2011). Structural basis of type II topoisomerase inhibition by the anticancer drug etoposide. Science.

[244] Pommier Y., Capranico G., Orr A., Kohn K.W. (1991). Distribution of topoisomerase II cleavage sites in simian virus 40 DNA and the effects of drugs. J. Mol. Biol..

[245] Evison B.J., Sleebs B.E., Watson K.G., Phillips D.R., Cutts S.M. (2016). Mitoxantrone, More than Just Another Topoisomerase II Poison. Med. Res. Rev..

[246] Vavrova A., Jansova H., Mackova E., Machacek M., Haskova P., Tichotova L., Sterba M., Simunek T. (2013). Catalytic inhibitors of topoisomerase II differently modulate the toxicity of anthracyclines in cardiac and cancer cells. PLoS ONE.

[247] Williams R.S., Williams J.S., Tainer J.A. (2007). Mre11-Rad50-Nbs1 is a keystone complex connecting DNA repair machinery, double-strand break signaling, and the chromatin template. Biochem. Cell Biol..

[248] Stracker T.H., Petrini J.H.J. (2011). The MRE11 complex: Starting from the ends. Nat. Rev. Mol. Cell Biol..

[249] Dupré A., Boyer-Chatenet L., Sattler R.M., Modi A.P., Lee J.H., Nicolette M.L., Kopelovich L., Jasin M., Baer R., Paull T.T. (2008). A forward chemical genetic screen reveals an inhibitor of the Mre11-Rad50-Nbs1 complex. Nat. Chem. Biol..

[250] Rass E., Grabarz A., Plo I., Gautier J., Bertrand P., Lopez B.S. (2009). Role of Mre11 in chromosomal nonhomologous end joining in mammalian cells. Nat. Struct. Mol. Biol..

[251] Jordheim L.P., Barakat K.H., Heinrich-Balard L., Matera E.L., Cros-Perrial E., Bouledrak K., El Sabeh R., Perez-Pineiro R., Wishart D.S., Cohen R. (2013). Small molecule inhibitors of ERCC1-XPF protein-protein interaction synergize alkylating agents in cancer cells. Mol. Pharmacol..

[252] Srivastava M., Raghavan S.C. (2015). DNA double-strand break repair inhibitors as cancer therapeutics. Chem. Biol..

[253] Gavande N.S., VanderVere-Carozza P.S., Hinshaw H.D., Jalal S.I., Sears C.R., Pawelczak K.S., Turchi J.J. (2016). DNA repair targeted therapy: The past or future of cancer treatment?. Pharmacol. Ther..

[254] Jette N., Lees-Miller S.P. (2015). The DNA-dependent protein kinase: A multifunctional protein kinase with roles in DNA double strand break repair and mitosis. Prog. Biophys. Mol. Biol..

[255] Stiff T., O’Driscoll M., Rief N., Iwabuchi K., Löbrich M., Jeggo P.A. (2004). ATM and DNA-PK function redundantly to phosphorylate H2AX after exposure to ionizing radiation. Cancer Res..

[256] Ciszewski W.M., Tavecchio M., Dastych J., Curtin N.J. (2014). DNA-PK inhibition by NU7441 sensitizes breast cancer cells to ionizing radiation and doxorubicin. Breast Cancer Res. Treat..

[257] Zhou X., Zhang X., Xie Y., Tanaka K., Wang B., Zhang H. (2013). DNA-PKcs inhibition sensitizes cancer cells to carbon-ion irradiation via telomere capping disruption. PLoS ONE.

[258] Allen C., Halbrook J., Nickoloff J.A. (2003). Interactive competition between homologous recombination and non-homologous end joining. Mol. Cancer Res..

[259] Sarkaria J.N., Tibbetts R.S., Busby E.C., Kennedy A.P., Hill D.E., Abraham R.T. (1998). Inhibition of phosphoinositide 3-kinase related kinases by the radiosensitizing agent wortmannin. Cancer Res..

[260] Vlahos C.J., Matter W.F., Hui K.Y., Brown R.F. (1994). A specific inhibitor of phosphatidylinositol 3-kinase, 2-(4-morpholinyl)-8-phenyl-4*H*-1-benzopyran-4-one (LY294002). J. Biol. Chem..

[261] Collis S.J., DeWeese T.L., Jeggo P.A., Parker A.R. (2005). The life and death of DNA-PK. Oncogene.

[262] Leahy J.J., Golding B.T., Griffin RJ., Hardcastle I.R., Richardson C., Rigoreau L., Smith G.C. (2004). Identification of a highly potent and selective DNA-dependent protein kinase (DNA-PK) inhibitor (NU7441) by screening of chromenone libraries. Bioorg. Med. Chem. Lett..

[263] Zhao Y., Thomas H.D., Batey M.A., Cowell I.G., Richardson C.J., Griffin R.J., Calvert A.H., Newell D.R., Smith G.C., Curtin N.J. (2006). Preclinical evaluation of a potent novel DNA-dependent protein kinase inhibitor NU7441. Cancer Res..

[264] Willmore E., de Caux S., Sunter N.J., Tilby M.J., Jackson G.H., Austin C.A., Durkacz B.W. (2004). A novel DNA-dependent protein kinase inhibitor, NU7026, potentiates the cytotoxicity of topoisomerase II poisons used in the treatment of leukemia. Blood.

[265] Tavecchio M., Munck J.M., Cano C., Newell D.R., Curtin N.J. (2012). Further characterisation of the cellular activity of the DNA-PK inhibitor, NU7441, reveals potential cross-talk with homologous recombination. Cancer Chemother. Pharmacol..

[266] Zenke F.T., Zimmermann A., Sirrenberg C., Dahmen H., Vassilev L., Pehl U., Fuchss T., Blaukat A. (2016). M3814, a novel investigational DNA-PK inhibitor: Enhancing the effect of fractionated radiotherapy leading to complete regression of tumors in mice. Cancer Ref..

[267] Harnor S.J., Brennan A., Cano C. (2017). Targeting DNA-Dependent Protein Kinase for Cancer Therapy. ChemMedChem.

[268] Boucher D., Newsome D., Takemoto D., Hillier S., Wang Y., Arimoto R., Maxwell J., Charifson P., Fields S.Z., Tanner K., Penney M.S. (2017). Abstract P5-06-05: Preclinical characterization of VX-984, a selective DNA-dependent protein kinase (DNA-PK) inhibitor in combination with doxorubicin in breast and ovarian cancers. Cancer Res..

[269] Boucher D., Hillier S., Newsome D., Wang Y., Takemoto D., Gu Y., Markland W., Hoover R., Arimoto R., Maxwell J. (2016). Preclinical characterization of the selective DNA-dependent protein kinase (DNA-PK) inhibitor VX-984 in combination with chemotherapy. Ann. Oncol..

[270] Neal J.A., Dang V., Douglas P., Wold M.S., Lees-Miller S.P., Meek K. (2011). Inhibition of homologous recombination by DNA-dependent protein kinase requires kinase activity, is titratable, and is modulated by autophosphorylation. Mol. Cell. Biol..

[271] Srivastava M., Nambiar M., Sharma S., Karki S.S., Goldsmith G., Hegde M., Kumar S., Pandey M., Singh R.K., Ray P. (2012). An inhibitor of nonhomologous end-joining abrogates double-strand break repair and impedes cancer progression. Cell.

[272] Polo S.E., Jackson S.P. (2011). Dynamics of DNA damage response proteins at DNA breaks: A focus on protein modifications. Genes Dev..

[273] Riballo E., Critchlow S.E., Teo S.H., Doherty A.J., Priestley A., Broughton B., Kysela B., Beamish H., Plowman N., Arlett C.F. (1999). Identification of a defect in DNA ligase IV in a radiosensitive leukaemia patient. Curr. Biol. CB.

[274] Kondo N., Takahashi A., Mori E., Ohnishi K., McKinnon P.J., Sakaki T., Nakase H., Ohnishi T. (2009). DNA ligase IV as a new molecular target for temozolomide. Biochem. Biophys. Res. Commun..

[275] Greco G.E., Matsumoto Y., Brooks R.C., Lu Z., Lieber M.R., Tomkinson A.E. (2016). SCR7 is neither a selective nor a potent inhibitor of human DNA ligase IV. DNA Repair (Amst.).

[276] Thompson L.H., Schild D. (2001). Homologous recombinational repair of DNA ensures mammalian chromosome stability. Mutat. Res..

[277] Li X., Heyer W.-D. (2008). Homologous recombination in DNA repair and DNA damage tolerance. Cell Res..

[278] Zhang J., Dai Q., Park D., Deng X. (2016). Targeting DNA Replication Stress for Cancer Therapy. Genes (Basel).

[279] Helleday T., Petermann E., Lundin C., Hodgson B., Sharma R.A. (2008). DNA repair pathways as targets for cancer therapy. Nat. Rev. Cancer.

[280] Huang F., Mazina O.M., Zentner I.J., Cocklin S., Mazin A.V. (2012). Inhibition of homologous recombination in human cells by targeting RAD51 recombinase. J. Med. Chem..

[281] Budke B., Logan H.L., Kalin J.H., Zelivianskaia A.S., Cameron M.W., Miller L.L., Stark J.M., Kozikowski A.P., Bishop D.K., Connell P.P. (2012). RI-1: A chemical inhibitor of RAD51 that disrupts homologous recombination in human cells. Nucleic Acids Res..

[282] Jayathilaka K., Sheridan S.D., Bold T.D., Bochenska K., Logan H.L., Weichselbaum R.R., Bishop D.K., Connell P.P. (2008). A chemical compound that stimulates the human homologous recombination protein RAD51. Proc. Natl. Acad. Sci. USA.

[283] Zhu J., Chen H., Guo X.E., Qiu X.L., Hu C.M., Chamberlin A.R., Lee W.H. (2015). Synthesis, molecular modeling, and biological evaluation of novel RAD51 inhibitors. European J. Med. Chem..

[284] Huang F., Goyal N., Sullivan K., Hanamshet K., Patel M., Mazina O.M., Wang C.X., An W.F., Spoonamore J., Metkar S. (2016). Targeting BRCA1- and BRCA2-deficient cells with RAD52 small molecule inhibitors. Nucleic Acids Res..

[285] Sullivan K., Cramer-Morales K., McElroy D.L., Ostrov D.A., Haas K., Childers W., Hromas R., Skorski T. (2016). Identification of a Small Molecule Inhibitor of RAD52 by Structure-Based Selection. PLoS ONE.

[286] Hengel S.R., Malacaria E., Folly da Silva Constantino L., Bain F.E., Diaz A., Koch B.G., Yu L., Wu M., Pichierri P., Spies M.A. (2016). Small-molecule inhibitors identify the RAD52-ssDNA interaction as critical for recovery from replication stress and for survival of BRCA2 deficient cells. eLife Sci..

[287] Chandramouly G., McDevitt S., Sullivan K., Kent T., Luz A., Glickman J.F., Andrake M., Skorski T., Pomerantz R.T. (2015). Small-Molecule Disruption of RAD52 Rings as a Mechanism for Precision Medicine in BRCA-Deficient Cancers. Chem. Biol..

[288] Deakyne J.S., Huang F., Negri J., Tolliday N., Cocklin S., Mazin A.V. (2013). Analysis of the activities of RAD54, a SWI2/SNF2 protein, using a specific small-molecule inhibitor. J. Biol. Chem..

[289] Ambaye N., Chen C.-H., Khanna S., Li Y.-J., Chen Y. (2018). Streptonigrin Inhibits SENP1 and Reduces the Protein Level of Hypoxia-Inducible Factor 1α (HIF1α) in Cells. Biochemistry.

[290] Petermann E., Orta M.L., Issaeva N., Schultz N., Helleday T. (2010). Hydroxyurea-stalled replication forks become progressively inactivated and require two different RAD51-mediated pathways for restart and repair. Mol. Cell.

[291] Klein H.L. (2008). The consequences of Rad51 overexpression for normal and tumor cells. DNA Repair (Amst.).

[292] Richardson C., Stark J.M., Ommundsen M., Jasin M. (2004). Rad51 overexpression promotes alternative double-strand break repair pathways and genome instability. Oncogene.

[293] Raderschall E., Stout K., Freier S., Suckow V., Schweiger S., Haaf T. (2002). Elevated levels of Rad51 recombination protein in tumor cells. Cancer Res..

[294] Huang F., Motlekar N.A., Burgwin C.M., Napper A.D., Diamond S.L., Mazin A.V. (2011). Identification of specific inhibitors of human RAD51 recombinase using high-throughput screening. ACS Chem. Biol..

[295] Huang F., Mazin A.V. (2014). A small molecule inhibitor of human RAD51 potentiates breast cancer cell killing by therapeutic agents in mouse xenografts. PLoS ONE.

[296] Alagpulinsa D.A., Ayyadevara S., Shmookler Reis R.J. (2014). A Small-Molecule Inhibitor of RAD51 Reduces Homologous Recombination and Sensitizes Multiple Myeloma Cells to Doxorubicin. Front. Oncol..

[297] Mason J.M., Logan H.L., Budke B., Wu M., Pawlowski M., Weichselbaum R.R., Kozikowski A.P., Bishop D.K., Connell P.P. (2014). The RAD51-Stimulatory Compound RS-1 Can Exploit the RAD51 Overexpression That Exists in Cancer Cells and Tumors. Cancer Res..

[298] Yoshida K., Miki Y. (2004). Role of BRCA1 and BRCA2 as regulators of DNA repair, transcription, and cell cycle in response to DNA damage. Cancer Sci..

[299] Pellegrini L., Yu D.S., Lo T., Anand S., Lee M., Blundell T.L., Venkitaraman A.R. (2002). Insights into DNA recombination from the structure of a RAD51-BRCA2 complex. Nature.

[300] Carreira A., Kowalczykowski S.C. (2011). Two classes of BRC repeats in BRCA2 promote RAD51 nucleoprotein filament function by distinct mechanisms. Proc. Natl. Acad. Sci. USA.

[301] Zhu J., Zhou L., Wu G., Konig H., Lin X., Li G., Qiu X.L., Chen C.F., Hu C.M., Goldblatt E. (2013). A novel small molecule RAD51 inactivator overcomes imatinib-resistance in chronic myeloid leukaemia. EMBO Mol. Med..

[302] Mazina O.M., Keskin H., Hanamshet K., Storici F., Mazin A.V. (2017). Rad52 Inverse Strand Exchange Drives RNA-Templated DNA Double-Strand Break Repair. Mol. Cell.

[303] Lok B.H., Carley A.C., Tchang B., Powell S.N. (2013). RAD52 inactivation is synthetically lethal with deficiencies in BRCA1 and PALB2 in addition to BRCA2 through RAD51-mediated homologous recombination. Oncogene.

[304] Feng Z., Scott S.P., Bussen W., Sharma G.G., Guo G., Pandita T.K., Powell S.N. (2011). Rad52 inactivation is synthetically lethal with BRCA2 deficiency. Proc. Natl. Acad. Sci. USA.

[305] Pomerantz R.T. (2017). Abstract B39: Small Molecule Disruption of RAD52 Rings as a Mechanism for Precision Medicine in BRCA Deficient Cancers. Am. Assoc. Cancer Res..

[306] Mazin A.V., Mazina O.M., Bugreev D.V., Rossi M.J. (2010). Rad54, the motor of homologous recombination. DNA Repair (Amst.).

[307] Bolzán A.D., Bianchi M.S. (2001). Genotoxicity of streptonigrin: A review. Mutat. Res..

[308] Jiang C., Pugh B.F. (2009). Nucleosome positioning and gene regulation: Advances through genomics. Nat. Rev. Genet..

[309] McGinty R.K., Tan S. (2015). Nucleosome structure and function. Chem. Rev..

[310] McAnena P., Brown J.A., Kerin M.J. (2017). Circulating Nucleosomes and Nucleosome Modifications as Biomarkers in Cancer. Cancers (Basal).

[311] Shogren-Knaak M., Ishii H., Sun J.M., Pazin M.J., Davie J.R., Peterson C.L. (2006). Histone H4-K16 acetylation controls chromatin structure and protein interactions. Science.

[312] Zentner G.E., Henikoff S. (2013). Regulation of nucleosome dynamics by histone modifications. Nat. Struct. Mol. Biol..

[313] Allis C.D., Jenuwein T. (2016). The molecular hallmarks of epigenetic control. Nat. Rev. Genet..

[314] Banerjee T., Chakravarti D. (2011). A peek into the complex realm of histone phosphorylation. Mol. Cell. Biol..

[315] Brownell J.E., Allis C.D. (1996). Special HATs for special occasions: Linking histone acetylation to chromatin assembly and gene activation. Curr. Opin. Genet. Dev..

[316] Kouzarides T. (2007). Chromatin modifications and their function. Cell.

[317] Price B.D., D’Andrea A.D. (2013). Chromatin remodeling at DNA double-strand breaks. Cell.

[318] Baylin S.B., Esteller M., Rountree M.R., Bachman K.E., Schuebel K., Herman J.G. (2001). Aberrant patterns of DNA methylation, chromatin formation and gene expression in cancer. Hum. Mol. Genet..

[319] Jenuwein T., Allis C.D. (2001). Translating the histone code. Science.

[320] Hunt C.R., Ramnarain D., Horikoshi N., Iyengar P., Pandita R.K., Shay J.W., Pandita T.K. (2013). Histone modifications and DNA double-strand break repair after exposure to ionizing radiations. Radiat. Res..

[321] Corpet A., Almouzni G. (2009). A histone code for the DNA damage response in mammalian cells?. EMBO J..

[322] Bennetzen M.V., Larsen D.H., Dinant C., Watanabe S., Bartek J., Lukas J., Andersen J.S. (2013). Acetylation dynamics of human nuclear proteins during the ionizing radiation-induced DNA damage response. Cell Cycle.

[323] Brown J.A.L., Bourke E., Eriksson L.A., Kerin M.J. (2016). Targeting cancer using KAT inhibitors to mimic lethal knockouts. Biochem. Soc. Trans..

[324] Conte M., Altucci L. (2012). Molecular pathways: The complexity of the epigenome in cancer and recent clinical advances. Clin. Cancer Res..

[325] Gong F., Chiu L.-Y., Miller K.M. (2016). Acetylation Reader Proteins: Linking Acetylation Signaling to Genome Maintenance and Cancer. PLoS Genet..

[326] Bertrand P. (2010). Inside HDAC with HDAC inhibitors. Eur. J. Med. Chem..

[327] Filippakopoulos P., Knapp S. (2014). Targeting bromodomains: Epigenetic readers of lysine acetylation. Nat. Rev. Drug Discov..

[328] De Ruijter A.J.M., van Gennip A.H., Caron H.N., Kemp S., van Kuilenburg A.B.P. (2003). Histone deacetylases (HDACs): Characterization of the classical HDAC family. Biochem. J..

[329] Inche A.G., La Thangue N.B. (2006). Chromatin control and cancer-drug discovery: Realizing the promise. Drug Discov. Today.

[330] Miller T.A., Witter D.J., Belvedere S. (2003). Histone deacetylase inhibitors. J. Med. Chem..

[331] Zhang C., Richon V., Ni X., Talpur R., Duvic M. (2005). Selective induction of apoptosis by histone deacetylase inhibitor SAHA in cutaneous T-cell lymphoma cells: Relevance to mechanism of therapeutic action. J. Investig. Dermatol..

[332] Xu W.S., Parmigiani R.B., Marks P.A. (2007). Histone deacetylase inhibitors: Molecular mechanisms of action. Oncogene.

[333] Madsen A.S., Kristensen H.M.E., Lanz G., Olsen C.A. (2014). The effect of various zinc binding groups on inhibition of histone deacetylases 1-11. ChemMedChem.

[334] Carey N., La Thangue N.B. (2006). Histone deacetylase inhibitors: Gathering pace. Curr. Opin. Pharmacol..

[335] Khan O., La Thangue N.B. (2012). HDAC inhibitors in cancer biology: Emerging mechanisms and clinical applications. Immunol. Cell Biol..

[336] Kelly W.K., Marks P.A. (2005). Drug insight: Histone deacetylase inhibitors—Development of the new targeted anticancer agent suberoylanilide hydroxamic acid. Nat. Clin. Pract. Oncol..

[337] Zagni C., Floresta G., Monciino G., Rescifina A. (2017). The Search for Potent, Small-Molecule HDACIs in Cancer Treatment: A Decade after Vorinostat. Med. Res. Rev..

[338] Mai A., Altucci L. (2009). Epi-drugs to fight cancer: From chemistry to cancer treatment, the road ahead. Int. J. Biochem. Cell Biol..

[339] Curtin M.L., Garland R.B., Heyman H.R., Frey R.R., Michaelides M.R., Li J., Pease L.J., Glaser K.B., Marcotte P.A., Davidsen S.K. (2002). Succinimide hydroxamic acids as potent inhibitors of histone deacetylase (HDAC). Bioorg. Med. Chem. Lett..

[340] Marks P.A. (2007). Discovery and development of SAHA as an anticancer agent. Oncogene.

[341] Li D., Marchenko N.D., Moll U.M. (2011). SAHA shows preferential cytotoxicity in mutant p53 cancer cells by destabilizing mutant p53 through inhibition of the HDAC6-Hsp90 chaperone axis. Cell Death Differ..

[342] Kelly A.D., Issa J.-P.J. (2017). The promise of epigenetic therapy: Reprogramming the cancer epigenome. Curr. Opin. Genet. Dev..

[343] Bennett R.L., Licht J.D. (2018). Targeting Epigenetics in Cancer. Annu. Rev. Pharmacol. Toxicol..

[344] Jacquet K., Fradet-Turcotte A., Avvakumov N., Lambert J.P., Roques C., Pandita R.K., Paquet E., Herst P., Gingras A.C., Pandita T.K. (2016). The TIP60 Complex Regulates Bivalent Chromatin Recognition by 53BP1 through Direct H4K20me Binding and H2AK15 Acetylation. Mol. Cell.

[345] VanderMolen K.M., McCulloch W., Pearce C.J., Oberlies N.H. (2011). Romidepsin (Istodax, NSC 630176, FR901228, FK228, depsipeptide): A natural product recently approved for cutaneous T-cell lymphoma. J. Antibiot..

[346] Zhao S., Guo J., Zhao Y., Fei C., Zheng Q., Li X., Chang C. (2016). Chidamide, a novel histone deacetylase inhibitor, inhibits the viability of MDS and AML cells by suppressing JAK2/STAT3 signaling. Am. J. Transl. Res..

[347] Shi Y., Dong M., Hong X., Zhang W., Feng J., Zhu J., Yu L., Ke X., Huang H., Shen Z. (2015). Results from a multicenter, open-label, pivotal phase II study of chidamide in relapsed or refractory peripheral T-cell lymphoma. Ann. Oncol..

[348] Yardley D.A., Ismail-Khan R.R., Melichar B., Lichinitser M., Munster P.N., Klein P.M., Cruickshank S., Miller K.D., Lee M.J., Trepel J.B. (2013). Randomized phase II, double-blind, placebo-controlled study of exemestane with or without entinostat in postmenopausal women with locally recurrent or metastatic estrogen receptor-positive breast cancer progressing on treatment with a nonsteroidal aromatase inhibitor. J. Clin. Oncol..

[349] Damaskos C., Garmpis N., Valsami S., Kontos M., Spartalis E., Kalampokas T., Kalampokas E., Athanasiou A., Moris D., Daskalopoulou A. (2017). Histone Deacetylase Inhibitors: An Attractive Therapeutic Strategy against Breast Cancer. Anticancer Res..

[350] Gottlicher M., Minucci S., Zhu P., Krämer O.H., Schimpf A., Giavara S., Sleeman J.P., Lo Coco F., Nervi C., Pelicci P.G. (2001). Valproic acid defines a novel class of HDAC inhibitors inducing differentiation of transformed cells. EMBO J..

[351] Wagner J.M., Hackanson B., Lübbert M., Jung M. (2010). Histone deacetylase (HDAC) inhibitors in recent clinical trials for cancer therapy. Clin. Epigenet..

[352] Mann B.S., Johnson J.R., Cohen M.H., Justice R., Pazdur R. (2007). FDA approval summary: Vorinostat for treatment of advanced primary cutaneous T-cell lymphoma. Oncologist.

[353] Lee H.-Z., Kwitkowski V.E., Del Valle P.L., Ricci M.S., Saber H., Habtemariam B.A., Bullock J., Bloomquist E., Li S.Y., Chen X.H. (2015). FDA Approval: Belinostat for the Treatment of Patients with Relapsed or Refractory Peripheral T-cell Lymphoma. Clin. Cancer Res..

[354] Atadja P. (2009). Development of the pan-DAC inhibitor panobinostat (LBH589): Successes and challenges. Cancer Lett..

[355] Barbarotta L., Hurley K. (2015). Romidepsin for the Treatment of Peripheral T-Cell Lymphoma. J. Adv. Pract. Oncol..

[356] Chan T., Tse E., Kwong Y.-L. (2017). Chidamide in the treatment of peripheral T-cell lymphoma. OTT.

[357] Choi K.-C., Jung M.G., Lee Y.H., Yoon J.C., Kwon S.H., Kang H.B., Kim M.J., Cha J.H., Kim Y.J., Jun W.J. (2009). Epigallocatechin-3-gallate, a histone acetyltransferase inhibitor, inhibits EBV-induced B lymphocyte transformation via suppression of RelA acetylation. Cancer Res..

[358] Lee Y.-H., Kwak J., Choi H.K., Choi K.C., Kim S., Lee J., Jun W., Park H.J., Yoon H.G. (2012). EGCG suppresses prostate cancer cell growth modulating acetylation of androgen receptor by anti-histone acetyltransferase activity. Int. J. Mol. Med..

[359] Balasubramanyam K., Varier R.A., Altaf M., Swaminathan V., Siddappa N.B., Ranga U., Kundu T.K. (2004). Curcumin, a novel p300/CREB-binding protein-specific inhibitor of acetyltransferase, represses the acetylation of histone/nonhistone proteins and histone acetyltransferase-dependent chromatin transcription. J. Biol. Chem..

[360] Gao C., Bourke E., Scobie M., Famme M.A., Koolmeister T., Helleday T., Eriksson L.A., Lowndes N.F., Brown J.A. (2014). Rational design and validation of a Tip60 histone acetyltransferase inhibitor. Sci. Rep..

[361] Coffey K., Blackburn T.J., Cook S., Golding B.T., Griffin R.J., Hardcastle I.R., Hewitt L., Huberman K., McNeill H.V., Newell D.R. (2012). Characterisation of a Tip60 Specific Inhibitor, NU9056, in Prostate Cancer. PLoS ONE.

[362] Gajer J.M., Furdas S.D., Gründer A., Gothwal M., Heinicke U., Keller K., Colland F., Fulda S., Pahl H.L., Fichtner I. (2015). Histone acetyltransferase inhibitors block neuroblastoma cell growth in vivo. Oncogenesis.

[363] Trisciuoglio D., Ragazzoni Y., Pelosi A., Desideri M., Carradori S., Gabellini C., Maresca G., Nescatelli R., Secci D., Bolasco A. (2012). CPTH6, a thiazole derivative, induces histone hypoacetylation and apoptosis in human leukemia cells. Clin. Cancer Res..

[364] Ravindra K.C., Selvi B.R., Arif M., Reddy B.A., Thanuja G.R., Agrawal S., Pradhan S.K., Nagashayana N., Dasgupta D., Kundu T.K. (2009). Inhibition of lysine acetyltransferase KAT3B/p300 activity by a naturally occurring hydroxynaphthoquinone, plumbagin. J. Biol. Chem..

[365] Daigle S.R., Olhava E.J., Therkelsen C.A., Basavapathruni A., Jin L., Boriack-Sjodin P.A., Allain C.J., Klaus C.R., Raimondi A., Scott M.P. (2013). Potent inhibition of DOT1L as treatment of MLL-fusion leukemia. Blood.

[366] Vedadi M., Barsyte-Lovejoy D., Liu F., Rival-Gervier S., Allali-Hassani A., Labrie V., Wigle T.J., Dimaggio P.A., Wasney G.A., Siarheyeva A. (2011). A chemical probe selectively inhibits G9a and GLP methyltransferase activity in cells. Nat. Chem. Biol..

[367] Knutson S.K., Kawano S., Minoshima Y., Warholic N.M., Huang K.C., Xiao Y., Kadowaki T., Uesugi M., Kuznetsov G., Kumar N. (2014). Selective inhibition of EZH2 by EPZ-6438 leads to potent antitumor activity in EZH2-mutant non-Hodgkin lymphoma. Mol. Cancer Ther..

[368] Ferguson A.D., Larsen N.A., Howard T., Pollard H., Green I., Grande C., Cheung T., Garcia-Arenas R., Cowen S., Wu J. (2011). Structural basis of substrate methylation and inhibition of SMYD2. Structure.

[369] Williams D.E., Dalisay D.S., Li F., Amphlett J., Maneerat W., Chavez M.A., Wang Y.A., Matainaho T., Yu W., Brown P.J. (2013). Nahuoic acid A produced by a Streptomyces sp. isolated from a marine sediment is a selective SAM-competitive inhibitor of the histone methyltransferase SETD8. Org. Lett..

[370] Judge R.A., Zhu H., Upadhyay A.K., Bodelle P.M., Hutchins C.W., Torrent M., Marin V.L., Yu W., Vedadi M., Li F. (2016). Turning a Substrate Peptide into a Potent Inhibitor for the Histone Methyltransferase SETD8. ACS Med. Chem. Lett..

[371] Schmidt D.M.Z., McCafferty D.G. (2007). trans-2-Phenylcyclopropylamine is a mechanism-based inactivator of the histone demethylase LSD1. Biochemistry.

[372] Mohammad H.P., Kruger R.G. (2016). Antitumor activity of LSD1 inhibitors in lung cancer. Mol. Cell Oncol..

[373] Carrozza M.J., Utley R.T., Workman J.L., Cote J. (2003). The diverse functions of histone acetyltransferase complexes. Trends Genet..

[374] Farria A., Li W., Dent S.Y.R. (2015). KATs in cancer: Functions and therapies. Oncogene.

[375] Wu J., Xie N., Wu Z., Zhang Y., Zheng Y.G. (2009). Bisubstrate Inhibitors of the MYST HATs Esa1 and Tip60. Bioorg. Med. Chem..

[376] Simon R.P., Robaa D., Alhalabi Z., Sippl W., Jung M. (2016). KATching-Up on Small Molecule Modulators of Lysine Acetyltransferases. J. Med. Chem..

[377] Koeller K.M., Haggarty S.J., Perkins B.D., Leykin I., Wong J.C., Kao M.C., Schreiber S.L. (2003). Chemical genetic modifier screens: Small molecule trichostatin suppressors as probes of intracellular histone and tubulin acetylation. Chem. Biol..

[378] Ghizzoni M., Wu J., Gao T., Haisma H.J., Dekker F.J., Zheng Y.G. (2012). 6-alkylsalicylates are selective Tip60 inhibitors and target the acetyl-CoA binding site. Eur. J. Med. Chem..

[379] Furdas S.D., Kannan S., Sippl W., Jung M. (2012). Small molecule inhibitors of histone acetyltransferases as epigenetic tools and drug candidates. Arch. Pharm. (Weinh.).

[380] Dawson M.A. (2017). The cancer epigenome: Concepts, challenges, and therapeutic opportunities. Science.

[381] Di Martile M., Desideri M., De Luca T., Gabellini C., Buglioni S., Eramo A., Sette G., Milella M., Rotili D., Mai A. (2016). Histone acetyltransferase inhibitor CPTH6 preferentially targets lung cancer stem-like cells. Oncotarget.

[382] Murray K. (1964). The Occurrence Of Epsilon-N-Methyl Lysine In Histones. Biochemistry.

[383] Black J.C., Van Rechem C., Whetstine J.R. (2012). Histone lysine methylation dynamics: Establishment, regulation, and biological impact. Mol. Cell.

[384] Yang Y., Bedford M.T. (2013). Protein arginine methyltransferases and cancer. Nat. Rev. Cancer.

[385] Gong F., Miller K.M. (2017). Histone methylation and the DNA damage response. Mutat. Res./Rev. Mutat. Res..

[386] Bakkenist C.J., Kastan M.B. (2015). Chromatin perturbations during the DNA damage response in higher eukaryotes. DNA Repair (Amst.).

[387] Dawson M.A., Kouzarides T. (2012). Cancer epigenetics: From mechanism to therapy. Cell.

[388] Højfeldt J.W., Agger K., Helin K. (2013). Histone lysine demethylases as targets for anticancer therapy. Nat. Rev. Drug Discov..

[389] Yao Y., Chen P., Diao J., Cheng G., Deng L., Anglin J.L., Prasad B.V., Song Y. (2011). Selective inhibitors of histone methyltransferase DOT1L: Design, synthesis, and crystallographic studies. J. Am. Chem. Soc..

[390] Daigle S.R., Olhava E.J., Therkelsen C.A., Majer C.R., Sneeringer C.J., Song J., Johnston L.D., Scott M.P., Smith J.J., Xiao Y. (2011). Selective killing of mixed lineage leukemia cells by a potent small-molecule DOT1L inhibitor. Cancer Cell.

[391] Bernt K.M., Zhu N., Sinha A.U., Vempati S., Faber J., Krivtsov A.V., Feng Z., Punt N., Daigle A., Bullinger L. (2011). MLL-rearranged leukemia is dependent on aberrant H3K79 methylation by DOT1L. Cancer Cell.

[392] Song Y., Li L., Chen Y., Liu J., Xiao S., Lian F., Zhang N., Ding H., Zhang Y., Chen K. (2018). Discovery of potent DOT1L inhibitors by AlphaLISA based High Throughput Screening assay. Bioorg. Med. Chem..

[393] Dillon S.C., Zhang X., Trievel R.C., Cheng X. (2005). The SET-domain protein superfamily: Protein lysine methyltransferases. Genome Biol..

[394] Botuyan M.V., Lee J., Ward I.M., Kim J.E., Thompson J.R., Chen J., Mer G. (2006). Structural basis for the methylation state-specific recognition of histone H4-K20 by 53BP1 and Crb2 in DNA repair. Cell.

[395] Takawa M., Cho H.S., Hayami S., Toyokawa G., Kogure M., Yamane Y., Iwai Y., Maejima K., Ueda K., Masuda A. (2012). Histone lysine methyltransferase SETD8 promotes carcinogenesis by deregulating PCNA expression. Cancer Res..

[396] Ma A., Yu W., Li F., Bleich R.M., Herold J.M., Butler K.V., Norris J.L., Korboukh V., Tripathy A., Janzen W.P. (2014). Discovery of a selective, substrate-competitive inhibitor of the lysine methyltransferase SETD8. J. Med. Chem..

[397] Zheng Y.-C., Yu B., Chen Z.-S., Liu Y., Liu H.-M. (2016). TCPs: Privileged scaffolds for identifying potent LSD1 inhibitors for cancer therapy. Epigenomics.

[398] Ferrari-Amorotti G., Chiodoni C., Shen F., Cattelani S., Soliera A.R., Manzotti G., Grisendi G., Dominici M., Rivasi F., Colombo M.P. (2014). Suppression of invasion and metastasis of triple-negative breast cancer lines by pharmacological or genetic inhibition of slug activity. Neoplasia.

[399] Hosseini A., Minucci S. (2017). A comprehensive review of lysine-specific demethylase 1 and its roles in cancer. Epigenomics.

